# Regional variations in cardiovascular risk predictions: a comparative analysis of Framingham, SCORE2, and WHO models across 53 countries

**DOI:** 10.7189/jogh.15.04323

**Published:** 2025-12-19

**Authors:** Yetong Liu, Wenming Bian, Sidong Li, Zhe Lv, Yizhen Lyu, Jiaheng Zhang, Kangyu Chen, Hui Yang, Tao Chen, Chao Li

**Affiliations:** 1 Department of Epidemiology and Health Statistics, Xi’an Jiaotong University Health Science Center, Xi’an, Shaanxi, China; 2 Institute of Public Health Sciences, Division of life sciences and medicine, University of Science and Technology of China, Hefei, China; 3 Xi’an Children's Hospital, Xi’an, Shaanxi, China; 4 Department of Cardiology, The First Affiliated Hospital of USTC, Division of Life Sciences and Medicine, University of Science and Technology of China, Hefei, China; 5 Department of Clinical Sciences, Liverpool School of Tropical Medicine, Liverpool, UK; 6 Key Laboratory of Environment and Genes Related to Diseases Ministry of Education, Xi’an Jiaotong University, Xi’an, Shaanxi, China

## Abstract

**Background:**

Risk prediction models for cardiovascular diseases (CVDs) have been widely applied in clinical practice and in designing prevention policies globally, yet their accuracy across different regions with distinct epidemiological profiles remains uncertain. We examined the regional variation in risk distribution and agreement between these models.

**Methods:**

We analysed 53 nationally representative health surveys in seven regions. Using the World Health Organization (WHO), SCORE2, and Framingham CVD risk prediction models, we estimated the respondents’ 10-year CVD risk and categorised them into low-, moderate-, or high-risk groups.

**Results:**

We included 86 430 individuals aged 40–69 years without a history of CVD in our analysis. Globally, CVD risk estimates differed substantially across models (WHO: 7.75%; 95% confidence interval (CI) = 7.70–7.80; SCORE2: 3.72%; 95% CI = 3.69–3.75; Framingham: 12.42%; 95% CI = 12.34–12.50). We also noted regional disparities in identifying moderate- and high-risk subjects, particularly in South Asia (WHO: 12.57%; 95% CI = 11.63–13.51; SCORE2: 18.24%; 95% CI = 17.14–19.33; Framingham: 29.40%; 95% CI = 28.11–30.70), sub-Saharan Africa (WHO: 16.30%; 95% CI = 15.78–16.83; SCORE2: 22.69%; 95% CI = 22.09–23.28; Framingham: 33.85%; 95% CI = 33.18–34.52), East Asia & the Pacific (WHO: 21.06%; 95% CI = 20.57, 21.55; SCORE2: 31.03%; 95% CI = 30.47, 31.59; Framingham: 45.54%; 95% CI = 44.93–46.14), and Latin America & the Caribbean (WHO: 23.09%; 95% CI = 21.48–24.70; SCORE2: 41.56%; 95% CI = 39.68–43.44; Framingham: 55.83%; 95% CI = 53.94–57.72), with greater than two-fold differences across models. Agreement in classifying individuals into low-, moderate-, or high-risk groups remained relatively high across risk models (63.1%), but varied considerably across regions, from 73.91% in South Asia to 47.54% in Latin America & the Caribbean.

**Conclusions:**

The CVD risk estimates produced by the WHO, SCORE2, and Framingham models varied significantly across regions, with poor consistency in identifying at-risk individuals in some regions. These discrepancies may lead to undertreatment and inefficient use of otherwise limited healthcare resources. Region-specific adaptations are needed to enhance risk targeting, promote equity, and improve the overall effectiveness of primary prevention.

Cardiovascular disease (CVD) is the leading cause of death and disability worldwide [1,2]. Given this global impact, models that estimate individual CVD risk are highly important for clinical decision-making, as well as informing targeted prevention efforts and primary prevention initiatives [3–5]. Many such models have been developed and applied in practice, with the Framingham, SCORE2, and World Health Organization (WHO) models being used widely in practice and health policy [4,6,7]. However, these three models were primarily derived from data on the populations of Europe and North America [8–10], limiting their applicability to other contexts. This lack of data on the accuracy of these models in regions with different epidemiological profiles may lead to the misclassification of risk in their populations, thereby hindering the development of effective, region-specific interventions [11]. To address this limitation, we applied the Framingham, SCORE2, and WHO models to estimate 10-year CVD risk across 53 countries spanning seven global regions and thus evaluate the divergences and agreements in their risk classification.

## METHODS

### Study data

We based our analysis on individual-level data from national health surveys. To ensure consistency and representativeness, we primarily used nationally representative datasets from the WHO Stepwise Approach to Surveillance (STEPS) survey, a standardised framework that collects behavioural, physical and biochemical risk factor data from adults [12]. For countries where valid STEPS data could not be obtained, we retrieved the data from the latest nationally representative surveys, including NHANES (USA), ELSA (UK), HAALSI (South Africa), MHAS (Mexico), CRELES (Costa Rica), MIDJA (Japan), and CHARLS (Figure S1, Table S1, and Text S1 in the **Online Supplementary Document**). After this process, we were left with data from 53 countries, which we into seven geographic regions based on the World Bank Classification [13]: East Asia & the Pacific, Europe & Central Asia, Latin America & the Caribbean, Middle East & North Africa, North America, South Asia, and sub-Saharan Africa 

We only included datasets that contained the following key variables for risk prediction models: gender, age, systolic blood pressure, total cholesterol, high-density lipoprotein, smoking history, diabetes history, hypertension treatment, and prior cardiovascular disease. We excluded participants with a history of CVD or with missing data required for risk estimation. To ensure consistency, we further restricted the analysis to adults aged 40–69 years, as this age group bears a high cardiovascular disease burden [9], and it overlaps fully with the age recommended for SCORE2 (40–69 years), and partially with the one required by the Framingham (20–79 years) and WHO (40–80 years) age groups [8,10].

### Cardiovascular risk prediction models

The Framingham model, developed in the Framingham Heart Study, is a multivariable risk prediction tool designed to estimate the 10-year risk of developing CVD [8]. It incorporates key risk factors, including age, total cholesterol, HDL cholesterol levels, systolic blood pressure (with differentiation based on antihypertensive treatment), smoking status, and diabetes. The SCORE2 model [9], meanwhile, is tailored for European populations aged 40–69 years and is designed to predict the 10-year risk of a first CVD event. It includes age, smoking status, systolic blood pressure, total cholesterol, and HDL cholesterol as the main risk factors. The WHO model [10] similarly estimates the 10-year CVD risk, but does so across 21 global regions and includes age, smoking status, systolic blood pressure, diabetes history, and total cholesterol as key predictors. Evidence from original studies and subsequent validations indicates shows acceptable discrimination and calibration for cardiovascular risk prediction for all three models [8–10] (Table S2 in the **Online Supplementary Document**).

### Data analysis

After standardising them across databases to ensure consistent definitions and units, we harmonised and integrated common variables such as age, gender, systolic blood pressure, total cholesterol, high-density lipoprotein cholesterol, smoking history, diabetes history, and hypertension treatment (Text S2 in the **Online Supplementary Document**). We estimated the Framingham CVD risk score using the ‘framingham’ command in Stata [8]. The SCORE2 CVD risk score was calculated based on a pre-defined methodology, without regional adjustments [9]. Specifically, we used an uncalibrated SCORE2 model in all analyses, as the risk factors prescribed within the aforementioned methodology are calibrated for European countries and may not generalise, or may even reduce predictive performance [9,14]. Lastly, we computed the WHO CVD risk score using the ‘whocvdrisk’ command in Stata, with national adjustments applied to the risk scores [10].

We categorised the CVD risk scores from the three models into low, moderate, and high-risk groups, using thresholds defined in the original studies or widely applied in prior research [15]. The original Framingham study did not provide exact thresholds [8]; however, <10%, 10–20%, and ≥20% are commonly applied in validation studies and recommended by clinical guidelines [16–18]. Although the original charts for the WHO model defined five categories, we followed prior studies in using <10%, 10–20%, and ≥20% to represent low, moderate, and high risk for comparability [19]. For SCORE2, we used the original age-specific cut-offs [9]: for individuals aged <50 years, thresholds of <2.5%, 2.5–7.5%, and ≥7.5% indicated low, moderate, and high risk, respectively; for those aged 50–69 years, these thresholds were set at <5%, 5–10%, and ≥10%, respectively. The thresholds used to define high-risk individuals also correspond to the guideline-recommended station initiation thresholds underlying each model [5,18,20].

### Sensitivity analysis

To address concerns about age restriction, we expanded the analytic sample to adults aged 30–80 years and applied the Framingham, SCORE2, and WHO models across this full age range, thereby ensuring that all models were evaluated on an identical population. To account for missing data in model covariates, we applied multiple imputation and re-ran the main analyses using the imputed data sets. To examine the impact of regional recalibration, we repeated the analyses without applying any regional adjustment to either the WHO or the SCORE2 models.

We performed all analyses in Stata, version 18.0 (StataCorp LLC, College Station, TX, USA) and *R*, version 4.4.1 (R Foundation for Statistical Computing, Vienna, Austria). Our study adheres to the Journal of Global Health's Guidelines for Reporting Analyses of Big Data Repositories Open to the Public (Table S3 in the **Online Supplementary Document**).

## RESULTS

Of 292 264 individuals from 53 surveys across 7 global regions (Figure S1 in the **Online Supplementary Document**), we excluded 205 834 due to their history of CVD (n = 18 117), age restrictions (n = 132 642), missing data (n = 54 385), and outlier values (n = 690). The final sample included 86 430 individuals aged 40–69 years (mean age = 52.96 years, 41.22% male), of whom 26 174 (30.25%) were from East Asia & the Pacific, 19 071 (22.06%) from Sub-Saharan Africa, 17 014 (19.67%) from Europe & Central Asia, 14 497 (16.83%) from Middle East & North Africa, 4765 (5.51%) from South Asia, 2642 (3.05%) from Latin America & the Caribbean, and 2267 (2.63%) from North America.

**Figure 1:**
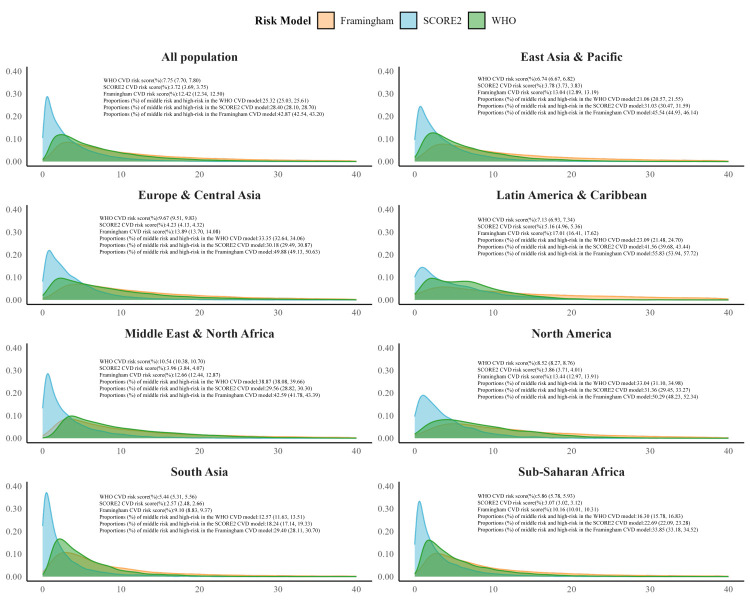
Variations in the density distribution of three CVD risk assessment models across regions.

Risk estimates varied considerably across models, with the Framingham estimating the highest risk (12.42%; 95% CI = 12.34–12.50), followed by the WHO (7.75%; 95% CI = 7.70–7.80) and the SCORE2 (3.72%; 95% CI = 3.69, 3.75). We likewise noted substantial regional variations in all models, with estimates ranging from 5.44% (95% CI = 5.31–5.56) in South Asia to 10.54% (95% CI = 10.38–10.70) in the Middle East & North Africa for the WHO model; 2.57% (95% CI = 2.48–2.66) in South Asia to 5.16% (95% CI = 4.96–5.36) in Latin America & the Caribbean for SCORE2, and 9.10% (95% CI = 8.83–9.37) in South Asia to 17.01% (95% CI = 16.41–17.62) in the Middle East & North Africa for Framingham (**Figure 1**, **Table 1**). Approximately 7.35% to 36.22% of individuals across regions were classified as high-risk by all three models. There was substantial cross-country heterogeneity at baseline in demographic profiles and cardiovascular risk factors, as well as gender differences, with women scoring lower across all models (Tables S4 and S5 in the **Online Supplementary Document**).

**Table 1:** Baseline characteristics of participants stratified by region*

	East Asia & the Pacific	Europe & Central Asia	Latin America & the Caribbean	Middle East & North Africa	North America	South Asia	Sub-Saharan Africa	Overall
**Sample size**	26 174	17 014	2642	14 497	2267	4765	19 071	86 430
**Age in years, x̄ (95% CI)**	53.19 (53.09–53.28)	54.92 (54.80–55.04)	57.15 (56.82–57.49)	51.35 (51.22–51.48)	55.01 (54.66–55.35)	50.89 (50.65–51.12)	51.84 (51.72–51.96)	52.97 (52.91–53.02)
**Age group**								
40–49	9554 (36.50)	4844 (28.47)	652 (24.68)	6766 (46.67)	675 (29.78)	2319 (48.67)	8111 (42.53)	32 921 (38.09)
50–59	10 013 (38.26)	6533 (38.40)	685 (25.93)	4868 (33.58)	745 (32.86)	1531 (32.13)	6578 (34.49)	30 953 (35.81)
60–69	6607 (25.24)	5637 (33.13)	1305 (49.39)	2863 (19.75)	847 (37.36)	915 (19.20)	4382 (22.98)	22 556 (26.10)
**Men**	11466 (43.81)	6616 (38.89)	965 (36.53)	5633 (38.86)	1076 (47.46)	2178 (45.71)	7697 (40.36)	35 631 (41.23)
**SBP (mmHg), x̄ (95% CI)**	129.59 (129.34–129.84)	139.02 (138.60–139.44)	137.34 (136.51–138.18)	133.71 (133.27–134.16)	128.92 (128.13–129.72)	131.12 (130.49–131.75)	134.63 (134.30–134.95)	133.55 (133.39–133.72)
**TC (mg/dl), x̄ (95% CI)**	184.93 (184.35–185.50)	193.73 (193.03–194.44)	192.27 (190.30–194.24)	180.91 (180.13–181.68)	196.89 (195.22–198.55)	177.23 (176.07–178.40)	156.89 (156.24–157.55)	179.91 (179.59–180.24)
**HDL (mg/dl), x̄ (95% CI)**	47.55 (47.30–47.80)	51.70 (51.44–51.96)	54.88 (54.12–55.63)	45.85 (45.60–46.10)	53.45 (52.79–54.12)	65.77 (64.99–66.56)	50.24 (49.89–50.59)	50.06 (49.92–50.20)
**Individuals who smoke**	7311 (27.93)	2900 (17.04)	382 (14.46)	2451 (16.91)	423 (18.66)	1247 (26.17)	2351 (12.33)	17 065 (19.74)
**Individuals with diabetes**	3881 (14.83)	1457 (8.56)	587 (22.22)	3313 (22.85)	419 (18.48)	575 (12.07)	3991 (20.93)	14 223 (16.46)
**Individuals receiving treatment for hypertension**	4296 (16.41)	3721 (21.87)	792 (29.98)	2965 (20.45)	731 (32.25)	487 (10.22)	2356 (12.35)	15 348 (17.76)
**% of WHO CVD risk score, x̄ (95% CI)**	6.74 (6.67–6.82)	9.67 (9.51–9.83)	7.13 (6.93–7.34)	10.54 (10.38–10.70)	8.52 (8.27–8.76)	5.44 (5.31–5.56)	5.86 (5.78–5.93)	7.75 (7.70–7.80)
**% of SCORE2 CVD risk score, x̄ (95% CI)**	3.78 (3.73–3.83)	4.23 (4.13–4.32)	5.16 (4.96–5.36)	3.96 (3.84–4.07)	3.86 (3.71–4.01)	2.57 (2.48–2.66)	3.07 (3.02–3.12)	3.72 (3.69–3.75)
**% of Framingham CVD risk score, x̄ (95% CI)**	13.04 (12.89–13.19)	13.89 (13.70–14.08)	17.01 (16.41–17.62)	12.66 (12.44–12.87)	13.44 (12.97–13.91)	9.10 (8.83–9.37)	10.16 (10.01–10.31)	12.42 (12.34–12.50)
**Individuals at moderate or high risk in the WHO CVD model**	5512 (21.06)	5674 (33.35)	610 (23.09)	5635 (38.87)	749 (33.04)	599 (12.57)	3109 (16.30)	21 888 (25.32)
**Individuals at moderate or high risk in the SCORE2 CVD model**	8122 (31.03)	5135 (30.18)	1098 (41.56)	4285 (29.56)	711 (31.36)	869 (18.24)	4327 (22.69)	24 547 (28.40)
**Individuals at moderate or high risk in the Framingham CVD model**	11 919 (45.54)	8487 (49.88)	1475 (55.83)	6174 (42.59)	1140 (50.29)	1401 (29.40)	6456 (33.85)	37 052 (42.87)
**Individuals with consistent high-risk**	746 (14.78)	1121 (30.33)	62 (7.35)	1032 (36.22)	98 (20.33)	56 (11.45)	366 (14.88)	3481 (21.60)

CI – confidence interval, CVD – cardiovascular disease, HDL – high-density lipoprotein cholesterol, SBP – systolic blood pressure, TC – total cholesterol

*Data are presented as n (%) unless specified otherwise.

**Figure 2:**
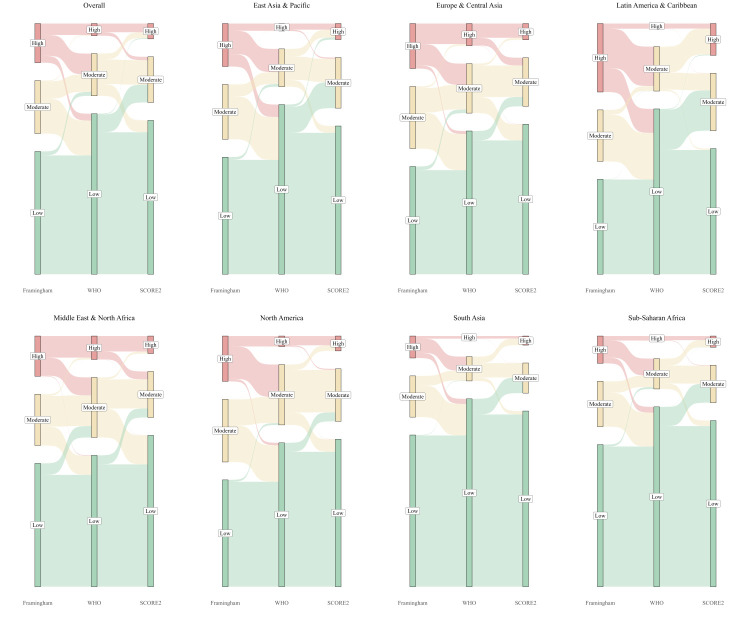
Distribution of 10-year cardiovascular disease risk levels across regions.

At a global level (**Figure 2**), the Framingham identified the highest proportion of individuals as moderate-to-high risk (42.87%; 95% CI = 42.54, 43.20), followed by the SCORE2 (28.40%; 95% CI = 28.10–28.70) and the WHO model (25.32%; 95% CI = 25.03–25.61). The differences between models were particularly pronounced in South Asia (WHO: 12.57%; 95% CI = 11.63–13.51; SCORE2: 18.24%; 95% CI = 17.14–19.33; Framingham: 29.40%; 95% CI = 28.11–30.70), sub-Saharan Africa (WHO: 16.30%; 95% CI = 15.78–16.83; SCORE2: 22.69%; 95% CI = 22.09–23.28; Framingham: 33.85%; 95% CI = 33.18–34.52), East Asia & the Pacific (WHO: 21.06%; 95% CI = 20.57–21.55; SCORE2: 31.03%; 95% CI = 30.47–31.59; Framingham: 45.54%; 95% CI = 44.93–46.14), and Latin America & the Caribbean (WHO: 23.09%; 95% CI = 21.48–24.70; SCORE2: 41.56%; 95% CI = 39.68–43.44; Framingham: 55.83%; 95% CI = 53.94–57.72), where there was a greater than two-fold variation in moderate-to-high risk classification across models. For gender sub-analyses, men were consistently classified as having higher risk than women across all three, while national-level explorations detected significant differences in countries such as Palau, Nepal, Uruguay, and Seychelles (Figures S2 and S3, and Tables S4 and S5 in the **Online Supplementary Document**).

**Figure 3:**
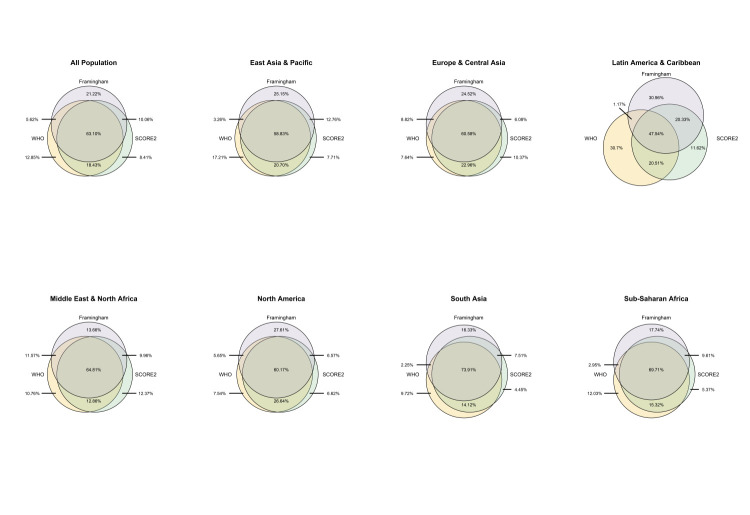
Consistency of three CVD risk assessment models in classifying low, moderate, and high-risk levels across regions.

The models showed relatively high consistency (63.1%) in classifying individuals as low-, moderate- or high-risk, albeit with notable regional differences, ranging from 47.54% in Latin America & the Caribbean to 73.91% in South Asia (**Figure 3**). These findings were supported by country-level analyses (Figure S4 in the **Online Supplementary Document**). Moreover, the models were more consistent in categorising younger individuals and those from lower economic backgrounds (Figures S5 and S6 in the **Online Supplementary Document**). The main findings remained robust in all sensitivity analyses (Figures S7–12 and Table S6 in the **Online Supplementary Document**)

## DISCUSSION

This investigation revealed significant variations in 10-year CVD risk estimates across models and regions. Notably, there was a greater than two-fold difference across models in the proportion of individuals classified as moderate- or high-risk in South Asia, sub-Saharan Africa, East Asia & the Pacific, and Latin America & the Caribbean. We likewise observed substantial variations in agreement in risk classification across regions, ranging from 73.91% in South Asia to 47.54% in Latin America & the Caribbean.

For decades, CVD has been the leading cause of mortality worldwide and a significant contributor to excess healthcare costs [1,21,22]. To facilitate CVD prevention and risk prediction, models such as Framingham, SCORE2, and WHO have been widely adopted across global regions [4,7]. However, most existing studies are single-country analyses, with few providing a cross-country or regional perspective [14,23,24]. This limitation arises because these models were primarily developed using data from European and North American populations, leading to limited representation of other regions [8–10]. When applied to populations from different regions with varying baseline risks, these models may experience calibration drift, resulting in systematic miscalibration. Further discrepancies among models stem from differences in outcome definitions, statistical frameworks, and risk factor inclusion [3,4,7]. For example, the WHO model excludes high-density lipoprotein and hypertension treatment, while the SCORE2 and Framingham incorporate heart failure outcomes, which are omitted in the WHO model [8–10]. Taken together, these omissions may partly contribute to the consistently lower risk estimates generated by the WHO model.

Existing research suggests that current CVD risk prediction models may not effectively distinguish moderate- and high-risk individuals [3,7]. Here, we found greater than two-fold variation in risk classification across models, particularly in South Asia, sub-Saharan Africa, East Asia & the Pacific, and Latin America & the Caribbean. These differences remained consistent across genders and national levels. We observed substantial variations even within the same model. South Asia and sub-Saharan Africa had the lowest proportions of moderate- to high-risk individuals, while North America, Europe & Central Asia had the highest, reflecting differences in regional epidemiological patterns and baseline characteristics, as shown in previous studies [25–27]. These disparities hinder precise risk stratification, which is essential for targeting preventive interventions such as statin therapy [5,28]. In South Asia, for example, only 11.45% of individuals were classified as high-risk by all three models. Given this population’s heightened susceptibility to statin-induced hyperglycaemia, such model inconsistency may lead to inappropriate statin use and increased diabetes risk [29,30]. In contrast, North America and Europe & Central Asia exhibited smaller discrepancies between our models, likely due to the extensive use of population cohorts from these regions in model development and validation [4,6,8–10]. Similarly, the relative alignment observed in the Middle East & North Africa may stem from its shared genetic [31,32] and epidemiological characteristics [21,25] with European populations. Given the higher morbidity and mortality risk among moderate- and high-risk individuals, improving risk assessment is crucial for designing effective interventions [4,5,33]. Accordingly, regions with the largest discrepancies in risk classification represent priority areas for validation studies and regional recalibration of the three models used in our study, as said discrepancies do not necessarily indicate that the models are invalid, but rather that tools developed in Western populations may be poorly adapted to certain regions [3,4,11]. Developing models tailored to these regions could, therefore, allow us to better capture local risk factors and epidemiological patterns, enhancing their predictive accuracy and clinical utility [11,15,34].

Our analysis further revealed a substantial degree of model consistency in risk classification within different regions, particularly in South Asia and sub-Saharan Africa. These results would coincide with our prior findings presented above. For example, the certain low risk observed in South Asia and sub-Saharan Africa suggests that most individuals from these regions were all classified as low risk across models. However, this is not necessarily indicative of model accuracy, as research suggests that individuals classified as low risk in the short term may still have a high long-term CVD risk [35]. Our findings further indicate that these models remain especially consistent in CVD risk classification among younger and lower-income populations. This may be due to the diminishing impact of common CVD risk factors with age, particularly in higher-income groups, where uncommon ones play a greater role [31,36–38]. Although current models incorporate age as a weighted variable, they do not explicitly account for the shifting impact of different risk factors across age groups [8–10], highlighting the need for age-specific risk assessment approaches [39–41].

Our study has several limitations. First, although the dataset we used was robust, the extent of missing covariates may have introduced selection bias. However, the sensitivity analyses we performed using multiple imputation produced results that were broadly consistent with the main findings. Second, although variations in data collection methods across countries may have introduced bias, the survey designs were largely comparable. Third, our analysis focussed on adults aged 40–69 years, limiting the generalisability of our findings to other age groups. However, the individuals within this age range are at elevated risk for cardiovascular disease [2,5], while our sensitivity analysis expanded to individuals aged 30–80 years yielded consistent results. Fourth, the lack of longitudinal outcome data precluded direct validation of model discrimination and calibration. We, therefore, focussed on comparing predicted risk distributions and model agreement to identify regions where validation and recalibration are most needed. Fifth, we did not apply the European region-specific calibration factors for SCORE2. While this may have biased absolute risk levels, it ensured consistency across regions and comparability with other models. Furthermore, sensitivity analyses for SCORE2 and WHO without regional recalibration produced results similar to the main analyses, indicating that the observed differences between models are unlikely to be solely due to regional calibration. Finally, although numerous CVD risk prediction models exist, we selected these three models based on their global adoption and reliance on readily available variables. While this also may have introduced bias in our estimates, the selection prioritised feasibility and comparability across diverse settings, particularly in low- and middle-income countries, where access to detailed clinical data may be limited [4].

## CONCLUSIONS

Our findings show regional variations in risk distribution and model agreement across seven regions, particularly in South Asia, sub-Saharan Africa, East Asia & the Pacific, and Latin America & the Caribbean. Refining these models based on region-specific data is crucial for improving risk prediction and designing targeted prevention strategies. Future research should strive to recalibrate the models with regionally- or nationally-specific cohorts, incorporate non-common risk factors, and adopt hybrid approaches using machine learning to improve their predictive performance and clinical utility.

## Additional material


Online Supplementary Document
