# Supplementary material for: Regional variations in cardiovascular risk predictions: a comparative analysis of Framingham, SCORE2, and WHO models across 53 countries
Source: J Glob Health. 2025 Dec 19;15:04323. doi: 10.7189/jogh.15.04323 (PMC12714358; doi:10.7189/jogh.15.04323)
Supplement: Online Supplementary Document [file jogh-15-04323-s001.pdf]

Supplement to: Liu Y, Bian W, Li S, Lv Z, Lyu Y, Zhang J, Chen K, Yang H, Chen T, Li C. Regional variations in cardiovascular risk predictions: a comparative analysis of Framingham, SCORE2, and WHO models across 53 countries. J Glob Health. 2025;15:04323.

Text S1. The search method for eligible surveys in countries where we were unable to acquire a WHO-STEPS survey 2

Text S2. Harmonization and Standardization of Variables Across National Surveys......3

TableS1. Population characteristics included in the databases for analysis......4

TableS2. Characteristics of cardiovascular risk assessment models used in the study 5

TableS3. GRABDROP compliance checklist for the present study......6

TableS4. Baseline characteristics of participants stratified by country......7

TableS5. Baseline characteristics of participants stratified by gender......10

TableS6. Global comparison of main and sensitivity analyses......11

Figure S1. Flow diagram of the search, screening, and selection process......12

Figure S2. Distribution of 10-year cardiovascular disease risk levels across regions and gender..13

Figure S3. Distribution of 10- year cardiovascular disease risk levels across regions and countries. 14

Figure S4. Pairwise consistency proportions of three CVD risk assessment models in classifying risk levels across regions and countries......21

Figure S5. Consistency of three CVD risk assessment models in classifying low, moderate, and high risk levels across age levels 28

Figure S6. Consistency of three CVD risk assessment models in classifying low, moderate, and high risk levels across income levels 29

Figure S7. Sensitivity analysis of the distribution of 10-year CVD risk levels across regions (age 30–80 years) 30

Figure S8.Sensitivity analysis of the consistency of three CVD risk assessment models in classifying low, moderate, and high risk levels across regions (age 30–80 years) 31

Figure S9. Sensitivity analysis of the distribution of 10-year CVD risk levels across regions (with multiple imputation) 32

Figure S10.Sensitivity analysis of the consistency of three CVD risk assessment models in classifying low, moderate, and high risk levels across regions (with multiple imputation) 33

Figure S11.Sensitivity analysis of the distribution of 10-year CVD risk levels across regions (without regional recalibration) 34

Figure S12.Sensitivity analysis of the consistency of three CVD risk assessment models in classifying low, moderate, and high risk levels across regions (without regional recalibration) .35

References......36

Text S1. The search method for eligible surveys in countries where we were unable to acquire a WHO-STEPS survey

Search engine: Google

Search terms: “[country name]” AND (“population-based”)

Inclusion criteria for a survey:
1. Population-based and nationally representative.
2. Includes age groups between 40 and 69 years.
3. Contains key variables required for the study: gender, age, systolic blood pressure, total cholesterol, high-density lipoprotein, smoking history, diabetes history, hypertension treatment, and history of cardiovascular disease.

Countries included in search:
Albania, America, Andorra, Angola, Antigua and Barbuda, Argentina, Australia, Austria, Belgium, Belize, Bolivia, Bosnia and Herzegovina, Brazil, Bulgaria, Burundi, Canada, Channel Islands, Chile, China, Costa Rica, Croatia, Cuba, Curaçao, Cyprus, Czech Republic, Denmark, Djibouti, Dominican Republic, El Salvador, Equatorial Guinea, Estonia, Faroe Islands, Finland, France, Germany, Gibraltar, Greece, Greenland, Guam, Guinea-Bissau, Haiti, Honduras, Hong Kong SAR, China, Hungary, Iceland, Ireland, Isle of Man, Italy, Jamaica, Japan, Kazakhstan, Korea, Rep., Kosovo, Latvia, Liechtenstein, Lithuania, Luxembourg, Macao SAR, China, Malta, Mexico, Monaco, Montenegro, Namibia, Netherlands, New Zealand, Nicaragua, North Macedonia, Northern Mariana Islands, Norway, Panama, Peru, Philippines, Poland, Portugal, Puerto Rico, Romania, Russian Federation, San Marino, Serbia, Singapore, Sint Maarten (Dutch part), Slovak Republic, Slovenia, Somalia, South Africa, South Sudan, Spain, St. Martin (French part), Sweden, Switzerland, Taiwan, China, Thailand, Tunisia, Turks and Caicos Islands, United Kingdom, Virgin Islands (U.S.), Yemen, Rep.

Date of search: July 2024

Text S2. Harmonization and Standardization of Variables Across National Surveys

Key predictor variables were extracted and harmonized based on the requirements of cardiovascular risk models. All variables were recoded and standardized prior to model input.

Systolic blood pressure: Retained as a continuous variable and standardized to millimeters of mercury (mmHg). When multiple readings were available, the mean of the last two was used, in accordance with WHO STEPS protocols. If only a single reading was reported, it was retained without modification.

Total cholesterol and HDL cholesterol: Retained as continuous variables and standardized to milligrams per deciliter (mg/dl), using a conversion factor of 38.67 for values originally reported in mmol/L. Biochemical assay data were prioritized; self-reported hyperlipidemia was not used as a substitute.

Smoking status: Harmonized into a binary indicator (current smoker vs. non-smoker).

Diabetes: Defined based on the availability of clinical or self-reported data. Where available, the following criteria were considered evidence of diabetes: fasting plasma glucose $\geq$ 7.0 mmol/L, HbA1c $\geq$ 6.5%, or the use of insulin or oral hypoglycemic agents. In the absence of clinical measures, self-reported physician diagnosis was used..

Missing data and exclusions: Participants with physiologically implausible values or missing values in core continuous variables were excluded. For binary variables (e.g., smoking, diabetes, antihypertensive treatment), missing values were imputed as "no" to preserve sample size and reflect common assumptions in population-based surveys.

All analyses were conducted using Stata 18.

TableS1. Population characteristics included in the databases for analysis

Cohort Study	Population Characteristics	Location	Analytical sample
WHO Stepwise Survey[1]	Adult populations across a broad age range; covers major behavioral and biological risk factors for NCDs	46 countries globally	67,065
English Longitudinal Study of Ageing (ELSA)[2]	Adults aged 50 and above; focuses on health, social, and economic well-being in older age	United Kingdom	3,929
Health and Aging in Africa: Longitudinal Study of an INDEPTH Community in South Africa (HAALASA)[3]	Rural elderly population; collects data on chronic diseases, cognitive function, and lifestyle factors	South Africa	2824
Mexican Health and Aging Study (MHAS)[4]	Adults aged 50 and above; focuses on health, socioeconomic status, and migration patterns	Mexico	542
Costa Rican Longevity and Healthy Aging Study (CRELES)[5]	Adults born in 1945 or earlier; explores factors related to longevity and health in aging	Costa Rica	513
Midlife in Japan (MIDJA)[6]	Adults aged 30–79; studies psychosocial, physical, and physiological aspects of health	Japan	213
China Health and Retirement Longitudinal Study (CHARLS)[7]	Adults aged 45 and above; examines health, socioeconomics, and aging-related factors	China	9,077
National Health and Nutrition Examination Survey (NHANES)[8]	All ages; provides comprehensive health and nutritional status data across U.S. population	America	2,267

TableS2. Characteristics of cardiovascular risk assessment models used in the study

		WHO	SCORE2		Framingham
Age group for which applicable (years)		40–80 years	40–69 years		30–75 years
Study location		Europe, North America, Japan, and Australia	Europe		USA
	Age	√	√		√
	Gender	√	√		√
	SBP (mmHg)	√	√		√
	TC (mg/dl)	√	√		√
	HDL (mg/dl)		√		√
	Smoking history	√	√		√
	History of diabetes	√	√		√
	hypertension treatment		√		√
	Adjusted by region	√			
risk stratification			< 50 years	50-69years	
	Low risk	< 10%	< 2.5%	< 5%	< 10%
	moderate risk	10% to < 20%	2.5% to < 7.5%	5% to < 10%	10% to < 20%
	High risk	⩾20%	⩾7.5%	⩾10%	⩾20%
Cardiovascular outcomes		I21-I25, I 60-I90	Included: I10-I16, I20-I25, I46-I52, I60-I69, I70-I73, R96.0-R96.1 excluded: I51.4, I60, I62, I67.1, I67.5 ,I68.2		CHD (coronary death, myocardial infarction, coronary insufficiency, and angina), cerebrovascular events (including ischemic stroke, hemorrhagic stoke, and transient ischemic attack), peripheral artery disease (intermittent claudication), and heart failure

In defining cardiovascular disease outcomes, the WHO and SCORE2 models use ICD-10 codes, whereas the Framingham model specifies disease names.

TableS3. GRABDROP compliance checklist for the present study

JoGH guideline item	Purpose	Author response for this study
1. List all papers published by each co-author in the previous three years based on secondary analyses of big data repositories.	Prevents potential involvement of “paper mills” or guest authorship without genuine contribution.	1. Zhang J, Xu H, Liu Y, et al. Time in target range of fasting blood glucose ranges defined by WHO and ADA guidelines and cardiorenal Risk: Insights from two cohorts. Diabetes Res Clin Pract. 2025;226:112323. doi:10.1016/j.diabres.2025.112323<br>2. Zhang, Z., Nie, Z., Chen, K. et al. Association between intensive blood pressure lowering and stroke-free survival among patients with and without Diabetes. Sci Rep 14, 21551 (2024). https://doi.org/10.1038/s41598-024-72211-7<br>3. Lv Z, Ji Y, Li C, et al. Self-reported sleep duration and quality and cardiovascular diseases among middle-aged and older Chinese: A 7-year longitudinal cohort study. J Clin Hypertens (Greenwich). 2024;26(10):1145-1154. doi:10.1111/jch.14883<br>4. Li C, Yang C, Shao F, et al. Generalisability and potential deaths averted from intensive blood pressure treatment among the elderly population in the US and China: A nationally representative cross-sectional study. J Glob Health. 2023;13:04100. Published 2023 Sep 8. doi:10.7189/jogh.13.04100<br>5. Huang, R., Kong, X., Geng, R. et al. Joint and interactive associations of body mass index and genetic factors with cardiovascular disease: a prospective study in UK Biobank. BMC Public Health 24, 2371 (2024). doi:10.1186/s12889-024-19916-6<br>6. Hao J, Liu Z, Hu B, et al. Long-term exposure to outdoor fine particulate and physical activity with mortality and cardiovascular events: an analysis of the Prospective Urban Rural Epidemiology (PURE)-China cohort study. Lancet Reg Health West Pac. 2025;59:101584. Published 2025 May 29. doi:10.1016/j.lanwpc.2025.101584
2. Explain the key elements of your study design and dataset use that make this study original.	Prevents duplicate publication where existing published information is merely reorganised.	Our study is the first to systematically compare three major cardiovascular risk prediction models (WHO, SCORE2, and Framingham) across 53 nationally representative surveys spanning seven global regions. Previous research has primarily consisted of single-country analyses, with only a small number of studies examining risk prediction across multiple countries. None has conducted a cross-model, cross-country comparison at a global scale using harmonised STEPS-equivalent datasets. The present analysis therefore provides novel insights into between-model discrepancies and regional variations in risk distribution that have not been evaluated previously.
3. List all publications addressing similar research questions in the same dataset and indicate where they are cited.	Prevents duplication where other groups have already studied the same question using the same dataset.	Most previous studies assessing CVD risk models have been limited to single-country analyses, with only a few involving small multi-country comparisons. These works, cited in our Discussion (Refs 13, 22, 23), have not evaluated model performance at a global scale. No prior study has systematically compared multiple major CVD risk models across world regions. Our analysis therefore addresses this gap by providing the first harmonised, multi-country assessment relevant to global and regional prevention efforts.
4. Explain how multiple testing was addressed (statistical threshold) and where this is described in Methods.	Prevents false-positive findings from multiple comparisons in big datasets.	No hypothesis testing was performed; multiple-testing correction was not applicable.
5. Declare the extent of AI chatbot use and specify which parts of the manuscript they contributed to.	Ensures transparency and avoids inappropriate AI-generated content.	No AI tools were used in this study.

TableS4. Baseline characteristics of participants stratified by country

Region	Country	Year	Sample size	Age （years）	Proportion of men （%）	SBP （mmHg）	TC (mg/dl)	HDL (mg/dl)	Proportion （%） of smokers	Proportion （%） of people with diabetes	Proportion (%) of people with treatment for hypertension	WHO CVD risk score （%）	SCORE2 CVD risk score （%）	Framingham CVD risk score （%）	Proportions (%) of moderate risk and high-risk in the WHO CVD model	Proportions (%) of moderate risk and high-risk in the SCORE2 CVD model	Proportions (%) of moderate risk and high-risk in the Framingham CVD model
East Asia & Pacific	Brunei Darussalam	2015	962	53.23 (52.72, 53.74)	45.84 (42.69, 48.99)	133.21 (132.03, 134.39)	199.78 (197.11, 202.45)	48.87 (48.02, 49.72)	12.27 (10.19, 14.34)	22.56 (19.92, 25.20)	35.76 (32.73, 38.79)	6.02 (5.74, 6.29)	3.88 (3.65, 4.12)	14.35 (13.59, 15.12)	16.63 (14.28, 18.99)	34.41 (31.41, 37.41)	51.87 (48.71, 55.03)
East Asia & Pacific	China	2015	9,077	56.11 (55.96, 56.26)	46.22 (45.19, 47.24)	125.67 (125.29, 126.06)	183.30 (182.56, 184.04)	51.34 (51.11, 51.57)	28.54 (27.62, 29.47)	12.40 (11.73, 13.08)	18.05 (17.25, 18.84)	8.65 (8.52, 8.77)	3.88 (3.80, 3.96)	12.83 (12.59, 13.07)	32.43 (31.47, 33.40)	29.88 (28.94, 30.82)	45.95 (44.93, 46.98)
East Asia & Pacific	Fiji	2002	1,423	50.17 (49.82, 50.52)	39.49 (36.95, 42.03)	131.93 (130.77, 133.09)	208.66 (205.83, 211.49)	45.65 (43.78, 47.53)	35.28 (32.79, 37.76)	18.55 (16.53, 20.57)	14.41 (12.58, 16.23)	6.83 (6.56, 7.11)	4.14 (3.88, 4.40)	14.84 (14.12, 15.57)	21.29 (19.17, 23.42)	37.67 (35.15, 40.18)	50.32 (47.72, 52.91)
East Asia & Pacific	Japan	2012	213	55.54 (54.38, 56.71)	46.01 (39.32, 52.70)	127.09 (124.70, 129.47)	211.77 (207.02, 216.51)	67.85 (65.39, 70.30)	15.02 (10.23, 19.82)	17.37 (12.28, 22.46)	16.43 (11.46, 21.41)	6.65 (5.92, 7.37)	3.72 (3.11, 4.34)	12.40 (10.57, 14.23)	20.19 (14.80, 25.58)	25.82 (19.94, 31.70)	40.85 (34.24, 47.45)
East Asia & Pacific	Kiribati	2015	474	51.52 (50.80, 52.24)	46.84 (42.34, 51.33)	133.93 (132.04, 135.83)	162.38 (157.12, 167.63)	34.33 (30.95, 37.72)	49.16 (44.66, 53.66)	28.90 (24.82, 32.98)	6.96 (4.67, 9.25)	7.56 (7.16, 7.97)	5.42 (5.00, 5.83)	18.16 (16.86, 19.45)	23.63 (19.80, 27.45)	55.06 (50.59, 59.54)	63.71 (59.38, 68.04)
East Asia & Pacific	Lao People's Democratic Republic	2013	1,178	49.89 (49.51, 50.27)	41.85 (39.03, 44.67)	123.94 (122.74, 125.13)	173.61 (171.07, 176.16)	39.51 (38.67, 40.34)	33.28 (30.59, 35.97)	8.66 (7.05, 10.26)	6.28 (4.90, 7.67)	4.10 (3.89, 4.31)	2.79 (2.61, 2.97)	10.37 (9.78, 10.96)	4.84 (3.61, 6.06)	22.50 (20.11, 24.88)	35.99 (33.25, 38.73)
East Asia & Pacific	Marshall Islands	2002	376	49.08 (48.44, 49.72)	42.29 (37.29, 47.28)	123.54 (121.40, 125.68)	199.06 (193.11, 205.01)	38.27 (37.02, 39.53)	14.89 (11.30, 18.49)	51.33 (46.28, 56.38)	11.97 (8.69, 15.25)	5.96 (5.56, 6.35)	3.66 (3.32, 4.00)	13.27 (12.08, 14.45)	14.63 (11.06, 18.20)	38.03 (33.12, 42.94)	49.20 (44.15, 54.26)
East Asia & Pacific	Micronesia	2002	425	49.49 (48.88, 50.11)	41.18 (36.50, 45.86)	129.23 (127.32, 131.15)	209.96 (205.54, 214.38)	57.83 (50.32, 65.34)	30.59 (26.21, 34.97)	22.59 (18.61, 26.56)	6.59 (4.23, 8.95)	6.18 (5.79, 6.56)	3.38 (3.07, 3.70)	12.25 (11.26, 13.25)	13.88 (10.60, 17.17)	31.76 (27.34, 36.19)	48.47 (43.72, 53.22)
East Asia & Pacific	Mongolia	2019	2,694	52.20 (51.90, 52.51)	44.02 (42.15, 45.90)	129.40 (128.65, 130.16)	182.16 (180.65, 183.68)	54.10 (53.61, 54.60)	25.98 (24.33, 27.64)	11.84 (10.62, 13.06)	25.98 (24.33, 27.64)	7.69 (7.35, 8.03)	3.09 (2.95, 3.23)	10.90 (10.49, 11.31)	24.35 (22.73, 25.97)	23.68 (22.08, 25.29)	36.60 (34.78, 38.42)
East Asia & Pacific	Myanmar	2014	4,653	51.11 (50.90, 51.31)	35.63 (34.26, 37.01)	131.91 (131.24, 132.59)	187.87 (186.72, 189.02)	46.71 (46.37, 47.04)	25.83 (24.58, 27.09)	9.71 (8.86, 10.57)	12.27 (11.33, 13.21)	4.93 (4.81, 5.05)	3.01 (2.91, 3.11)	10.89 (10.61, 11.18)	9.63 (8.78, 10.48)	23.36 (22.15, 24.58)	38.45 (37.05, 39.85)
East Asia & Pacific	Nauru	2015	274	50.44 (49.52, 51.35)	46.35 (40.45, 52.25)	133.59 (131.16, 136.02)	162.64 (156.25, 169.03)	33.02 (31.22, 34.83)	38.32 (32.56, 44.08)	31.39 (25.89, 36.88)	10.22 (6.63, 13.81)	2.49 (2.10, 2.87)	4.72 (4.17, 5.26)	15.05 (13.51, 16.60)	2.19 (0.46, 3.92)	45.26 (39.36, 51.15)	54.74 (48.85, 60.64)
East Asia & Pacific	New Zealand	2014	234	51.32 (50.44, 52.20)	46.58 (40.19, 52.97)	135.02 (132.66, 137.38)	212.60 (206.88, 218.32)	41.13 (39.15, 43.11)	50.85 (44.45, 57.26)	47.86 (41.46, 54.26)	17.09 (12.27, 21.92)	9.39 (8.57, 10.22)	6.78 (6.07, 7.49)	21.43 (19.47, 23.38)	43.16 (36.82, 49.51)	62.82 (56.63, 69.01)	73.50 (67.85, 79.16)
East Asia & Pacific	Palau	2016	798	52.33 (51.79, 52.87)	50.00 (46.53, 53.47)	130.31 (128.91, 131.71)	79.94 (76.72, 83.15)	29.37 (28.27, 30.47)	17.04 (14.43, 19.65)	20.68 (17.87, 23.49)	17.29 (14.67, 19.92)	1.58 (1.47, 1.68)	3.28 (3.03, 3.53)	9.35 (8.44, 10.26)	0.50 (0.01, 0.99)	26.32 (23.26, 29.37)	28.45 (25.32, 31.58)
East Asia & Pacific	Tokelau	2014	234	51.32 (50.44, 52.20)	46.58 (40.19, 52.97)	135.02 (132.66, 137.38)	212.60 (206.88, 218.32)	41.13 (39.15, 43.11)	50.85 (44.45, 57.26)	47.86 (41.46, 54.26)	17.09 (12.27, 21.92)	2.56 (2.23, 2.89)	6.78 (6.07, 7.49)	21.43 (19.47, 23.38)	2.56 (0.54, 4.59)	62.82 (56.63, 69.01)	73.50 (67.85, 79.16)
East Asia & Pacific	Tonga	2004	478	49.88 (49.26, 50.49)	41.00 (36.59, 45.41)	130.47 (128.82, 132.11)	201.31 (198.01, 204.61)	43.82 (40.94, 46.70)	24.69 (20.82, 28.55)	14.23 (11.09, 17.36)	5.65 (3.58, 7.72)	5.77 (5.38, 6.15)	3.28 (2.94, 3.61)	12.43 (11.37, 13.49)	12.97 (9.96, 15.98)	31.38 (27.22, 35.54)	43.72 (39.28, 48.17)
East Asia & Pacific	Tuvalu	2015	500	53.37 (52.70, 54.04)	44.40 (40.04, 48.76)	147.61 (145.46, 149.75)	171.82 (167.63, 176.02)	26.72 (25.20, 28.25)	34.80 (30.62, 38.98)	19.40 (15.93, 22.87)	9.00 (6.49, 11.51)	3.25 (2.93, 3.56)	6.73 (6.20, 7.26)	23.74 (22.23, 25.26)	5.40 (3.42, 7.38)	59.20 (54.89, 63.51)	79.00 (75.43, 82.57)
East Asia & Pacific	Viet Nam	2021	2,181	53.73 (53.39, 54.07)	51.12 (49.03, 53.22)	135.25 (134.34, 136.16)	202.55 (200.44, 204.65)	46.41 (45.59, 47.24)	26.78 (24.92, 28.64)	13.66 (12.22, 15.10)	13.98 (12.53, 15.44)	6.91 (6.66, 7.16)	4.74 (4.53, 4.95)	16.88 (16.24, 17.53)	21.50 (19.78, 23.23)	40.58 (38.52, 42.64)	56.35 (54.27, 58.43)
Europe & Central Asia	Armenia	2016	841	54.05 (53.50, 54.59)	25.33 (22.39, 28.27)	150.60 (147.07, 154.12)	184.67 (181.56, 187.77)	44.75 (43.85, 45.65)	16.05 (13.57, 18.53)	10.11 (8.07, 12.14)	14.51 (12.13, 16.89)	12.36 (11.06, 13.65)	6.66 (5.68, 7.64)	15.71 (14.61, 16.82)	32.34 (29.18, 35.50)	34.96 (31.74, 38.18)	50.77 (47.39, 54.15)
Europe & Central Asia	Azerbaijan	2017	1,475	53.62 (53.23, 54.02)	39.73 (37.23, 42.23)	139.53 (137.72, 141.35)	184.42 (182.06, 186.78)	43.21 (42.57, 43.86)	19.53 (17.50, 21.55)	12.68 (10.98, 14.38)	19.66 (17.63, 21.69)	10.26 (9.52, 11.00)	5.03 (4.54, 5.52)	15.57 (14.76, 16.37)	29.22 (26.90, 31.54)	33.08 (30.68, 35.49)	51.32 (48.77, 53.87)
Europe & Central Asia	Belarus	2016	2,963	53.47 (53.17, 53.76)	39.99 (38.23, 41.76)	143.39 (142.56, 144.22)	196.67 (195.21, 198.13)	53.64 (53.07, 54.21)	25.72 (24.14, 27.29)	6.34 (5.47, 7.22)	31.32 (29.65, 32.99)	11.58 (11.24, 11.91)	4.06 (3.90, 4.22)	14.88 (14.40, 15.36)	45.80 (44.00, 47.59)	33.04 (31.35, 34.73)	53.36 (51.56, 55.15)
Europe & Central Asia	Georgia	2016	1,674	54.96	26.94	137.99 (136.76,	184.41	40.13 (39.43,	18.64 (16.77,	9.56 (8.15, 10.97)	26.16 (24.06,	9.44 (8.93,	4.58 (4.31, 4.85)	15.73 (15.00,	31.78 (29.55,	35.01 (32.72,	53.11 (50.72,

				(54.55, 55.36)	(24.82, 29.07)	139.23)	(182.17, 186.66)	40.82)	20.50)		28.27)	9.95)		16.46)	34.01)	37.29)	55.50)
Europe & Central Asia	Republic of Moldova	2013	1,725	54.14 (53.76, 54.52)	39.36 (37.06, 41.67)	147.58 (145.82, 149.34)	186.43 (184.57, 188.29)	56.12 (55.17, 57.07)	19.07 (17.22, 20.93)	9.86 (8.45, 11.26)	18.03 (16.21, 19.84)	12.81 (12.18, 13.43)	4.76 (4.35, 5.17)	14.14 (13.51, 14.78)	48.46 (46.11, 50.82)	31.30 (29.12, 33.49)	49.33 (46.97, 51.69)
Europe & Central Asia	Tajikistan	2016	1,187	50.90 (50.45, 51.34)	43.39 (40.57, 46.21)	143.81 (142.51, 145.11)	166.24 (163.94, 168.54)	44.14 (43.37, 44.91)	5.31 (4.03, 6.58)	11.04 (9.25, 12.82)	15.00 (12.96, 17.03)	7.04 (6.55, 7.53)	3.19 (2.98, 3.39)	11.34 (10.74, 11.95)	21.23 (18.90, 23.56)	24.52 (22.07, 26.96)	40.19 (37.40, 42.97)
Europe & Central Asia	Turkmenistan	2018	1,680	50.70 (50.33, 51.07)	42.56 (40.20, 44.92)	135.83 (134.91, 136.76)	172.75 (170.53, 174.98)	53.03 (52.27, 53.79)	4.58 (3.58, 5.58)	8.15 (6.85, 9.46)	14.40 (12.73, 16.08)	5.98 (5.63, 6.34)	2.40 (2.26, 2.54)	8.83 (8.40, 9.26)	16.55 (14.77, 18.32)	15.83 (14.09, 17.58)	27.56 (25.42, 29.70)
Europe & Central Asia	Ukraine	2019	1,540	55.09 (54.65, 55.53)	33.18 (30.83, 35.53)	137.42 (136.10, 138.74)	189.64 (187.52, 191.76)	48.39 (47.65, 49.13)	25.26 (23.09, 27.43)	13.31 (11.62, 15.01)	25.19 (23.03, 27.36)	12.15 (11.60, 12.69)	4.75 (4.42, 5.08)	15.18 (14.45, 15.92)	47.14 (44.65, 49.64)	37.01 (34.60, 39.42)	50.58 (48.09, 53.08)
Europe & Central Asia	United Kingdom	2008	3,929	59.97 (59.81, 60.13)	44.82 (43.27, 46.38)	130.28 (129.77, 130.79)	223.02 (221.65, 224.39)	60.90 (60.40, 61.41)	13.87 (12.79, 14.95)	4.94 (4.26, 5.62)	20.97 (19.70, 22.25)	7.56 (7.41, 7.71)	4.04 (3.95, 4.13)	13.66 (13.38, 13.94)	25.20 (23.84, 26.55)	28.53 (27.12, 29.94)	57.60 (56.05, 59.14)
Latin America & Caribbean	Bahamas	2019	725	52.32 (51.76, 52.88)	36.69 (33.18, 40.20)	134.21 (132.76, 135.66)	160.56 (156.84, 164.29)	76.70 (76.35, 77.04)	11.17 (8.88, 13.47)	14.76 (12.18, 17.34)	25.79 (22.61, 28.98)	4.93 (4.62, 5.24)	1.95 (1.76, 2.14)	6.95 (6.35, 7.54)	9.24 (7.13, 11.35)	9.38 (7.26, 11.50)	18.48 (15.66, 21.31)
Latin America & Caribbean	Costa Rica	2007	513	65.83 (65.64, 66.01)	44.83 (40.53, 49.14)	142.31 (140.46, 144.16)	207.04 (203.56, 210.52)	42.96 (41.87, 44.04)	11.31 (8.57, 14.05)	20.86 (17.34, 24.37)	38.21 (34.00, 42.41)	9.85 (9.50, 10.19)	8.60 (8.21, 8.99)	27.33 (25.96, 28.70)	39.38 (35.15, 43.60)	78.17 (74.59, 81.74)	91.03 (88.56, 93.51)
Latin America & Caribbean	Guyana	2016	404	52.78 (51.96, 53.59)	36.88 (32.18, 41.59)	135.25 (132.86, 137.64)	210.74 (205.88, 215.60)	52.91 (50.83, 55.00)	10.64 (7.64, 13.65)	23.27 (19.15, 27.39)	22.52 (18.45, 26.60)	6.29 (5.71, 6.86)	3.93 (3.39, 4.47)	14.08 (12.90, 15.26)	15.84 (12.28, 19.40)	34.16 (29.53, 38.78)	51.49 (46.61, 56.36)
Latin America & Caribbean	Mexico	2012	542	63.05 (62.63, 63.47)	34.69 (30.68, 38.69)	140.96 (139.21, 142.72)	207.20 (203.00, 211.39)	40.77 (39.88, 41.66)	11.07 (8.43, 13.71)	44.83 (40.65, 49.02)	38.56 (34.46, 42.66)	9.84 (9.38, 10.30)	8.71 (8.23, 9.19)	27.62 (26.12, 29.12)	41.33 (37.18, 45.47)	72.69 (68.94, 76.44)	86.16 (83.26, 89.07)
Latin America & Caribbean	Uruguay	2006	458	51.97 (51.30, 52.64)	28.82 (24.67, 32.97)	134.30 (132.19, 136.41)	191.98 (187.89, 196.06)	52.10 (50.42, 53.79)	30.57 (26.35, 34.79)	7.86 (5.40, 10.32)	23.80 (19.90, 27.70)	5.13 (4.64, 5.63)	3.27 (2.89, 3.64)	11.43 (10.43, 12.43)	11.57 (8.64, 14.50)	21.18 (17.44, 24.92)	43.45 (38.91, 47.99)
Middle East & North Africa	Algeria	2016	3,034	51.50 (51.21, 51.80)	45.52 (43.75, 47.29)	136.15 (135.30, 136.99)	175.11 (173.63, 176.59)	46.24 (45.76, 46.73)	11.27 (10.15, 12.40)	17.93 (16.57, 19.30)	13.97 (12.74, 15.21)	10.00 (9.70, 10.30)	3.53 (3.35, 3.72)	11.61 (11.21, 12.01)	36.78 (35.07, 38.50)	27.22 (25.64, 28.81)	39.88 (38.14, 41.62)
Middle East & North Africa	Iraq	2015	1,564	50.99 (50.58, 51.39)	39.64 (37.22, 42.07)	138.53 (137.58, 139.48)	193.43 (190.81, 196.04)	44.84 (44.00, 45.69)	16.75 (14.90, 18.60)	26.09 (23.91, 28.26)	23.34 (21.24, 25.43)	11.24 (10.83, 11.65)	4.13 (3.91, 4.35)	14.74 (14.06, 15.42)	43.73 (41.28, 46.19)	37.02 (34.63, 39.41)	51.92 (49.44, 54.39)
Middle East & North Africa	Jordan	2019	1,548	52.12 (51.70, 52.55)	36.56 (34.16, 38.96)	126.15 (125.21, 127.09)	160.46 (158.38, 162.55)	40.10 (39.57, 40.63)	28.42 (26.18, 30.67)	22.80 (20.71, 24.89)	25.58 (23.41, 27.75)	9.86 (9.49, 10.22)	3.70 (3.50, 3.90)	11.93 (11.34, 12.53)	37.27 (34.87, 39.68)	31.46 (29.15, 33.77)	40.89 (38.44, 43.34)
Middle East & North Africa	Kuwait	2014	804	49.98 (49.46, 50.49)	35.82 (32.51, 39.14)	148.35 (143.29, 153.42)	202.65 (199.85, 205.45)	49.03 (48.24, 49.82)	13.68 (11.31, 16.06)	28.86 (25.72, 31.99)	29.35 (26.21, 32.50)	16.16 (14.53, 17.80)	9.41 (7.89, 10.92)	16.29 (14.89, 17.68)	40.55 (37.15, 43.94)	33.08 (29.83, 36.34)	47.01 (43.56, 50.46)
Middle East & North Africa	Lebanon	2017	1,306	52.20 (51.78, 52.63)	39.82 (37.16, 42.47)	133.79 (132.66, 134.93)	213.98 (211.56, 216.40)	47.64 (46.99, 48.29)	33.46 (30.90, 36.02)	18.45 (16.35, 20.56)	19.22 (17.08, 21.36)	12.56 (12.06, 13.05)	4.61 (4.32, 4.90)	15.66 (14.87, 16.44)	50.92 (48.21, 53.63)	37.29 (34.67, 39.91)	51.99 (49.28, 54.70)
Middle East & North Africa	Morocco	2017	2,406	52.60 (52.27, 52.93)	36.99 (35.06, 38.92)	136.53 (135.71, 137.35)	150.10 (148.47, 151.73)	45.00 (44.36, 45.65)	8.40 (7.29, 9.50)	19.62 (18.03, 21.20)	10.18 (8.97, 11.39)	9.71 (9.42, 10.00)	3.33 (3.18, 3.48)	10.65 (10.22, 11.08)	36.99 (35.06, 38.92)	25.19 (23.45, 26.92)	36.41 (34.49, 38.33)
Middle East & North Africa	Occupied Palestinian Territory*	2010	3,111	50.54 (50.29, 50.79)	34.68 (33.01, 36.36)	127.78 (127.13, 128.43)	197.40 (195.87, 198.94)	46.65 (46.03, 47.28)	17.94 (16.59, 19.28)	27.23 (25.66, 28.79)	27.58 (26.01, 29.15)	9.75 (9.50, 10.00)	3.36 (3.22, 3.50)	12.85 (12.39, 13.31)	37.93 (36.22, 39.63)	28.00 (26.42, 29.58)	43.33 (41.59, 45.07)
Middle East & North Africa	Qatar	2012	724	49.14 (48.63, 49.65)	39.92 (36.35, 43.48)	129.02 (126.76, 131.27)	169.51 (166.68, 172.35)	51.21 (49.83, 52.59)	13.81 (11.30, 16.33)	29.83 (26.50, 33.17)	26.24 (23.04, 29.45)	9.02 (8.29, 9.75)	3.34 (2.75, 3.93)	10.48 (9.53, 11.42)	27.21 (23.97, 30.45)	22.51 (19.47, 25.56)	32.87 (29.45, 36.29)
North America	America	2017	2,267	55.01 (54.66, 55.35)	47.46 (45.41, 49.52)	128.92 (128.13, 129.72)	196.89 (195.22, 198.55)	53.45 (52.79, 54.12)	18.66 (17.06, 20.26)	18.48 (16.88, 20.08)	32.25 (30.32, 34.17)	8.52 (8.27, 8.76)	3.86 (3.71, 4.01)	13.44 (12.97, 13.91)	33.04 (31.10, 34.98)	31.36 (29.45, 33.27)	50.29 (48.23, 52.34)
South Asia	Bangladesh	2018	2,788	50.24 (49.96, 50.53)	52.15 (50.30, 54.01)	127.58 (126.78, 128.38)	179.27 (177.82, 180.72)	83.46 (82.68, 84.23)	27.30 (25.64, 28.95)	14.67 (13.36, 15.98)	11.48 (10.29, 12.66)	5.11 (4.96, 5.26)	1.79 (1.70, 1.87)	6.87 (6.62, 7.12)	10.44 (9.30, 11.57)	10.08 (8.96, 11.20)	20.30 (18.81, 21.79)
South Asia	Nepal	2012	1,977	51.79 (51.41, 52.17)	36.62 (34.50, 38.74)	136.11 (135.15, 137.08)	174.37 (172.45, 176.28)	40.83 (40.28, 41.38)	24.58 (22.68, 26.48)	8.40 (7.17, 9.62)	8.45 (7.22, 9.67)	5.89 (5.68, 6.10)	3.68 (3.50, 3.85)	12.25 (11.73, 12.76)	15.58 (13.98, 17.18)	29.74 (27.73, 31.76)	42.24 (40.06, 44.41)
Sub-Saharan Africa	Botswana	2014	1,214	51.84 (51.40, 52.29)	30.15 (27.57, 32.73)	139.25 (137.89, 140.61)	161.10 (158.49, 163.71)	54.59 (53.00, 56.18)	14.91 (12.91, 16.91)	8.07 (6.54, 9.60)	25.37 (22.92, 27.82)	5.08 (4.84, 5.32)	2.74 (2.54, 2.93)	9.44 (8.89, 9.99)	10.05 (8.36, 11.74)	18.45 (16.27, 20.63)	30.40 (27.81, 32.98)
Sub-Saharan Africa	Cabo Verde	2020	1,193	53.01 (52.54,	39.23 (36.46,	137.65 (136.42, 138.88)	175.58 (172.99,	98.82 (96.86, 100.79)	7.88 (6.35, 9.41)	10.39 (8.66, 12.13)	20.87 (18.57, 23.18)	5.39 (5.13, 5.65)	1.81 (1.67, 1.95)	6.82 (6.41, 7.22)	13.83 (11.87, 15.79)	9.14 (7.50, 10.77)	20.45 (18.16, 22.74)

				53.48)	42.00)		178.18)										
Sub-Saharan Africa	Eswatini	2014	998	52.72 (52.19, 53.25)	32.36 (29.46, 35.27)	137.87 (136.32, 139.42)	165.22 (162.66, 167.78)	50.23 (49.14, 51.32)	8.12 (6.42, 9.81)	10.72 (8.80, 12.64)	16.93 (14.61, 19.26)	5.29 (5.02, 5.57)	3.06 (2.84, 3.29)	10.21 (9.58, 10.85)	13.03 (10.94, 15.11)	22.04 (19.47, 24.62)	33.67 (30.74, 36.60)
Sub-Saharan Africa	Ethiopia	2015	2,872	50.29 (49.98, 50.60)	46.52 (44.69, 48.34)	127.86 (127.03, 128.68)	148.43 (147.04, 149.83)	42.36 (41.88, 42.85)	7.73 (6.75, 8.71)	4.81 (4.02, 5.59)	3.17 (2.53, 3.81)	3.62 (3.51, 3.74)	2.22 (2.13, 2.32)	7.40 (7.13, 7.67)	5.12 (4.31, 5.92)	13.16 (11.93, 14.40)	21.83 (20.32, 23.34)
Sub-Saharan Africa	Kenya	2015	1,499	51.66 (51.23, 52.10)	41.63 (39.13, 44.12)	135.11 (133.90, 136.32)	152.33 (150.20, 154.47)	47.49 (46.63, 48.35)	10.21 (8.67, 11.74)	5.54 (4.38, 6.69)	5.40 (4.26, 6.55)	4.44 (4.25, 4.63)	2.65 (2.50, 2.80)	8.55 (8.13, 8.97)	8.01 (6.63, 9.38)	18.61 (16.64, 20.58)	27.55 (25.29, 29.81)
Sub-Saharan Africa	Lesotho	2012	1,172	52.19 (51.77, 52.61)	31.83 (29.16, 34.49)	138.77 (137.21, 140.32)	145.51 (143.50, 147.52)	49.49 (48.47, 50.52)	14.42 (12.41, 16.43)	5.63 (4.31, 6.95)	13.74 (11.77, 15.71)	4.71 (4.50, 4.92)	2.58 (2.41, 2.74)	8.69 (8.19, 9.18)	8.11 (6.54, 9.67)	14.76 (12.73, 16.79)	27.13 (24.59, 29.68)
Sub-Saharan Africa	Rwanda	2012	2,394	49.99 (49.71, 50.27)	35.96 (34.04, 37.89)	129.01 (128.18, 129.84)	131.43 (130.05, 132.81)	44.40 (43.67, 45.13)	27.44 (25.66, 29.23)	2.38 (1.77, 2.99)	1.04 (0.64, 1.45)	3.61 (3.48, 3.73)	2.08 (1.98, 2.19)	6.48 (6.22, 6.73)	3.84 (3.07, 4.61)	11.49 (10.21, 12.76)	17.13 (15.62, 18.64)
Sub-Saharan Africa	Seychelles	2004	807	51.87 (51.39, 52.36)	45.60 (42.16, 49.04)	137.24 (135.80, 138.68)	218.25 (214.76, 221.74)	53.15 (51.82, 54.48)	18.71 (16.02, 21.40)	16.98 (14.39, 19.57)	29.99 (26.83, 33.15)	6.45 (6.04, 6.86)	3.96 (3.62, 4.31)	15.29 (14.29, 16.30)	16.60 (14.04, 19.17)	31.85 (28.63, 35.06)	52.91 (49.47, 56.36)
Sub-Saharan Africa	South Africa	2015	2,824	55.37 (55.06, 55.67)	44.72 (42.89, 46.56)	136.16 (135.34, 136.98)	162.71 (160.90, 164.52)	59.98 (59.22, 60.74)	10.84 (9.69, 11.98)	96.64 (95.97, 97.30)	23.90 (22.33, 25.48)	9.24 (9.05, 9.43)	5.48 (5.33, 5.64)	15.97 (15.51, 16.43)	37.71 (35.92, 39.50)	50.25 (48.40, 52.09)	60.62 (58.82, 62.43)
Sub-Saharan Africa	Sudan	2016	3,002	51.11 (50.81, 51.41)	42.14 (40.37, 43.90)	137.71 (136.94, 138.48)	162.83 (161.19, 164.48)	34.71 (34.17, 35.25)	6.00 (5.15, 6.85)	14.19 (12.94, 15.44)	10.19 (9.11, 11.28)	8.99 (8.74, 9.23)	3.50 (3.36, 3.64)	12.83 (12.39, 13.27)	32.25 (30.57, 33.92)	27.75 (26.15, 29.35)	44.87 (43.09, 46.65)
Sub-Saharan Africa	Uganda	2014	1,096	50.59 (50.10, 51.08)	41.06 (38.15, 43.97)	133.85 (132.46, 135.25)	144.07 (141.72, 146.41)	45.83 (44.53, 47.14)	14.32 (12.25, 16.40)	2.37 (1.47, 3.27)	4.47 (3.25, 5.69)	3.95 (3.75, 4.14)	2.30 (2.15, 2.46)	7.54 (7.13, 7.95)	6.48 (5.02, 7.94)	14.60 (12.51, 16.69)	23.08 (20.59, 25.58)

Data are mean (95% CI), number of participants (95% CI), unless stated otherwise

* The Occupied Palestinian Territory is not classified by the World Bank; for this analysis, it was grouped with the Middle East & North Africa region.

TableS5. Baseline characteristics of participants stratified by gender

	Male	Female	All Population
Sample size	35,631	50,799	86,430
Age (years)	53.33 (53.24, 53.41)	52.71 (52.64, 52.78)	52.97 (52.91, 53.02)
SBP (mmHg)	133.95 (133.71, 134.19)	133.28 (133.06, 133.50)	133.55 (133.39, 133.72)
TC (mg/dl)	173.89 (173.39, 174.39)	184.14 (183.72, 184.56)	179.91 (179.59, 180.24)
HDL (mg/dl)	47.60 (47.38, 47.81)	51.78 (51.60, 51.96)	50.06 (49.92, 50.20)
Proportion (%) of smokers	36.97 (36.47, 37.47)	7.66 (7.43, 7.89)	19.74 (19.48, 20.01)
Proportion (%) of people with diabetes	16.59 (16.21, 16.98)	16.36 (16.04, 16.68)	16.46 (16.21, 16.70)
Proportion (%) of people with treatment for hypertension	14.41 (14.05, 14.78)	20.10 (19.75, 20.45)	17.76 (17.50, 18.01)
WHO CVD risk score（%）	9.48 (9.39, 9.57)	6.53 (6.47, 6.59)	7.75 (7.70, 7.80)
SCORE2 CVD risk score（%）	5.09 (5.03, 5.15)	2.76 (2.72, 2.80)	3.72 (3.69, 3.75)
Framingham CVD risk score（%）	17.45 (17.30, 17.60)	8.90 (8.81, 8.98)	12.42 (12.34, 12.50)
Proportions (%) of moderate risk and high-risk in the WHO CVD model	34.50 (34.01, 34.99)	18.89 (18.55, 19.23)	25.32 (25.03, 25.61)
Proportions (%) of moderate risk and high-risk in the SCORE2 CVD model	44.94 (44.42, 45.46)	16.80 (16.47, 17.12)	28.40 (28.10, 28.70)
Proportions (%) of moderate risk and high-risk in the Framingham CVD model	63.47 (62.97, 63.97)	28.42 (28.03, 28.81)	42.87 (42.54, 43.20)

Data are mean (95% CI), number of participants (95% CI), unless stated otherwise

TableS6. Global comparison of main and sensitivity analyses

Analysis scenario	WHO CVD risk score（%）	SCORE2 CVD risk score（%）	Framingham CVD risk score（%）	Proportions (%) of moderate risk and high-risk in the WHO CVD model	Proportions (%) of moderate risk and high-risk in the SCORE2 CVD model	Proportions (%) of moderate risk and high-risk in the Framingham CVD model	Proportions (%) of consistent high-risk
Main analysis	7.75 (7.80)	3.72 (5.12)	12.42 (12.28)	25.32	28.40	42.87	21.60
Sensitivity 1: Multiple imputation	7.63 (8.95)	3.76 (5.53)	13.58 (13.15)	23.01	28.91	46.74	14.60
Sensitivity 2: Expanded age	6.66 (8.09)	3.56 (6.06)	10.37 (12.28)	21.47	24.31	33.64	27.71
Sensitivity 3: Without regional recalibration	4.68 (6.78)	3.72 (5.12)	12.42 (12.28)	12.30	28.40	42.87	12.32

Sensitivity analyses included: (i) multiple imputation for missing variables; (ii) expanded age range of 30–80 years ; and (iii) analyses without regional recalibration for WHO and SCORE2.

Data are mean (95% CI) for model-based risk scores, and percentage of particpants for categorical risk groups, unless stated otherwise.

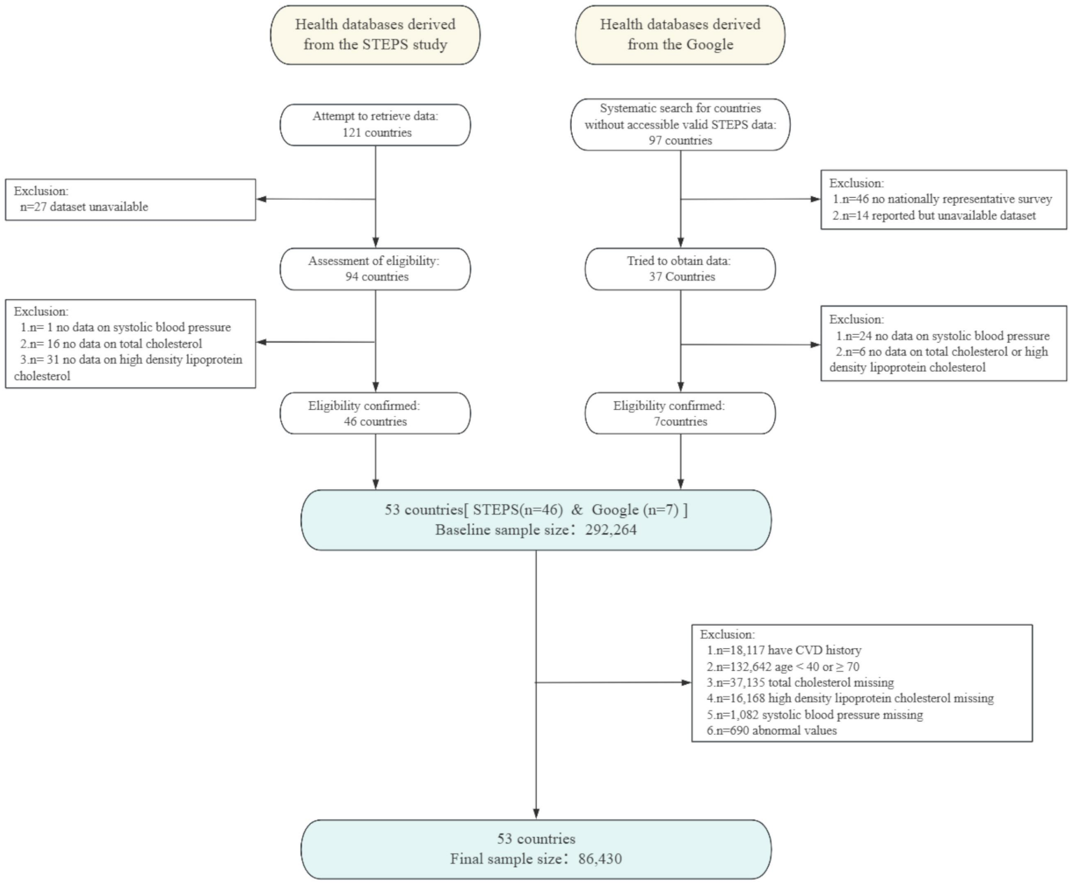

Figure S1. Study flowchart

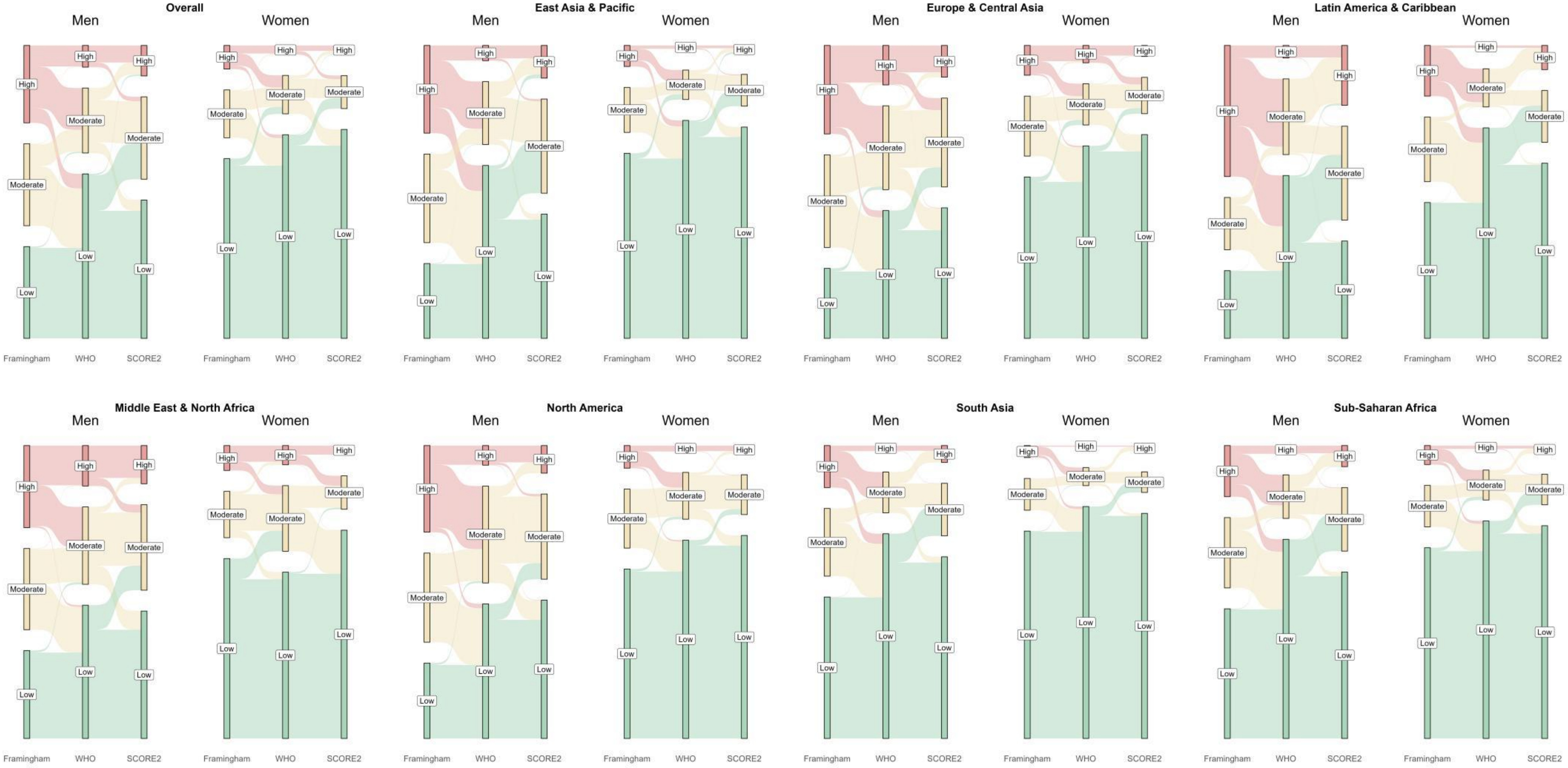

Figure S2. Distribution of 10-year cardiovascular disease risk levels across regions and gender

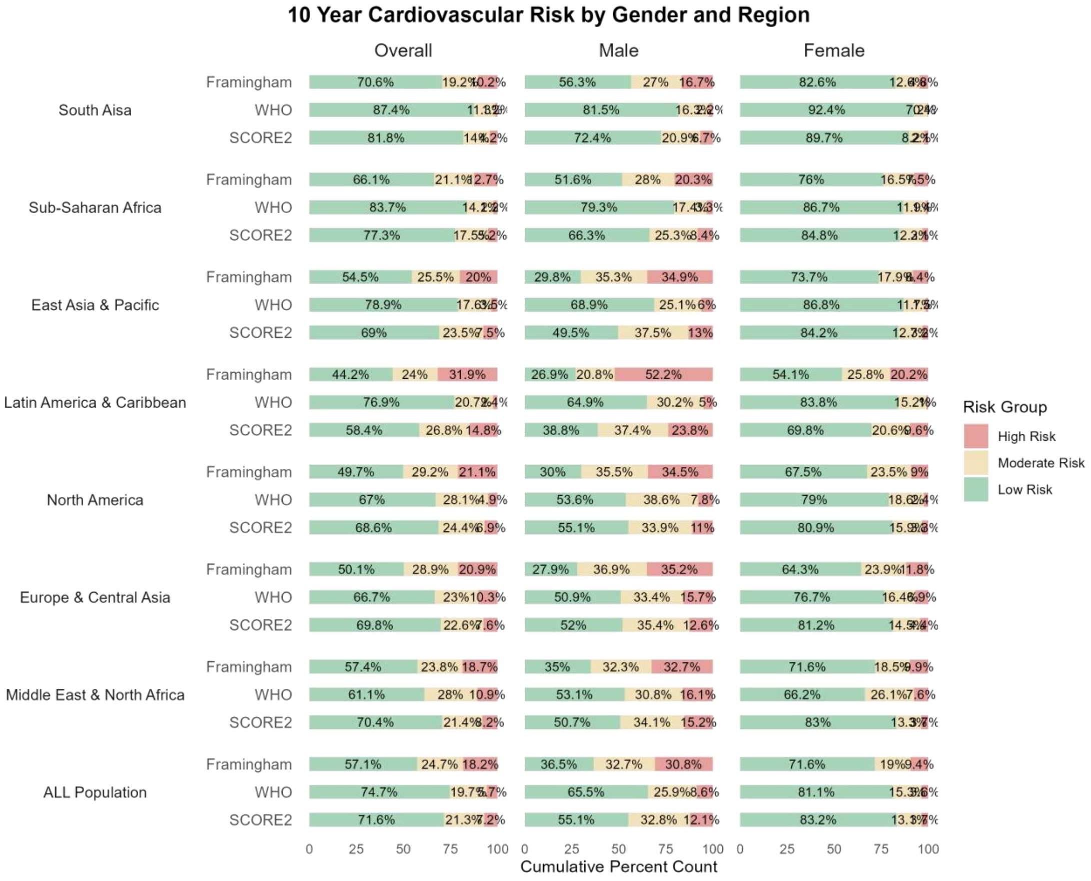

10 Year Cardiovascular Risk by Gender and Region
Overall
Male
Female
South Aisa
Sub-Saharan Africa
East Asia & Pacific
Latin America & Caribbean
North America
Europe & Central Asia
Middle East & North Africa
ALL Population
Framingham
WHO
SCORE2
Risk Group
High Risk
Moderate Risk
Low Risk
Cumulative Percent Count

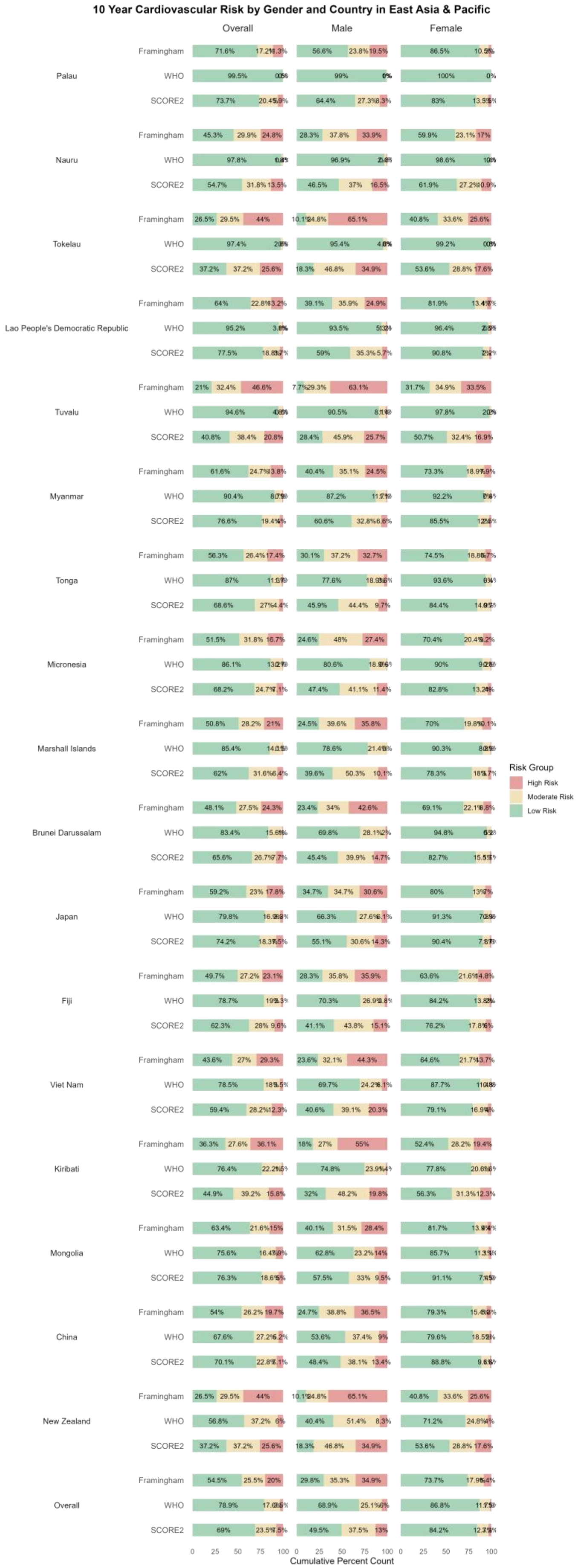
10 Year Cardiovascular Risk by Gender and Country in East Asia & Pacific
Overall
Male
Female
Palau
Nauru
Tokelau
Lao People's Democratic Republic
Tuvalu
Myanmar
Tonga
Micronesia
Marshall Islands
Brunei Darussalam
Japan
Fiji
Viet Nam
Kiribati
Mongolia
China
New Zealand
Overall
Framingham
WHO
SCORE2
Risk Group
High Risk
Moderate Risk
Low Risk
Cumulative Percent Count

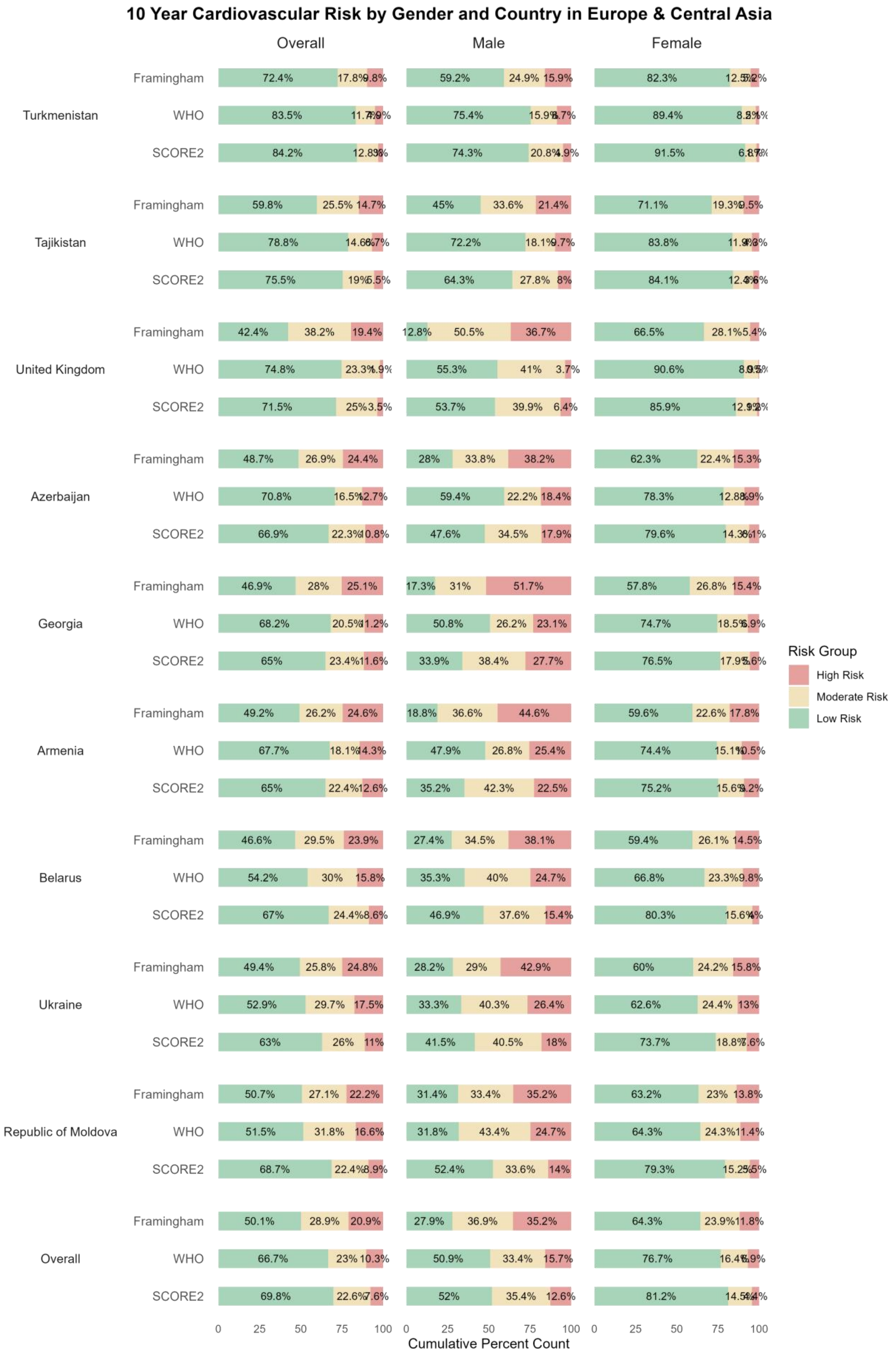
10 Year Cardiovascular Risk by Gender and Country in Europe & Central Asia
Overall
Male
Female
Turkmenistan
Framingham
72.4% 17.8%
59.2% 24.9% 15.9%
82.3%
WHO
83.5%
75.4%
89.4%
SCORE2
84.2%
74.3% 20.8%
91.5%
Tajikistan
Framingham
59.8% 25.5% 14.7%
45% 33.6% 21.4%
71.1% 19.3%
WHO
78.8%
72.2%
83.8%
SCORE2
75.5% 19%
64.3% 27.8% 8%
84.1%
United Kingdom
Framingham
42.4% 38.2% 19.4%
12.8% 50.5% 36.7%
66.5% 28.1%
WHO
74.8% 23.3%
55.3% 41% 3.7%
90.6%
SCORE2
71.5% 25%
53.7% 39.9% 6.4%
85.9%
Azerbaijan
Framingham
48.7% 26.9% 24.4%
28% 33.8% 38.2%
62.3% 22.4% 15.3%
WHO
70.8% 16.5%
59.4% 22.2% 18.4%
78.3%
SCORE2
66.9% 22.3%
47.6% 34.5% 17.9%
79.6%
Georgia
Framingham
46.9% 28% 25.1%
17.3% 31% 51.7%
57.8% 26.8% 15.4%
WHO
68.2%
50.8% 26.2% 23.1%
74.7%
SCORE2
65% 23.4%
33.9% 38.4% 27.7%
76.5%
Armenia
Framingham
49.2% 26.2% 24.6%
18.8% 36.6% 44.6%
59.6% 22.6% 17.8%
WHO
67.7%
47.9% 26.8% 25.4%
74.4%
SCORE2
65% 22.4%
35.2% 42.3% 22.5%
75.2%
Belarus
Framingham
46.6% 29.5% 23.9%
27.4% 34.5% 38.1%
59.4% 26.1% 14.5%
WHO
54.2% 30% 15.8%
35.3% 40% 24.7%
66.8%
SCORE2
67% 24.4%
46.9% 37.6% 15.4%
80.3%
Ukraine
Framingham
49.4% 25.8% 24.8%
28.2% 29% 42.9%
60% 24.2% 15.8%
WHO
52.9% 29.7% 17.5%
33.3% 40.3% 26.4%
62.6% 24.4% 13%
SCORE2
63% 26% 11%
41.5% 40.5% 18%
73.7%
Republic of Moldova
Framingham
50.7% 27.1% 22.2%
31.4% 33.4% 35.2%
63.2% 23% 13.8%
WHO
51.5% 31.8% 16.6%
31.8% 43.4% 24.7%
64.3%
SCORE2
68.7%
52.4% 33.6% 14%
79.3%
Overall
Framingham
50.1% 28.9% 20.9%
27.9% 36.9% 35.2%
64.3%
WHO
66.7% 23%
50.9% 33.4% 15.7%
76.7%
SCORE2
69.8%
52% 35.4% 12.6%
81.2%
0 25 50 75 100
Cumulative Percent Count
Risk Group
High Risk
Moderate Risk
Low Risk

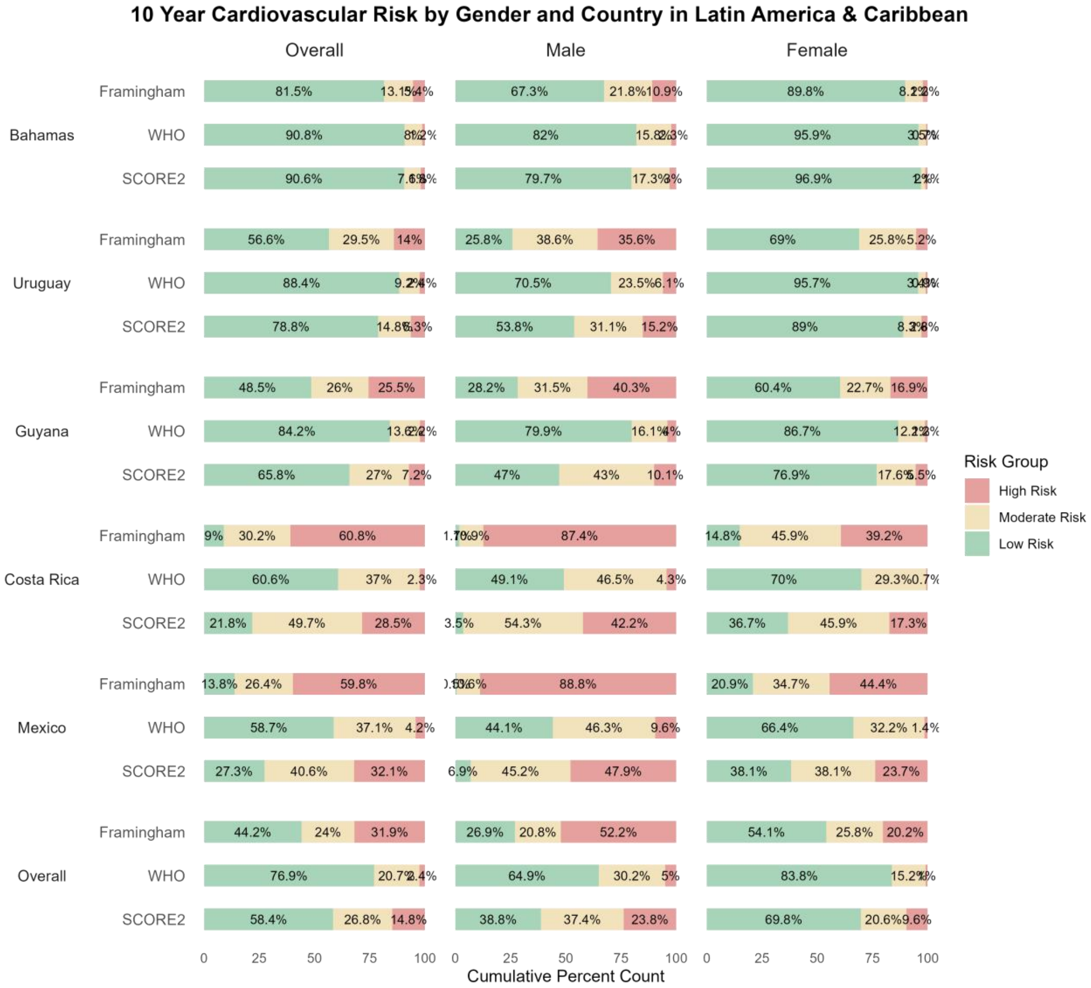
10 Year Cardiovascular Risk by Gender and Country in Latin America & Caribbean
Overall
Male
Female
Bahamas
Uruguay
Guyana
Costa Rica
Mexico
Overall
Framingham
WHO
SCORE2
Risk Group
High Risk
Moderate Risk
Low Risk
Cumulative Percent Count

10 Year Cardiovascular Risk by Gender and Country in Middle East & North Africa

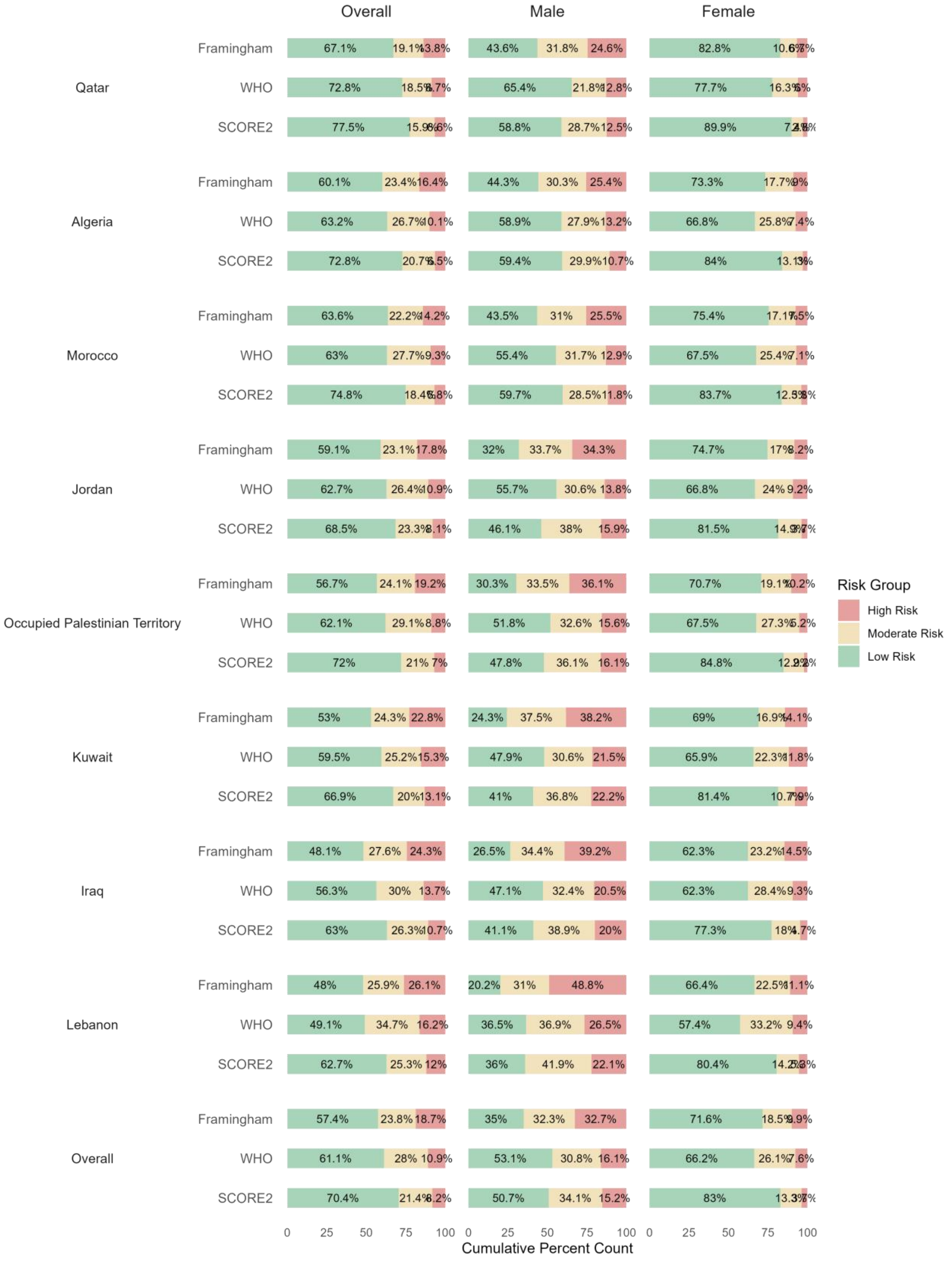

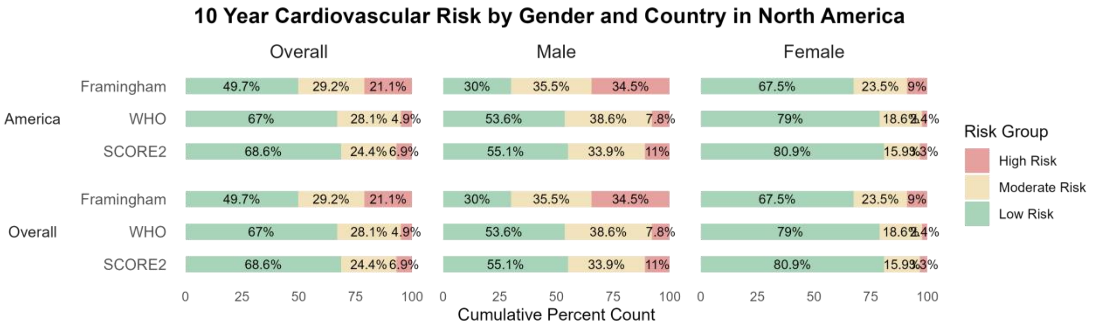
10 Year Cardiovascular Risk by Gender and Country in North America
Overall
Male
Female
America
Overall
Framingham
WHO
SCORE2
Risk Group
High Risk
Moderate Risk
Low Risk
Cumulative Percent Count

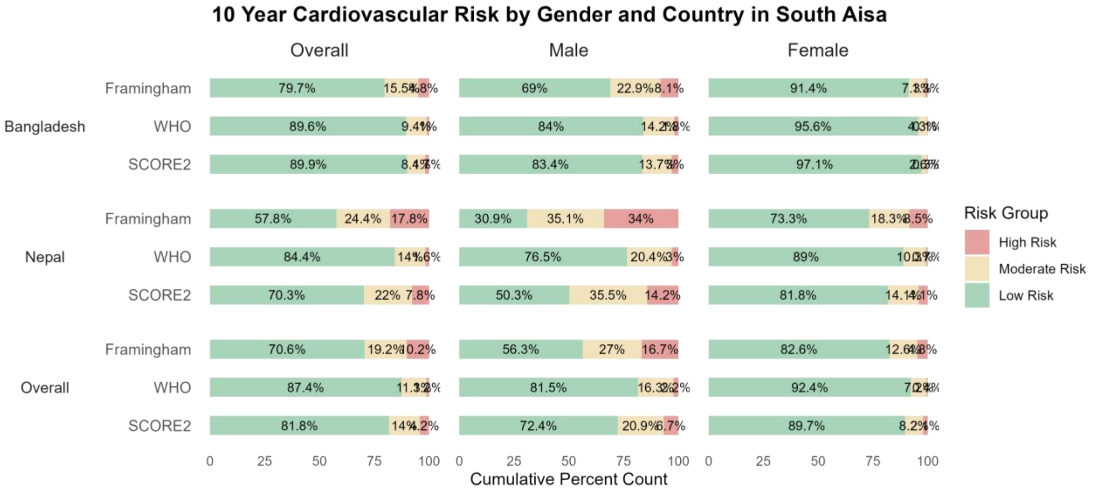
10 Year Cardiovascular Risk by Gender and Country in South Aisa
Overall
Male
Female
Bangladesh
Nepal
Overall
Framingham
WHO
SCORE2
Risk Group
High Risk
Moderate Risk
Low Risk
Cumulative Percent Count

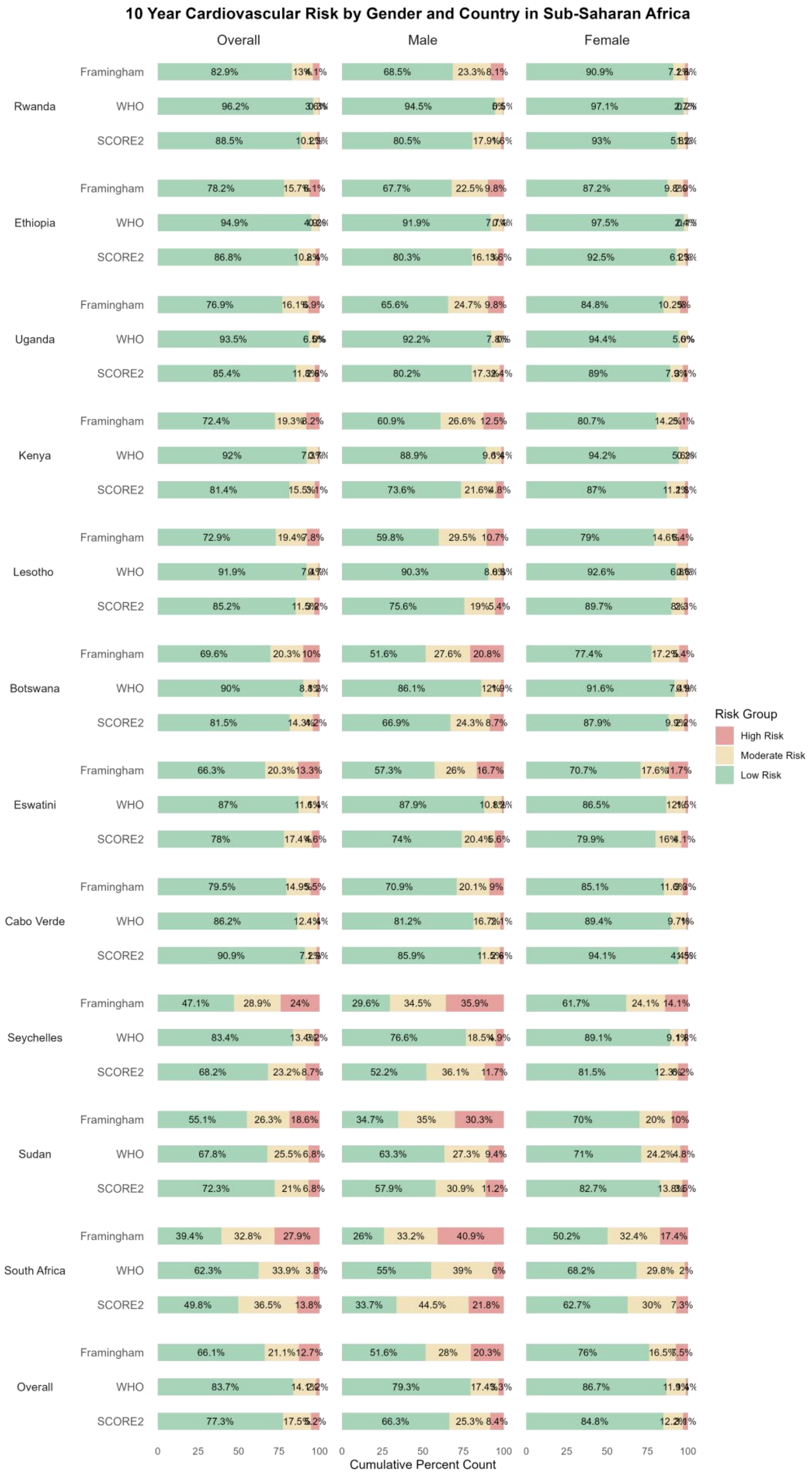

Figure S3. Distribution of 10- year cardiovascular disease risk levels across regions and countries

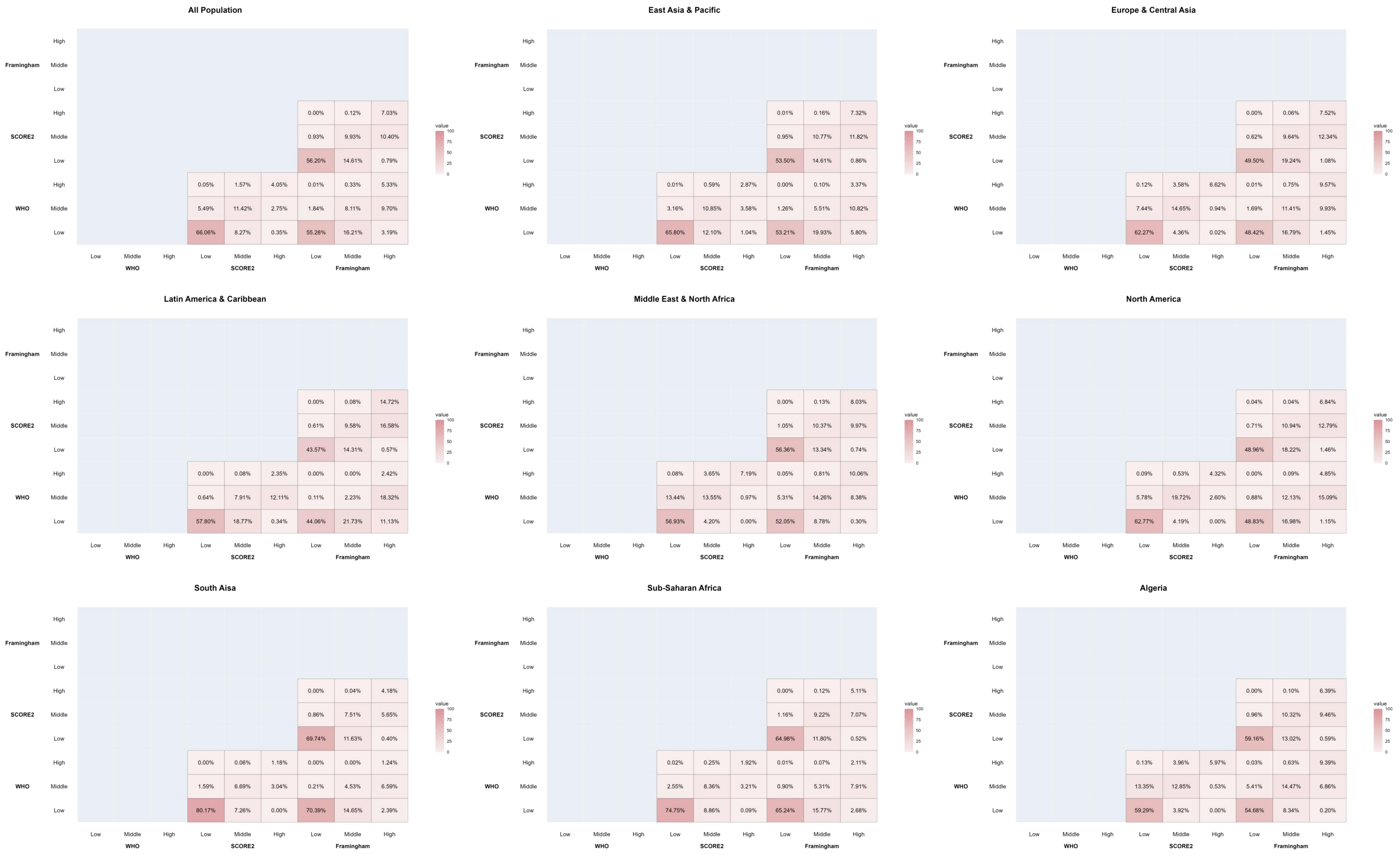
All Population
Framingham
SCORE2
WHO
High
Middle
Low
0.00% 0.12% 7.03%
0.93% 9.93% 10.40%
56.20% 14.61% 0.79%
0.05% 1.57% 4.05% 0.01% 0.33% 5.33%
5.49% 11.42% 2.75% 1.84% 8.11% 9.70%
66.06% 8.27% 0.35% 55.28% 16.21% 3.19%
value
100
75
50
25
0
East Asia & Pacific
0.01% 0.16% 7.32%
0.95% 10.77% 11.82%
53.50% 14.61% 0.86%
0.01% 0.59% 2.87% 0.00% 0.10% 3.37%
3.16% 10.85% 3.58% 1.26% 5.51% 10.82%
65.80% 12.10% 1.04% 53.21% 19.93% 5.80%
Europe & Central Asia
0.00% 0.06% 7.52%
0.62% 9.64% 12.34%
49.50% 19.24% 1.08%
0.12% 3.58% 6.62% 0.01% 0.75% 9.57%
7.44% 14.65% 0.94% 1.69% 11.41% 9.93%
62.27% 4.36% 0.02% 48.42% 16.79% 1.45%
Latin America & Caribbean
0.00% 0.08% 14.72%
0.61% 9.58% 16.58%
43.57% 14.31% 0.57%
0.00% 0.08% 2.35% 0.00% 0.00% 2.42%
0.64% 7.91% 12.11% 0.11% 2.23% 18.32%
57.80% 18.77% 0.34% 44.06% 21.73% 11.13%
Middle East & North Africa
0.00% 0.13% 8.03%
1.05% 10.37% 9.97%
56.36% 13.34% 0.74%
0.08% 3.65% 7.19% 0.05% 0.81% 10.06%
13.44% 13.55% 0.97% 5.31% 14.26% 8.38%
56.93% 4.20% 0.00% 52.05% 8.78% 0.30%
North America
0.04% 0.04% 6.84%
0.71% 10.94% 12.79%
48.96% 18.22% 1.46%
0.09% 0.53% 4.32% 0.00% 0.09% 4.85%
5.78% 19.72% 2.60% 0.88% 12.13% 15.09%
62.77% 4.19% 0.00% 48.83% 16.98% 1.15%
South Aisa
0.00% 0.04% 4.18%
0.86% 7.51% 5.65%
69.74% 11.63% 0.40%
0.00% 0.06% 1.18% 0.00% 0.00% 1.24%
1.59% 6.69% 3.04% 0.21% 4.53% 6.59%
80.17% 7.26% 0.00% 70.39% 14.65% 2.39%
Sub-Saharan Africa
0.00% 0.12% 5.11%
1.16% 9.22% 7.07%
64.98% 11.80% 0.52%
0.02% 0.25% 1.92% 0.01% 0.07% 2.11%
2.55% 8.36% 3.21% 0.90% 5.31% 7.91%
74.75% 8.86% 0.09% 65.24% 15.77% 2.68%
Algeria
0.00% 0.10% 6.39%
0.96% 10.32% 9.46%
59.16% 13.02% 0.59%
0.13% 3.96% 5.97% 0.03% 0.63% 9.39%
13.35% 12.85% 0.53% 5.41% 14.47% 6.86%
59.29% 3.92% 0.00% 54.68% 8.34% 0.20%

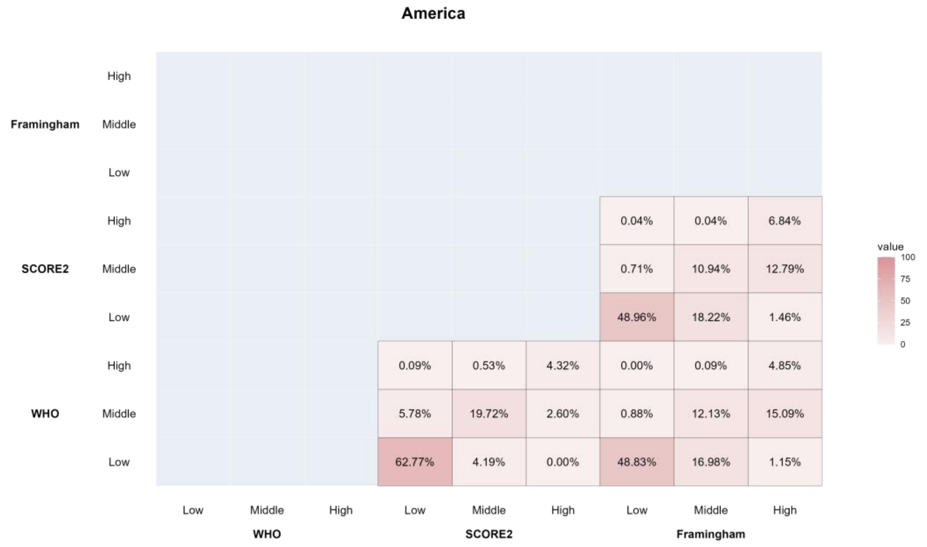
America
Framingham
SCORE2
WHO
High
Middle
Low
0.04% 0.04% 6.84%
0.71% 10.94% 12.79%
48.96% 18.22% 1.46%
0.09% 0.53% 4.32% 0.00% 0.09% 4.85%
5.78% 19.72% 2.60% 0.88% 12.13% 15.09%
62.77% 4.19% 0.00% 48.83% 16.98% 1.15%
value
100
75
50
25
0

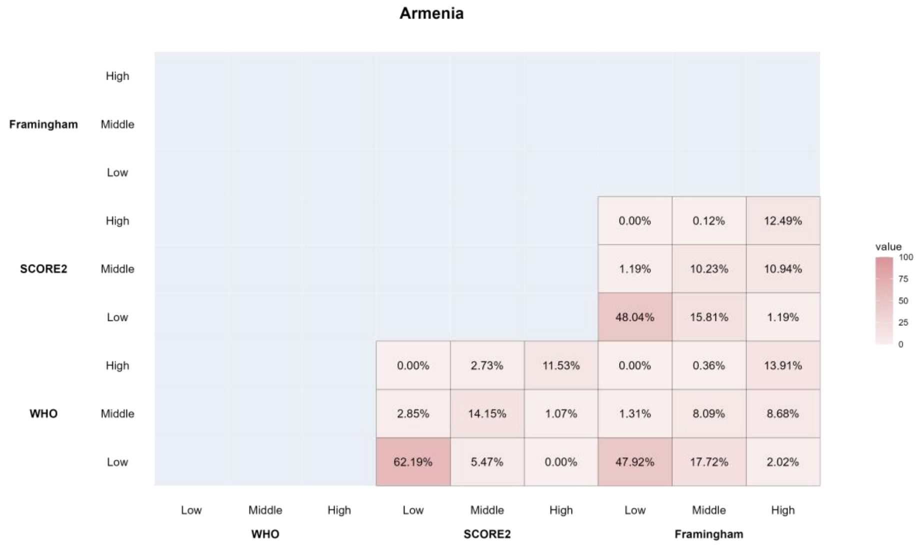
Armenia
Framingham
SCORE2
WHO
High
Middle
Low
0.00% 0.12% 12.49%
1.19% 10.23% 10.94%
48.04% 15.81% 1.19%
0.00% 2.73% 11.53% 0.00% 0.36% 13.91%
2.85% 14.15% 1.07% 1.31% 8.09% 8.68%
62.19% 5.47% 0.00% 47.92% 17.72% 2.02%
value
100
75
50
25
0

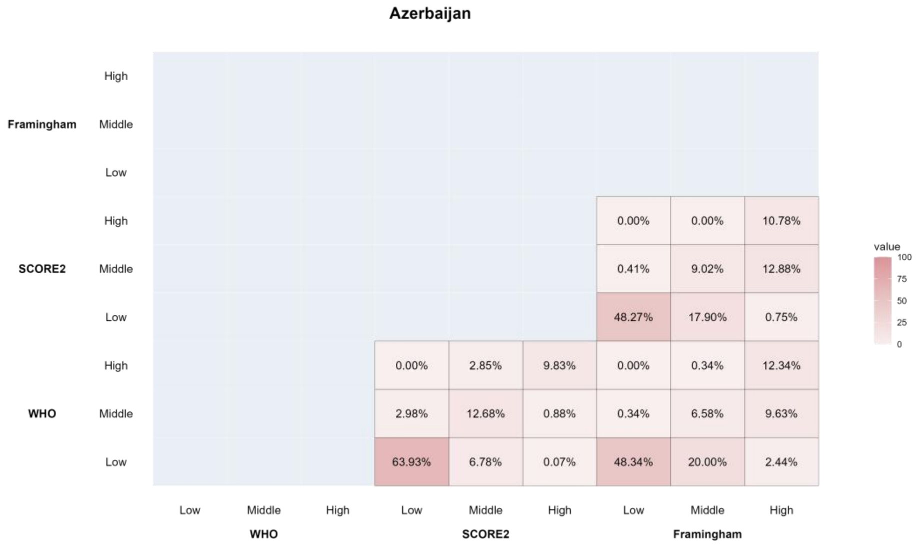
Azerbaijan
Framingham
SCORE2
WHO
High
Middle
Low
0.00% 0.00% 10.78%
0.41% 9.02% 12.88%
48.27% 17.90% 0.75%
0.00% 2.85% 9.83% 0.00% 0.34% 12.34%
2.98% 12.68% 0.88% 0.34% 6.58% 9.63%
63.93% 6.78% 0.07% 48.34% 20.00% 2.44%
value
100
75
50
25
0

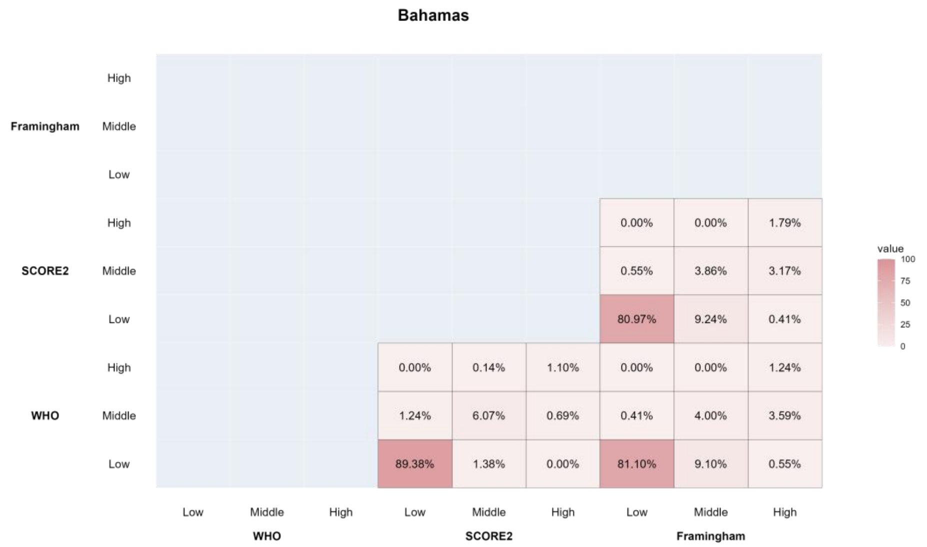
Bahamas
Framingham
SCORE2
WHO
High
Middle
Low
0.00% 0.00% 1.79%
0.55% 3.86% 3.17%
80.97% 9.24% 0.41%
0.00% 0.14% 1.10% 0.00% 0.00% 1.24%
1.24% 6.07% 0.69% 0.41% 4.00% 3.59%
89.38% 1.38% 0.00% 81.10% 9.10% 0.55%
value
100
75
50
25
0

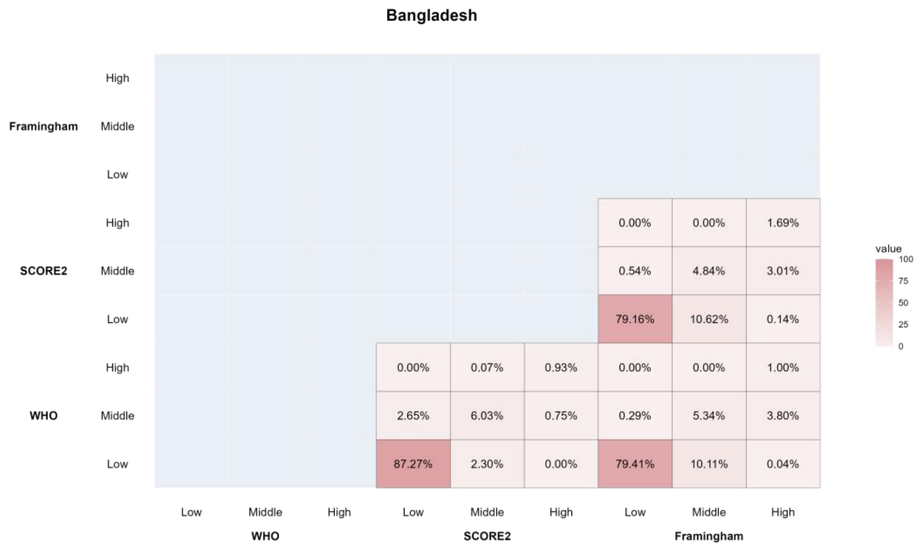
Bangladesh
Framingham
SCORE2
WHO
High
Middle
Low
0.00% 0.00% 1.69%
0.54% 4.84% 3.01%
79.16% 10.62% 0.14%
0.00% 0.07% 0.93% 0.00% 0.00% 1.00%
2.65% 6.03% 0.75% 0.29% 5.34% 3.80%
87.27% 2.30% 0.00% 79.41% 10.11% 0.04%
value
100
75
50
25
0

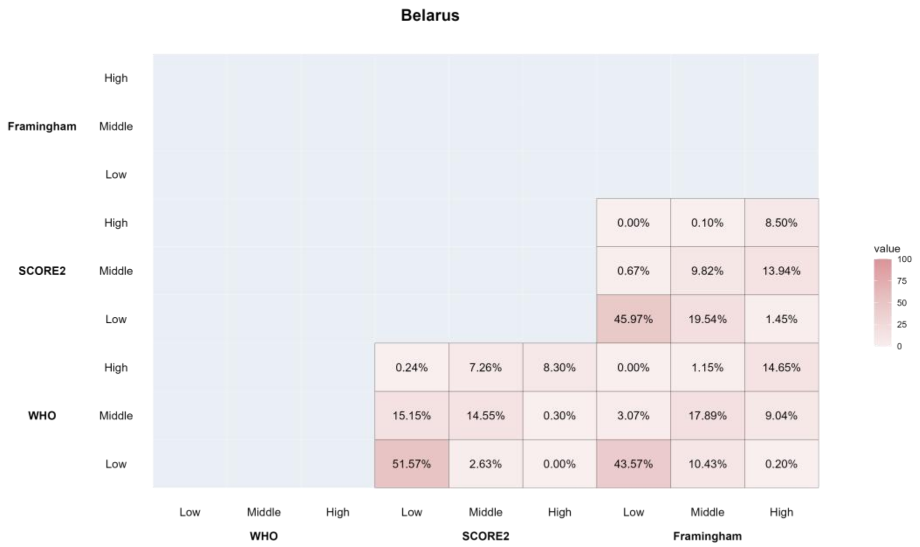
Belarus
Framingham
SCORE2
WHO
High
Middle
Low
0.00% 0.10% 8.50%
0.67% 9.82% 13.94%
45.97% 19.54% 1.45%
0.24% 7.26% 8.30% 0.00% 1.15% 14.65%
15.15% 14.55% 0.30% 3.07% 17.89% 9.04%
51.57% 2.63% 0.00% 43.57% 10.43% 0.20%
value
100
75
50
25
0

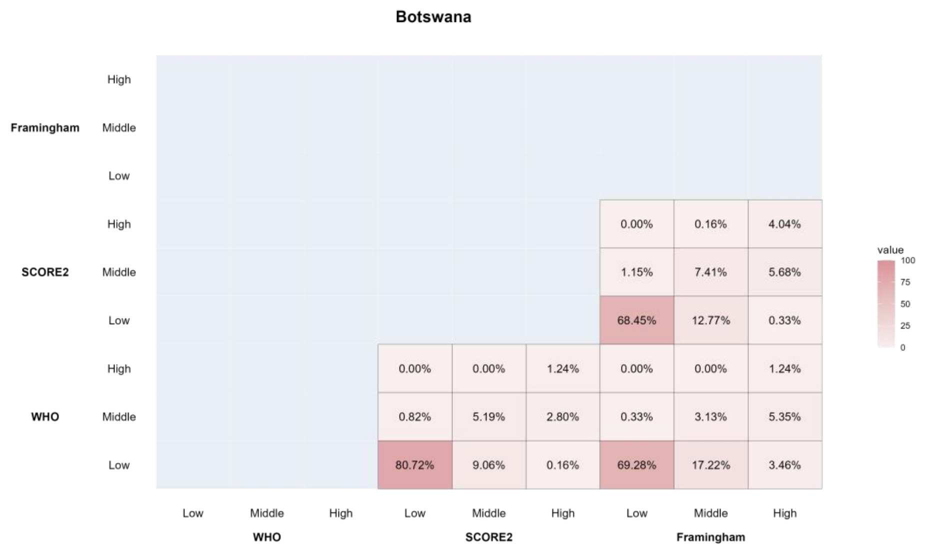
Botswana
Framingham
SCORE2
WHO
High
Middle
Low
0.00% 0.16% 4.04%
1.15% 7.41% 5.68%
68.45% 12.77% 0.33%
0.00% 0.00% 1.24% 0.00% 0.00% 1.24%
0.82% 5.19% 2.80% 0.33% 3.13% 5.35%
80.72% 9.06% 0.16% 69.28% 17.22% 3.46%
value
100
75
50
25
0

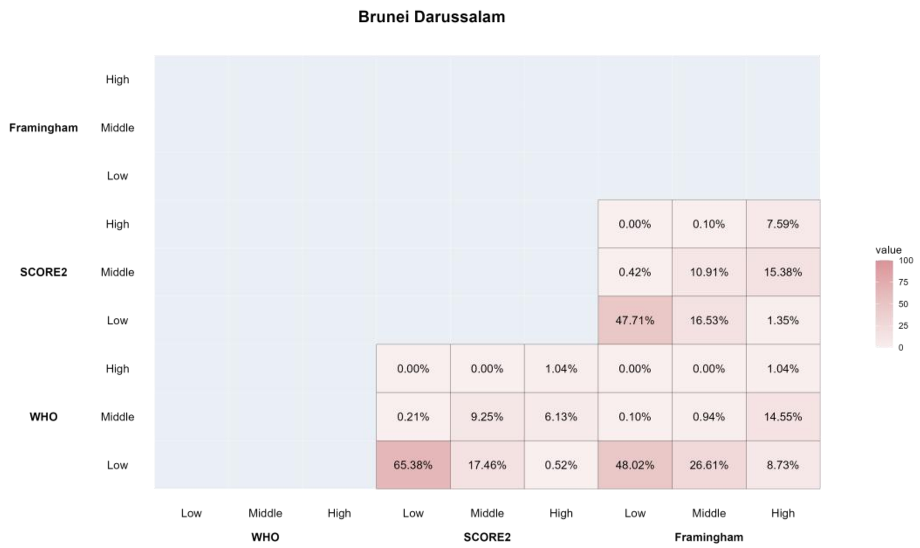
Brunei Darussalam
Framingham
SCORE2
WHO
High
Middle
Low
0.00% 0.10% 7.59%
0.42% 10.91% 15.38%
47.71% 16.53% 1.35%
0.00% 0.00% 1.04% 0.00% 0.00% 1.04%
0.21% 9.25% 6.13% 0.10% 0.94% 14.55%
65.38% 17.46% 0.52% 48.02% 26.61% 8.73%
value
100
75
50
25
0

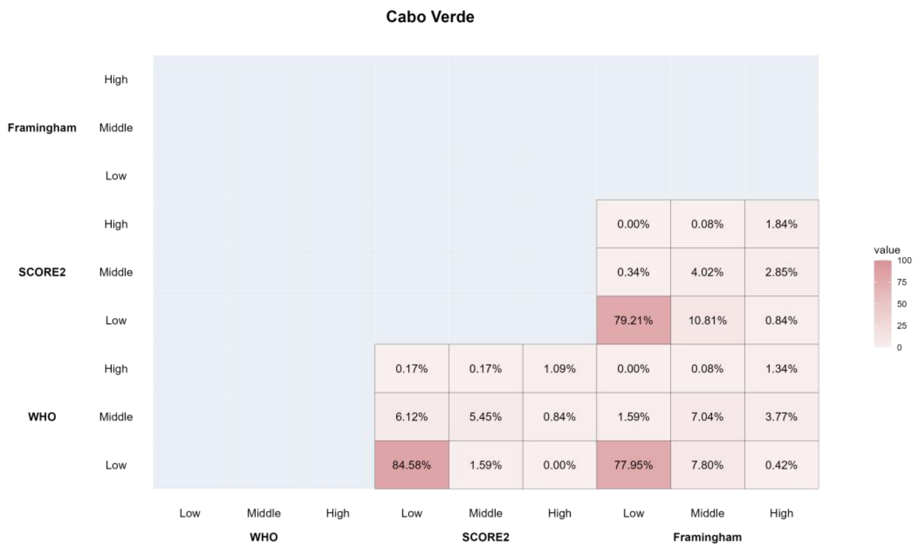
Cabo Verde
Framingham
SCORE2
WHO
High
Middle
Low
0.00% 0.08% 1.84%
0.34% 4.02% 2.85%
79.21% 10.81% 0.84%
0.17% 0.17% 1.09% 0.00% 0.08% 1.34%
6.12% 5.45% 0.84% 1.59% 7.04% 3.77%
84.58% 1.59% 0.00% 77.95% 7.80% 0.42%
value
100
75
50
25
0

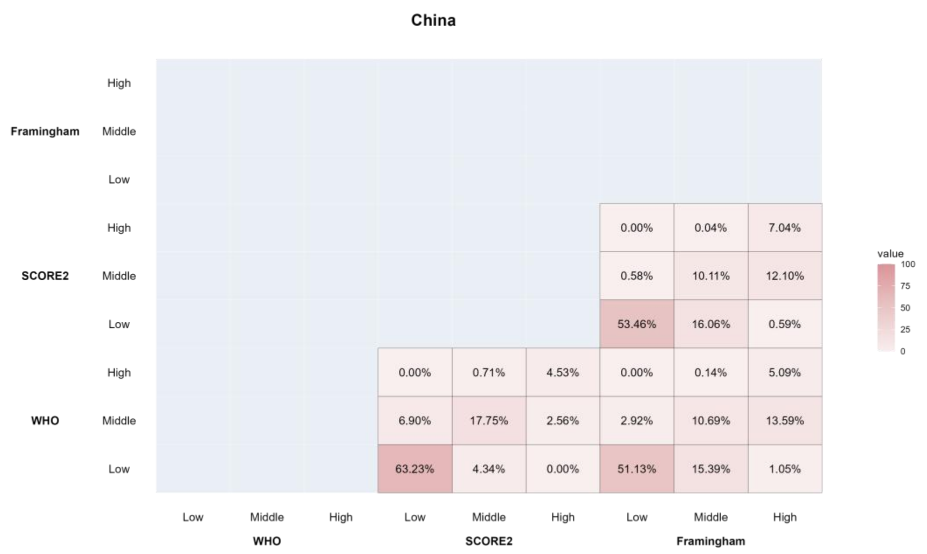
China
Framingham High Middle Low
SCORE2 High 0.00% 0.04% 7.04%
SCORE2 Middle 0.58% 10.11% 12.10%
SCORE2 Low 53.46% 16.06% 0.59%
WHO High 0.00% 0.71% 4.53% 0.00% 0.14% 5.09%
WHO Middle 6.90% 17.75% 2.56% 2.92% 10.69% 13.59%
WHO Low 63.23% 4.34% 0.00% 51.13% 15.39% 1.05%
Low Middle High Low Middle High Low Middle High
WHO SCORE2 Framingham
value 100 75 50 25 0

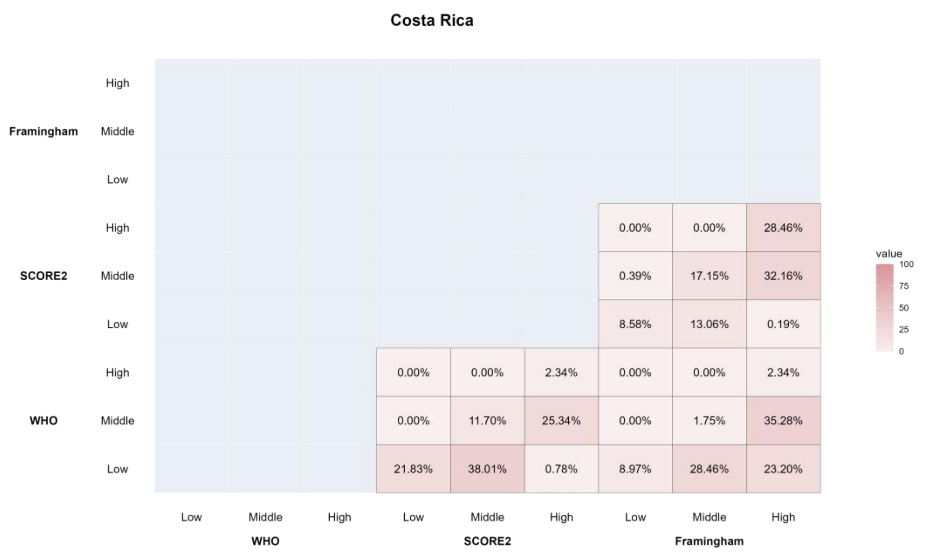
Costa Rica
Framingham High Middle Low
SCORE2 High 0.00% 0.00% 28.46%
SCORE2 Middle 0.39% 17.15% 32.16%
SCORE2 Low 8.58% 13.06% 0.19%
WHO High 0.00% 0.00% 2.34% 0.00% 0.00% 2.34%
WHO Middle 0.00% 11.70% 25.34% 0.00% 1.75% 35.28%
WHO Low 21.83% 38.01% 0.78% 8.97% 28.46% 23.20%
Low Middle High Low Middle High Low Middle High
WHO SCORE2 Framingham
value 100 75 50 25 0

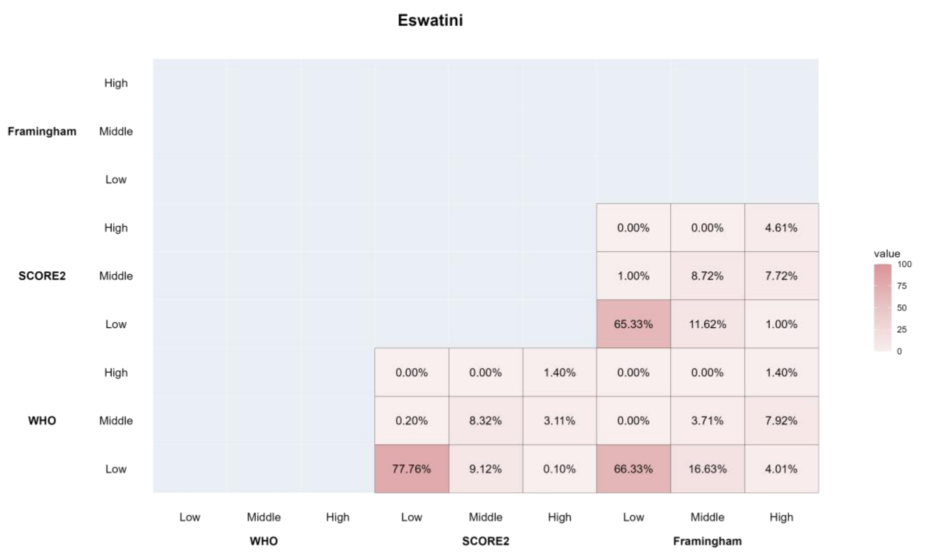
Eswatini
Framingham High Middle Low
SCORE2 High 0.00% 0.00% 4.61%
SCORE2 Middle 1.00% 8.72% 7.72%
SCORE2 Low 65.33% 11.62% 1.00%
WHO High 0.00% 0.00% 1.40% 0.00% 0.00% 1.40%
WHO Middle 0.20% 8.32% 3.11% 0.00% 3.71% 7.92%
WHO Low 77.76% 9.12% 0.10% 66.33% 16.63% 4.01%
Low Middle High Low Middle High Low Middle High
WHO SCORE2 Framingham
value 100 75 50 25 0

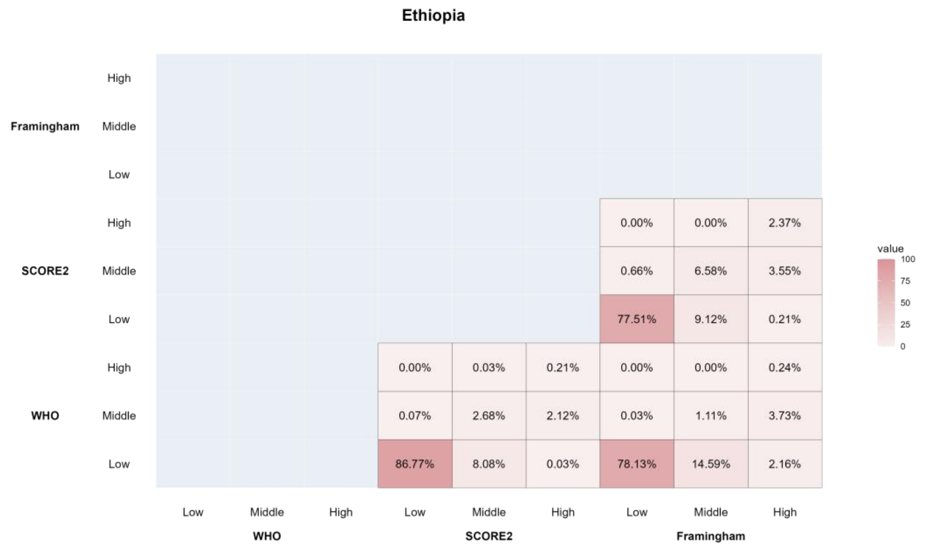
Ethiopia
Framingham High Middle Low
SCORE2 High 0.00% 0.00% 2.37%
SCORE2 Middle 0.66% 6.58% 3.55%
SCORE2 Low 77.51% 9.12% 0.21%
WHO High 0.00% 0.03% 0.21% 0.00% 0.00% 0.24%
WHO Middle 0.07% 2.68% 2.12% 0.03% 1.11% 3.73%
WHO Low 86.77% 8.08% 0.03% 78.13% 14.59% 2.16%
Low Middle High Low Middle High Low Middle High
WHO SCORE2 Framingham
value 100 75 50 25 0

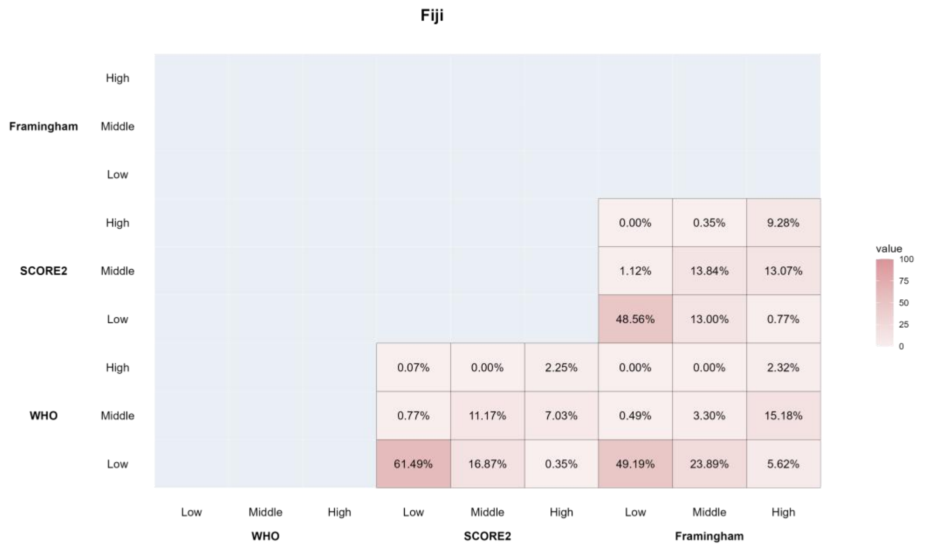
Fiji
Framingham High Middle Low
SCORE2 High 0.00% 0.35% 9.28%
SCORE2 Middle 1.12% 13.84% 13.07%
SCORE2 Low 48.56% 13.00% 0.77%
WHO High 0.07% 0.00% 2.25% 0.00% 0.00% 2.32%
WHO Middle 0.77% 11.17% 7.03% 0.49% 3.30% 15.18%
WHO Low 61.49% 16.87% 0.35% 49.19% 23.89% 5.62%
Low Middle High Low Middle High Low Middle High
WHO SCORE2 Framingham
value 100 75 50 25 0

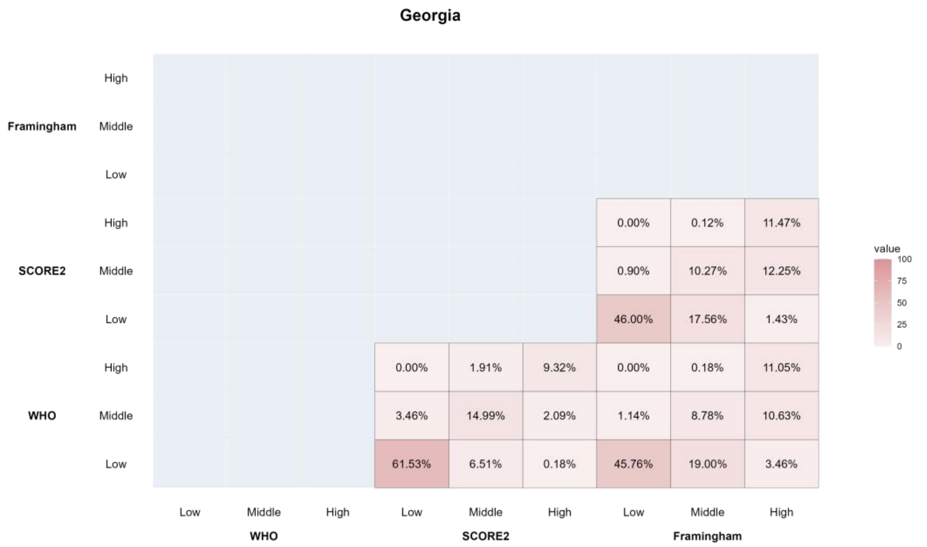
Georgia
Framingham High Middle Low
SCORE2 High 0.00% 0.12% 11.47%
SCORE2 Middle 0.90% 10.27% 12.25%
SCORE2 Low 46.00% 17.56% 1.43%
WHO High 0.00% 1.91% 9.32% 0.00% 0.18% 11.05%
WHO Middle 3.46% 14.99% 2.09% 1.14% 8.78% 10.63%
WHO Low 61.53% 6.51% 0.18% 45.76% 19.00% 3.46%
Low Middle High Low Middle High Low Middle High
WHO SCORE2 Framingham
value 100 75 50 25 0

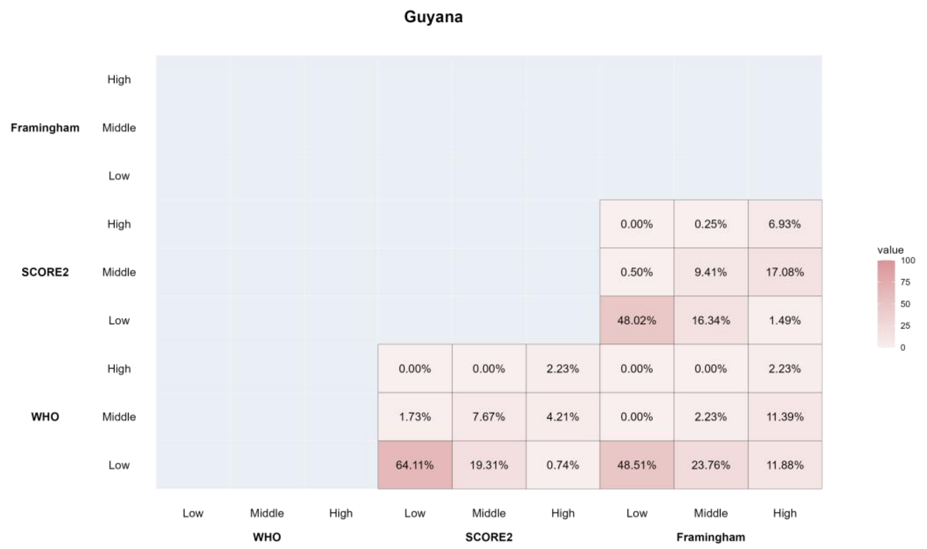
Guyana
Framingham High Middle Low
SCORE2 High 0.00% 0.25% 6.93%
SCORE2 Middle 0.50% 9.41% 17.08%
SCORE2 Low 48.02% 16.34% 1.49%
WHO High 0.00% 0.00% 2.23% 0.00% 0.00% 2.23%
WHO Middle 1.73% 7.67% 4.21% 0.00% 2.23% 11.39%
WHO Low 64.11% 19.31% 0.74% 48.51% 23.76% 11.88%
Low Middle High Low Middle High Low Middle High
WHO SCORE2 Framingham
value 100 75 50 25 0

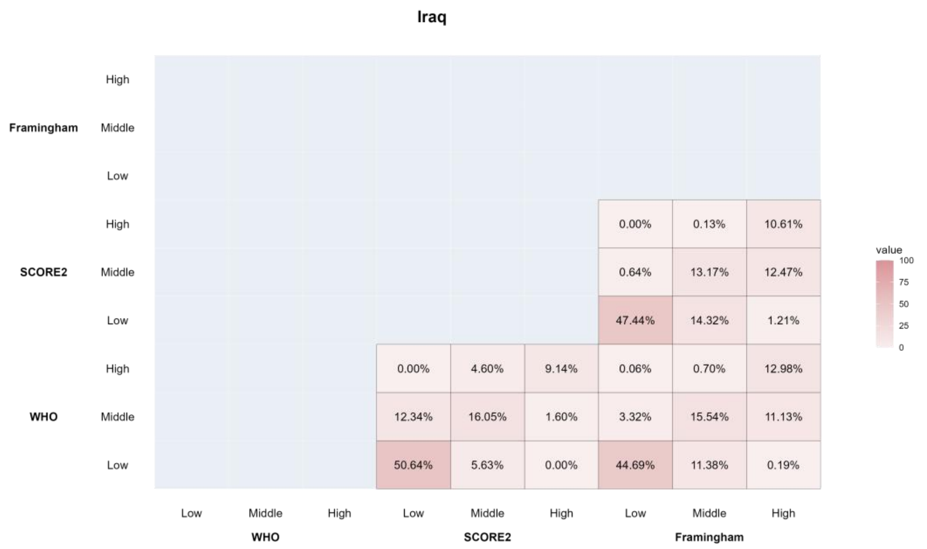
Iraq
Framingham High Middle Low
SCORE2 High 0.00% 0.13% 10.61%
SCORE2 Middle 0.64% 13.17% 12.47%
SCORE2 Low 47.44% 14.32% 1.21%
WHO High 0.00% 4.60% 9.14% 0.06% 0.70% 12.98%
WHO Middle 12.34% 16.05% 1.60% 3.32% 15.54% 11.13%
WHO Low 50.64% 5.63% 0.00% 44.69% 11.38% 0.19%
Low Middle High Low Middle High Low Middle High
WHO SCORE2 Framingham
value 100 75 50 25 0

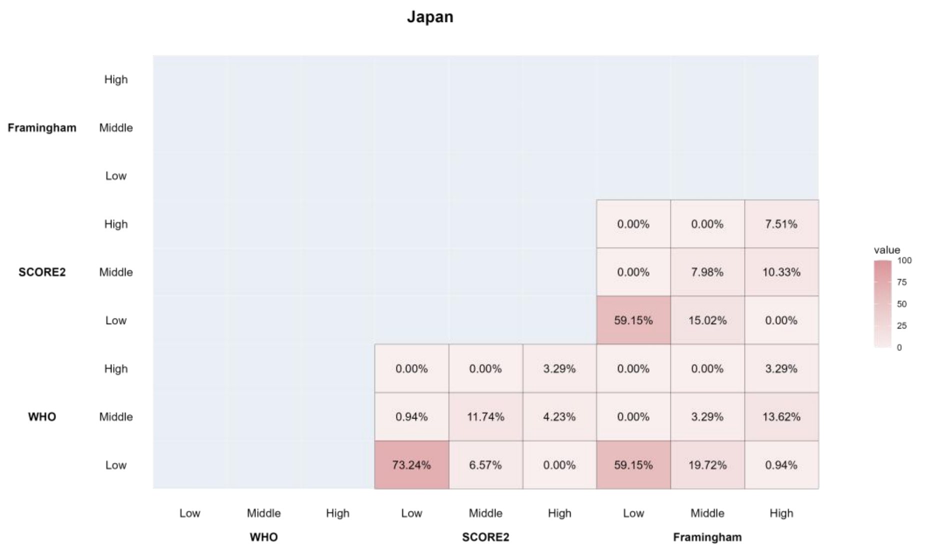
Japan
Framingham High Middle Low
SCORE2 High 0.00% 0.00% 7.51%
SCORE2 Middle 0.00% 7.98% 10.33%
SCORE2 Low 59.15% 15.02% 0.00%
WHO High 0.00% 0.00% 3.29% 0.00% 0.00% 3.29%
WHO Middle 0.94% 11.74% 4.23% 0.00% 3.29% 13.62%
WHO Low 73.24% 6.57% 0.00% 59.15% 19.72% 0.94%
Low Middle High Low Middle High Low Middle High
WHO SCORE2 Framingham
value 100 75 50 25 0

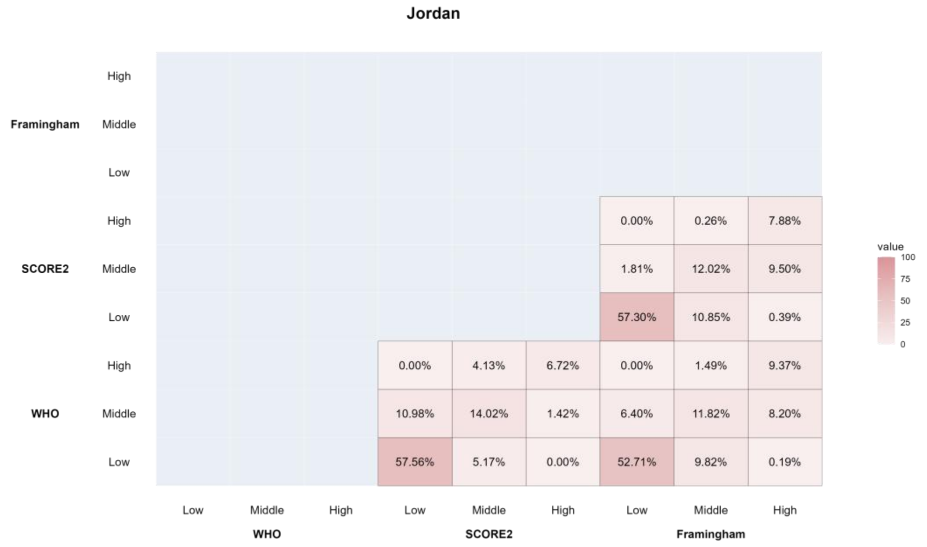
Jordan
Framingham
SCORE2
WHO
High
Middle
Low
0.00% 0.26% 7.88%
1.81% 12.02% 9.50%
57.30% 10.85% 0.39%
0.00% 4.13% 6.72% 0.00% 1.49% 9.37%
10.98% 14.02% 1.42% 6.40% 11.82% 8.20%
57.56% 5.17% 0.00% 52.71% 9.82% 0.19%
Low Middle High Low Middle High Low Middle High
WHO SCORE2 Framingham
value
100
75
50
25
0

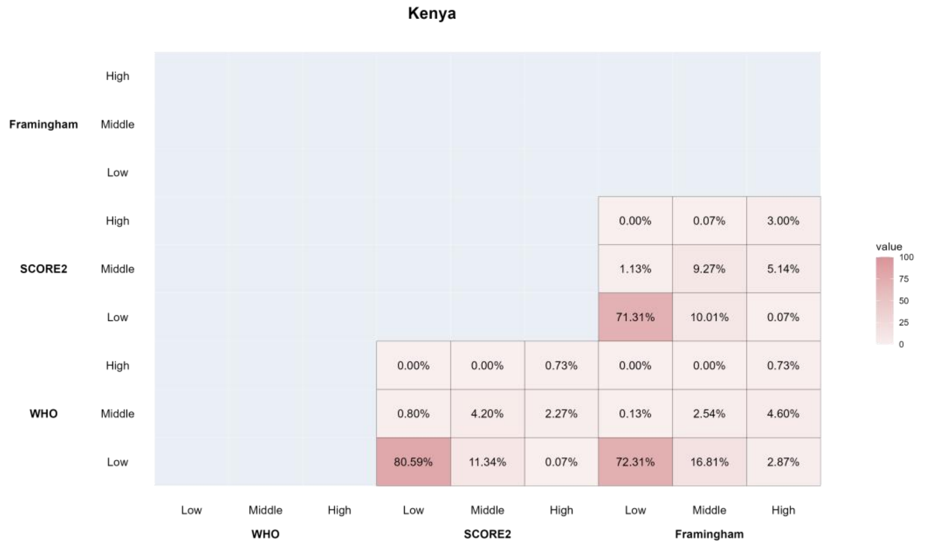
Kenya
Framingham
SCORE2
WHO
High
Middle
Low
0.00% 0.07% 3.00%
1.13% 9.27% 5.14%
71.31% 10.01% 0.07%
0.00% 0.00% 0.73% 0.00% 0.00% 0.73%
0.80% 4.20% 2.27% 0.13% 2.54% 4.60%
80.59% 11.34% 0.07% 72.31% 16.81% 2.87%
Low Middle High Low Middle High Low Middle High
WHO SCORE2 Framingham
value
100
75
50
25
0

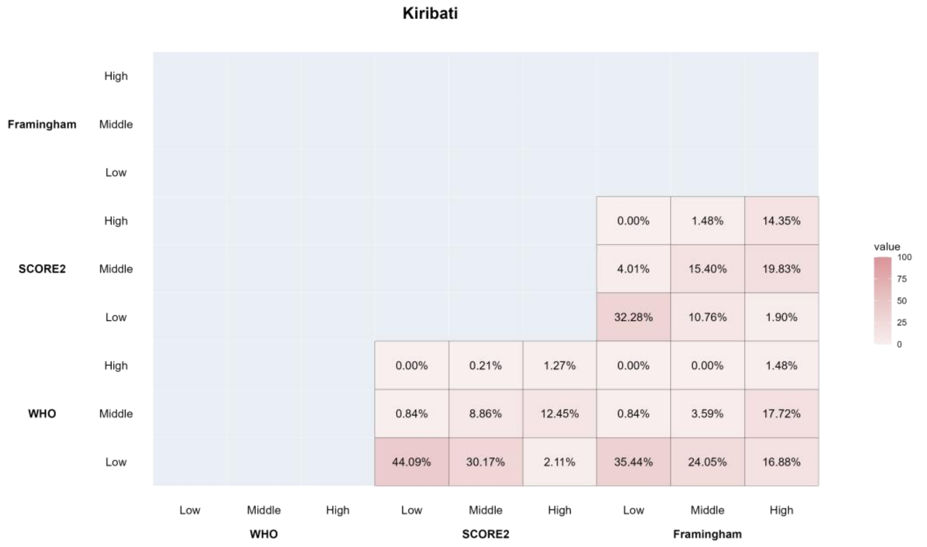
Kiribati
Framingham
SCORE2
WHO
High
Middle
Low
0.00% 1.48% 14.35%
4.01% 15.40% 19.83%
32.28% 10.76% 1.90%
0.00% 0.21% 1.27% 0.00% 0.00% 1.48%
0.84% 8.86% 12.45% 0.84% 3.59% 17.72%
44.09% 30.17% 2.11% 35.44% 24.05% 16.88%
Low Middle High Low Middle High Low Middle High
WHO SCORE2 Framingham
value
100
75
50
25
0

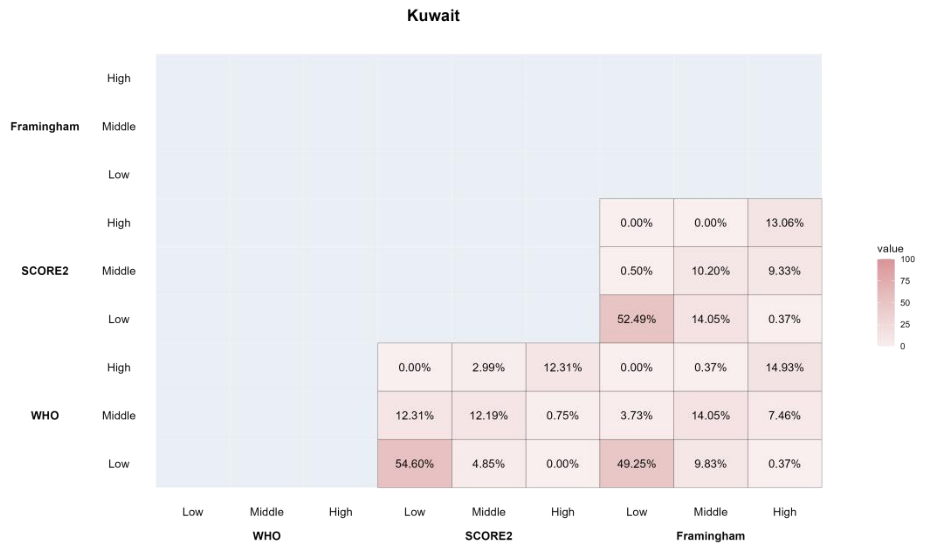
Kuwait
Framingham
SCORE2
WHO
High
Middle
Low
0.00% 0.00% 13.06%
0.50% 10.20% 9.33%
52.49% 14.05% 0.37%
0.00% 2.99% 12.31% 0.00% 0.37% 14.93%
12.31% 12.19% 0.75% 3.73% 14.05% 7.46%
54.60% 4.85% 0.00% 49.25% 9.83% 0.37%
Low Middle High Low Middle High Low Middle High
WHO SCORE2 Framingham
value
100
75
50
25
0

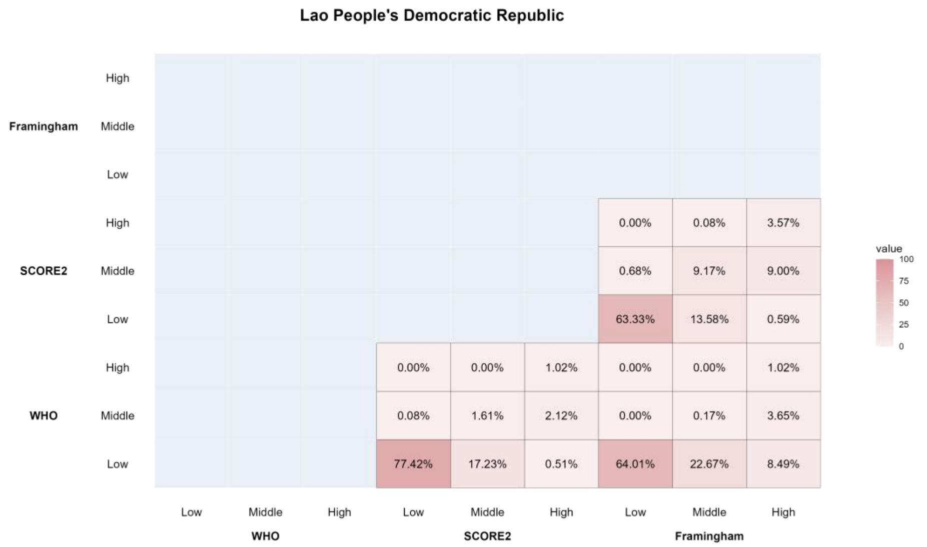
Lao People's Democratic Republic
Framingham
SCORE2
WHO
High
Middle
Low
0.00% 0.08% 3.57%
0.68% 9.17% 9.00%
63.33% 13.58% 0.59%
0.00% 0.00% 1.02% 0.00% 0.00% 1.02%
0.08% 1.61% 2.12% 0.00% 0.17% 3.65%
77.42% 17.23% 0.51% 64.01% 22.67% 8.49%
Low Middle High Low Middle High Low Middle High
WHO SCORE2 Framingham
value
100
75
50
25
0

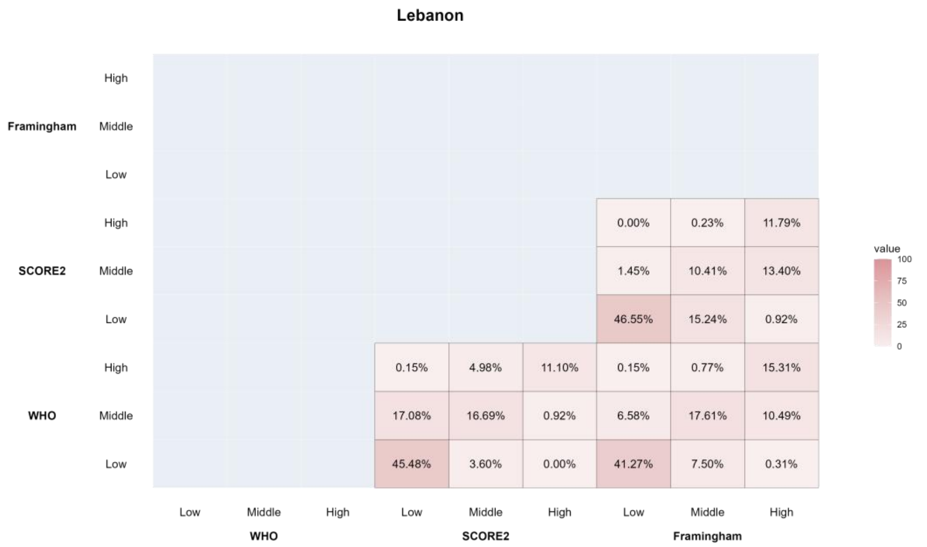
Lebanon
Framingham
SCORE2
WHO
High
Middle
Low
0.00% 0.23% 11.79%
1.45% 10.41% 13.40%
46.55% 15.24% 0.92%
0.15% 4.98% 11.10% 0.15% 0.77% 15.31%
17.08% 16.69% 0.92% 6.58% 17.61% 10.49%
45.48% 3.60% 0.00% 41.27% 7.50% 0.31%
Low Middle High Low Middle High Low Middle High
WHO SCORE2 Framingham
value
100
75
50
25
0

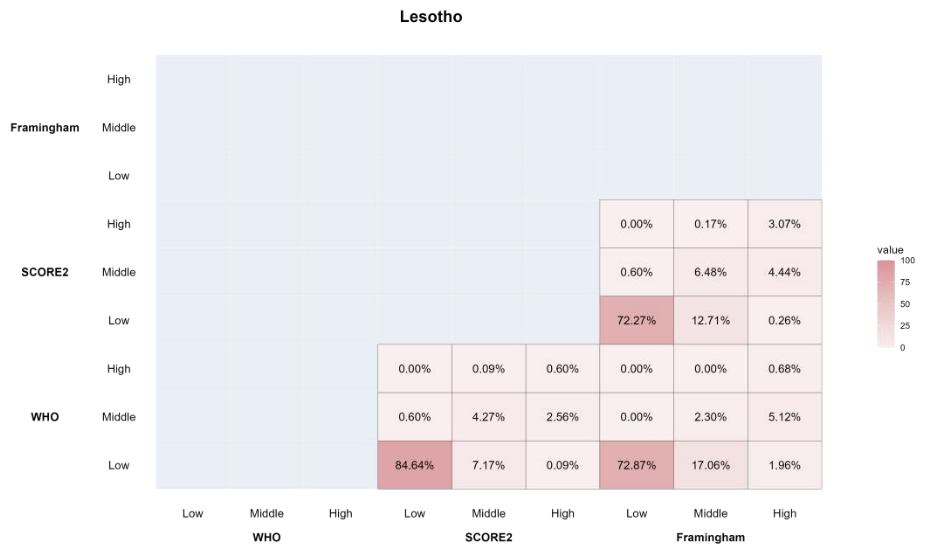
Lesotho
Framingham
SCORE2
WHO
High
Middle
Low
0.00% 0.17% 3.07%
0.60% 6.48% 4.44%
72.27% 12.71% 0.26%
0.00% 0.09% 0.60% 0.00% 0.00% 0.68%
0.60% 4.27% 2.56% 0.00% 2.30% 5.12%
84.64% 7.17% 0.09% 72.87% 17.06% 1.96%
Low Middle High Low Middle High Low Middle High
WHO SCORE2 Framingham
value
100
75
50
25
0

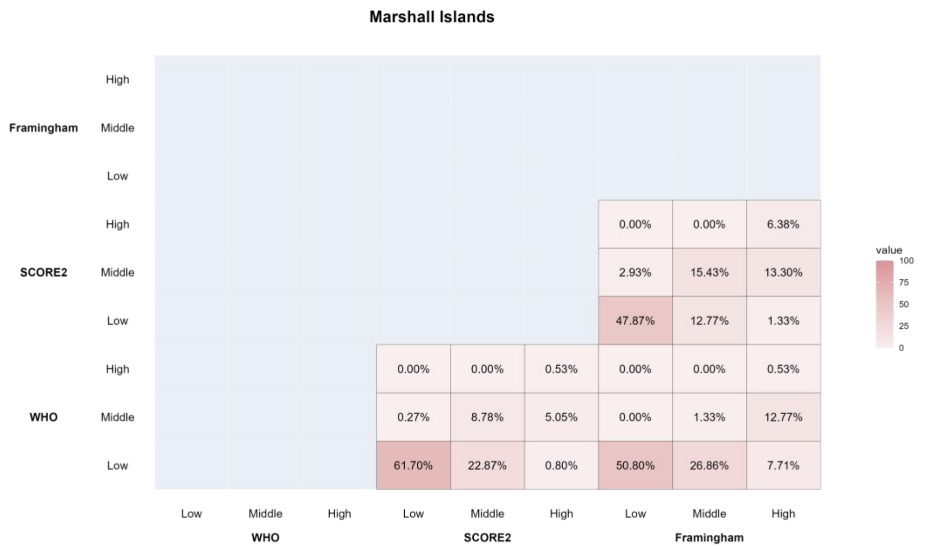
Marshall Islands
Framingham
SCORE2
WHO
High
Middle
Low
0.00% 0.00% 6.38%
2.93% 15.43% 13.30%
47.87% 12.77% 1.33%
0.00% 0.00% 0.53% 0.00% 0.00% 0.53%
0.27% 8.78% 5.05% 0.00% 1.33% 12.77%
61.70% 22.87% 0.80% 50.80% 26.86% 7.71%
Low Middle High Low Middle High Low Middle High
WHO SCORE2 Framingham
value
100
75
50
25
0

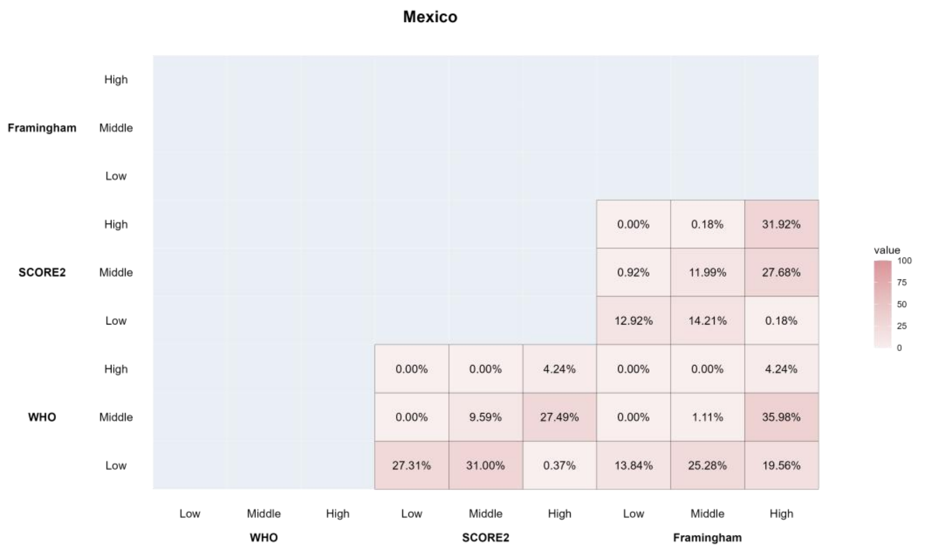
Mexico
Framingham
SCORE2
WHO
High
Middle
Low
0.00% 0.18% 31.92%
0.92% 11.99% 27.68%
12.92% 14.21% 0.18%
0.00% 0.00% 4.24% 0.00% 0.00% 4.24%
0.00% 9.59% 27.49% 0.00% 1.11% 35.98%
27.31% 31.00% 0.37% 13.84% 25.28% 19.56%
Low Middle High Low Middle High Low Middle High
WHO SCORE2 Framingham
value
100
75
50
25
0

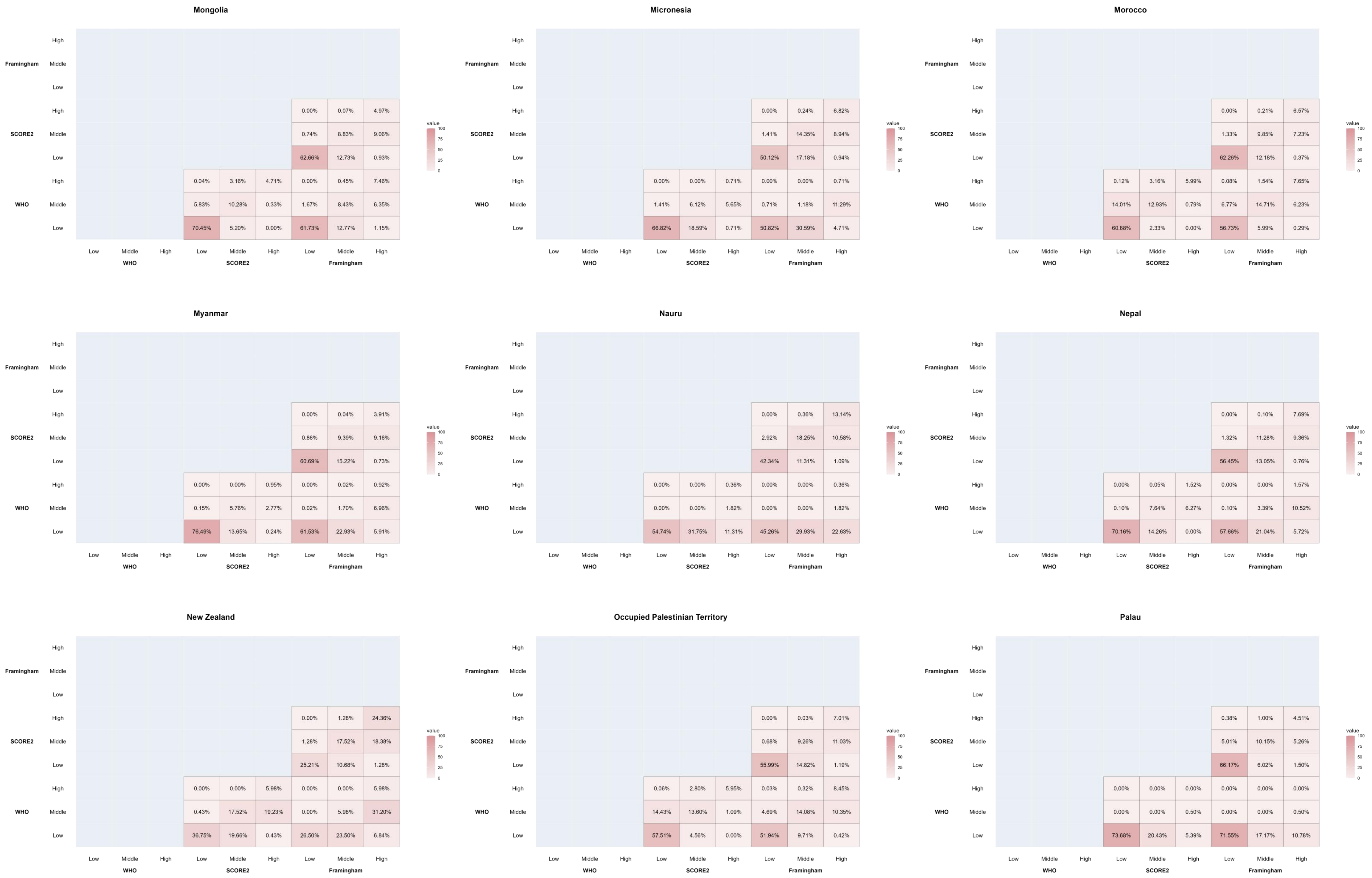
Mongolia
0.00% 0.07% 4.97%
0.74% 8.83% 9.06%
62.66% 12.73% 0.93%
0.04% 3.16% 4.71% 0.00% 0.45% 7.46%
5.83% 10.28% 0.33% 1.67% 8.43% 6.35%
70.45% 5.20% 0.00% 61.73% 12.77% 1.15%
Micronesia
0.00% 0.24% 6.82%
1.41% 14.35% 8.94%
50.12% 17.18% 0.94%
0.00% 0.00% 0.71% 0.00% 0.00% 0.71%
1.41% 6.12% 5.65% 0.71% 1.18% 11.29%
66.82% 18.59% 0.71% 50.82% 30.59% 4.71%
Morocco
0.00% 0.21% 6.57%
1.33% 9.85% 7.23%
62.26% 12.18% 0.37%
0.12% 3.16% 5.99% 0.08% 1.54% 7.65%
14.01% 12.93% 0.79% 6.77% 14.71% 6.23%
60.68% 2.33% 0.00% 56.73% 5.99% 0.29%
Myanmar
0.00% 0.04% 3.91%
0.86% 9.39% 9.16%
60.69% 15.22% 0.73%
0.00% 0.00% 0.95% 0.00% 0.02% 0.92%
0.15% 5.76% 2.77% 0.02% 1.70% 6.96%
76.49% 13.65% 0.24% 61.53% 22.93% 5.91%
Nauru
0.00% 0.36% 13.14%
2.92% 18.25% 10.58%
42.34% 11.31% 1.09%
0.00% 0.00% 0.36% 0.00% 0.00% 0.36%
0.00% 0.00% 1.82% 0.00% 0.00% 1.82%
54.74% 31.75% 11.31% 45.26% 29.93% 22.63%
Nepal
0.00% 0.10% 7.69%
1.32% 11.28% 9.36%
56.45% 13.05% 0.76%
0.00% 0.05% 1.52% 0.00% 0.00% 1.57%
0.10% 7.64% 6.27% 0.10% 3.39% 10.52%
70.16% 14.26% 0.00% 57.66% 21.04% 5.72%
New Zealand
0.00% 1.28% 24.36%
1.28% 17.52% 18.38%
25.21% 10.68% 1.28%
0.00% 0.00% 5.98% 0.00% 0.00% 5.98%
0.43% 17.52% 19.23% 0.00% 5.98% 31.20%
36.75% 19.66% 0.43% 26.50% 23.50% 6.84%
Occupied Palestinian Territory
0.00% 0.03% 7.01%
0.68% 9.26% 11.03%
55.99% 14.82% 1.19%
0.06% 2.80% 5.95% 0.03% 0.32% 8.45%
14.43% 13.60% 1.09% 4.69% 14.08% 10.35%
57.51% 4.56% 0.00% 51.94% 9.71% 0.42%
Palau
0.38% 1.00% 4.51%
5.01% 10.15% 5.26%
66.17% 6.02% 1.50%
0.00% 0.00% 0.00% 0.00% 0.00% 0.00%
0.00% 0.00% 0.50% 0.00% 0.00% 0.50%
73.68% 20.43% 5.39% 71.55% 17.17% 10.78%
Framingham
SCORE2
WHO
High
Middle
Low
value
100
75
50
25
0

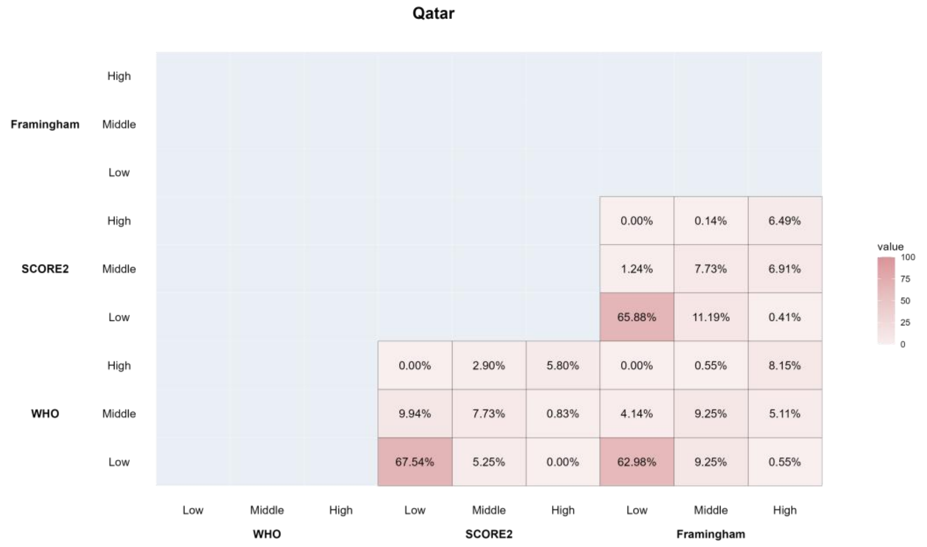
Qatar
Framingham
SCORE2
WHO
High
Middle
Low
0.00% 0.14% 6.49%
1.24% 7.73% 6.91%
65.88% 11.19% 0.41%
0.00% 2.90% 5.80% 0.00% 0.55% 8.15%
9.94% 7.73% 0.83% 4.14% 9.25% 5.11%
67.54% 5.25% 0.00% 62.98% 9.25% 0.55%
value
100 75 50 25 0

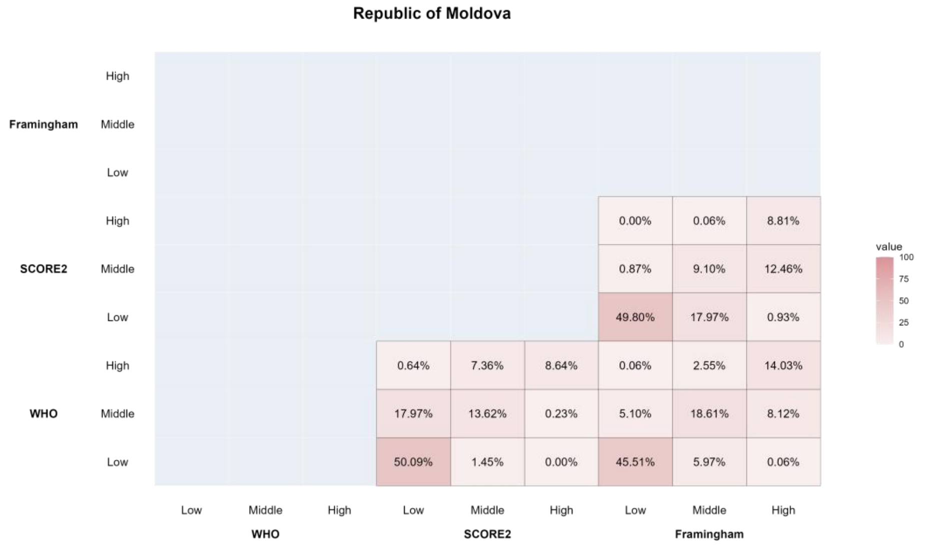
Republic of Moldova
Framingham
SCORE2
WHO
High
Middle
Low
0.00% 0.06% 8.81%
0.87% 9.10% 12.46%
49.80% 17.97% 0.93%
0.64% 7.36% 8.64% 0.06% 2.55% 14.03%
17.97% 13.62% 0.23% 5.10% 18.61% 8.12%
50.09% 1.45% 0.00% 45.51% 5.97% 0.06%
value
100 75 50 25 0

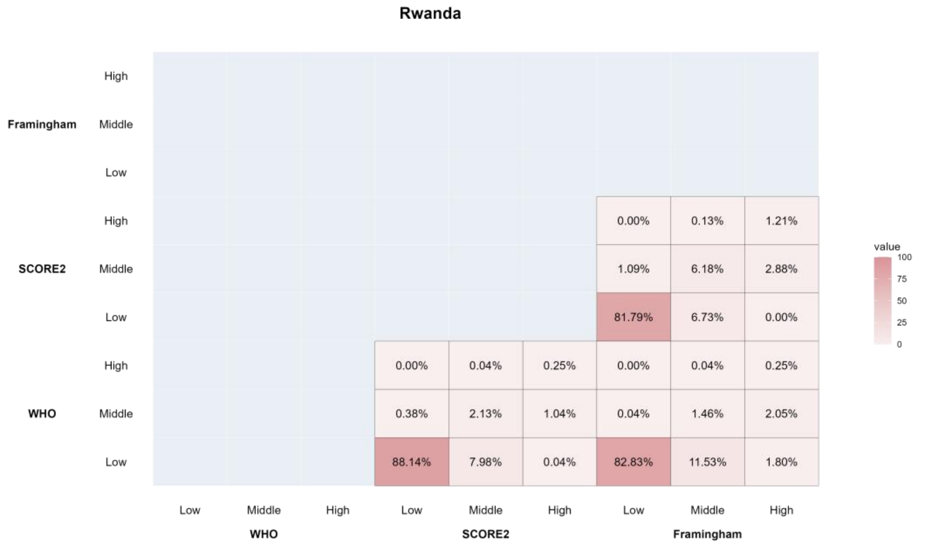
Rwanda
Framingham
SCORE2
WHO
High
Middle
Low
0.00% 0.13% 1.21%
1.09% 6.18% 2.88%
81.79% 6.73% 0.00%
0.00% 0.04% 0.25% 0.00% 0.04% 0.25%
0.38% 2.13% 1.04% 0.04% 1.46% 2.05%
88.14% 7.98% 0.04% 82.83% 11.53% 1.80%
value
100 75 50 25 0

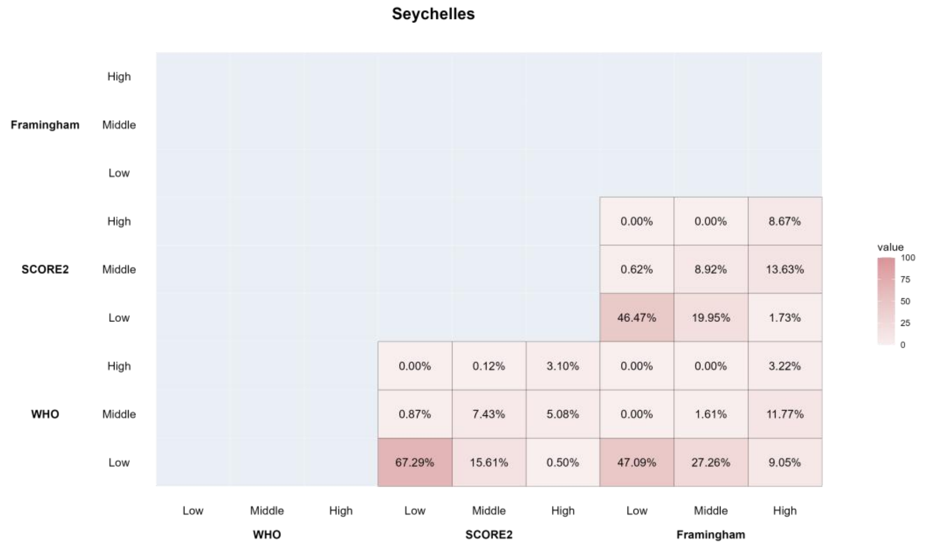
Seychelles
Framingham
SCORE2
WHO
High
Middle
Low
0.00% 0.00% 8.67%
0.62% 8.92% 13.63%
46.47% 19.95% 1.73%
0.00% 0.12% 3.10% 0.00% 0.00% 3.22%
0.87% 7.43% 5.08% 0.00% 1.61% 11.77%
67.29% 15.61% 0.50% 47.09% 27.26% 9.05%
value
100 75 50 25 0

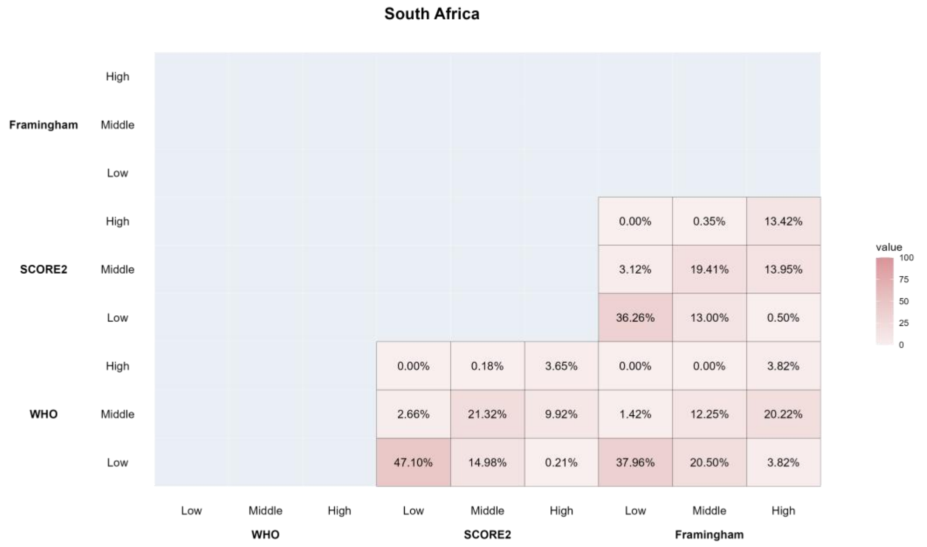
South Africa
Framingham
SCORE2
WHO
High
Middle
Low
0.00% 0.35% 13.42%
3.12% 19.41% 13.95%
36.26% 13.00% 0.50%
0.00% 0.18% 3.65% 0.00% 0.00% 3.82%
2.66% 21.32% 9.92% 1.42% 12.25% 20.22%
47.10% 14.98% 0.21% 37.96% 20.50% 3.82%
value
100 75 50 25 0

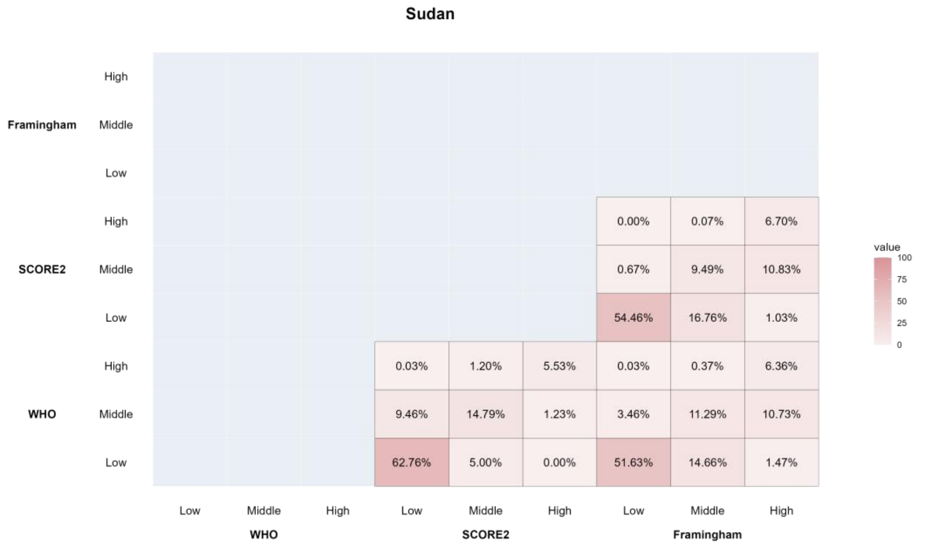
Sudan
Framingham
SCORE2
WHO
High
Middle
Low
0.00% 0.07% 6.70%
0.67% 9.49% 10.83%
54.46% 16.76% 1.03%
0.03% 1.20% 5.53% 0.03% 0.37% 6.36%
9.46% 14.79% 1.23% 3.46% 11.29% 10.73%
62.76% 5.00% 0.00% 51.63% 14.66% 1.47%
value
100 75 50 25 0

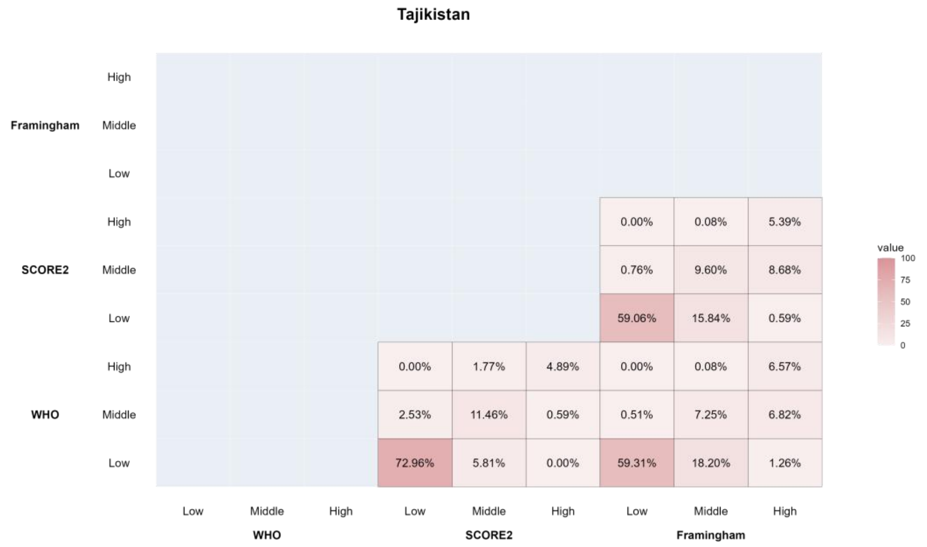
Tajikistan
Framingham
SCORE2
WHO
High
Middle
Low
0.00% 0.08% 5.39%
0.76% 9.60% 8.68%
59.06% 15.84% 0.59%
0.00% 1.77% 4.89% 0.00% 0.08% 6.57%
2.53% 11.46% 0.59% 0.51% 7.25% 6.82%
72.96% 5.81% 0.00% 59.31% 18.20% 1.26%
value
100 75 50 25 0

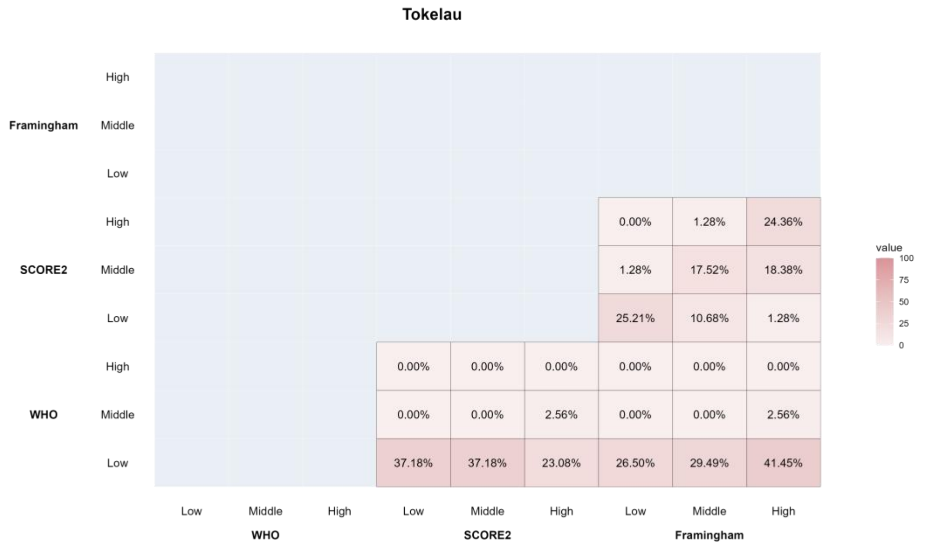
Tokelau
Framingham
SCORE2
WHO
High
Middle
Low
0.00% 1.28% 24.36%
1.28% 17.52% 18.38%
25.21% 10.68% 1.28%
0.00% 0.00% 0.00% 0.00% 0.00% 0.00%
0.00% 0.00% 2.56% 0.00% 0.00% 2.56%
37.18% 37.18% 23.08% 26.50% 29.49% 41.45%
value
100 75 50 25 0

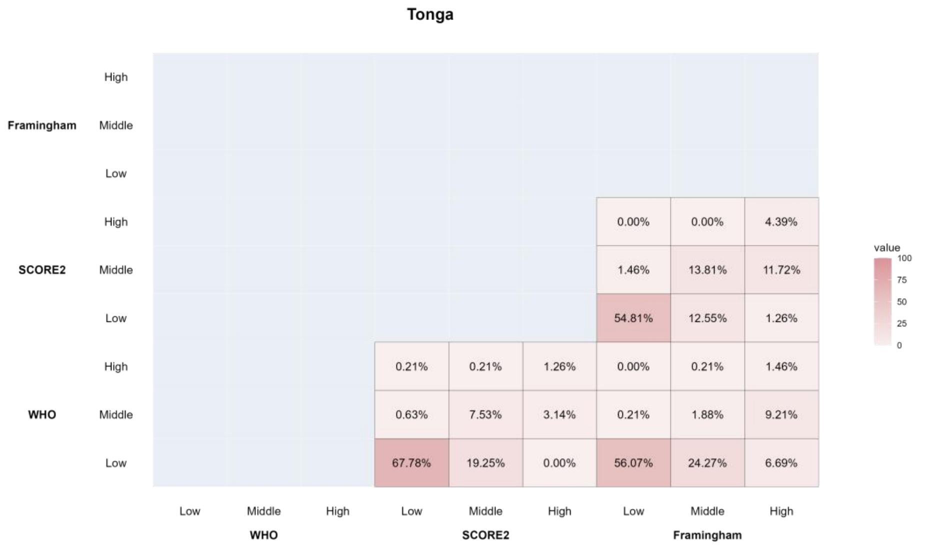
Tonga
Framingham
SCORE2
WHO
High
Middle
Low
0.00% 0.00% 4.39%
1.46% 13.81% 11.72%
54.81% 12.55% 1.26%
0.21% 0.21% 1.26% 0.00% 0.21% 1.46%
0.63% 7.53% 3.14% 0.21% 1.88% 9.21%
67.78% 19.25% 0.00% 56.07% 24.27% 6.69%
value
100 75 50 25 0

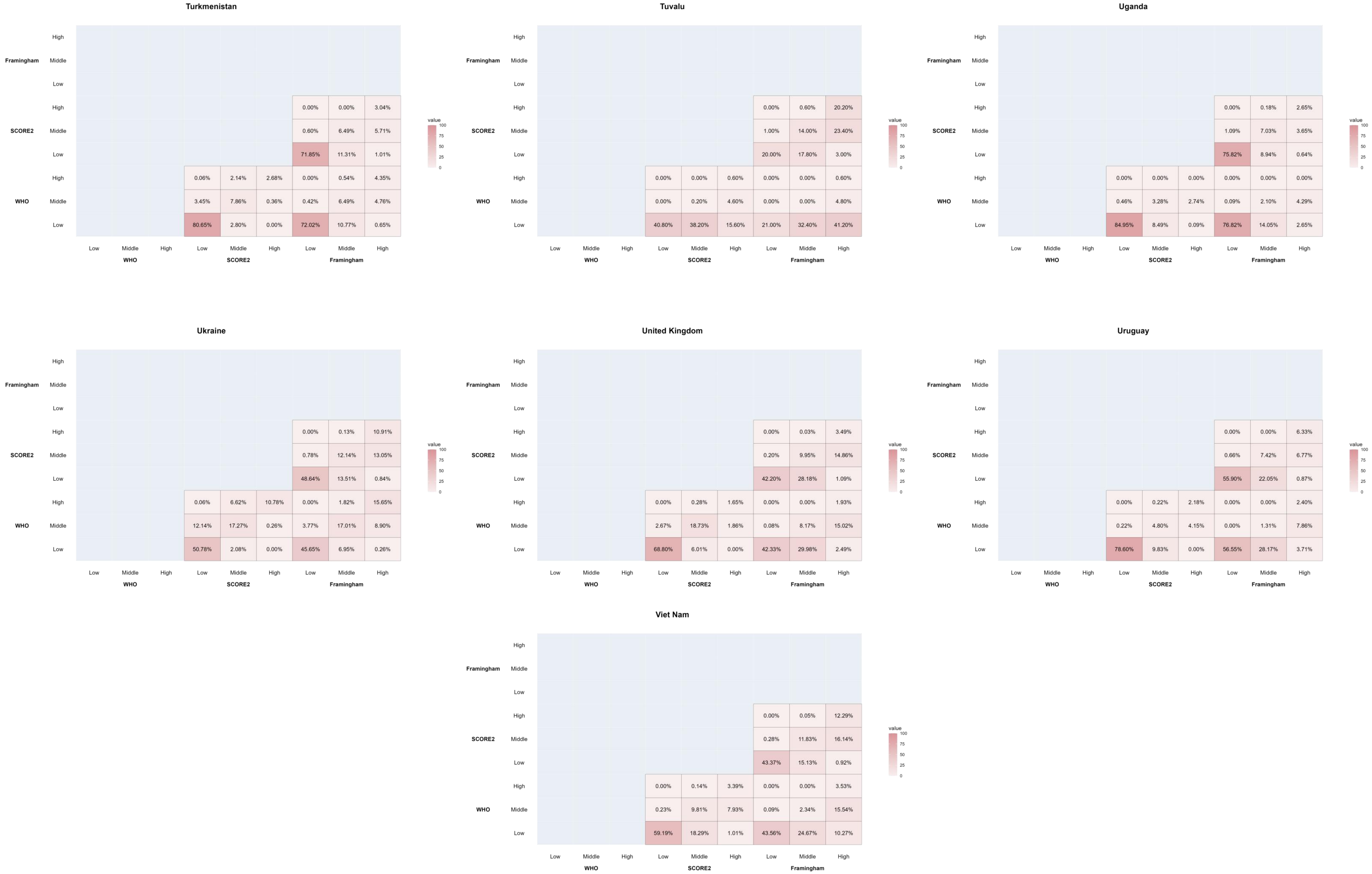

Figure S4. Pairwise consistency proportions of three CVD risk assessment models in classifying risk levels across regions and countries

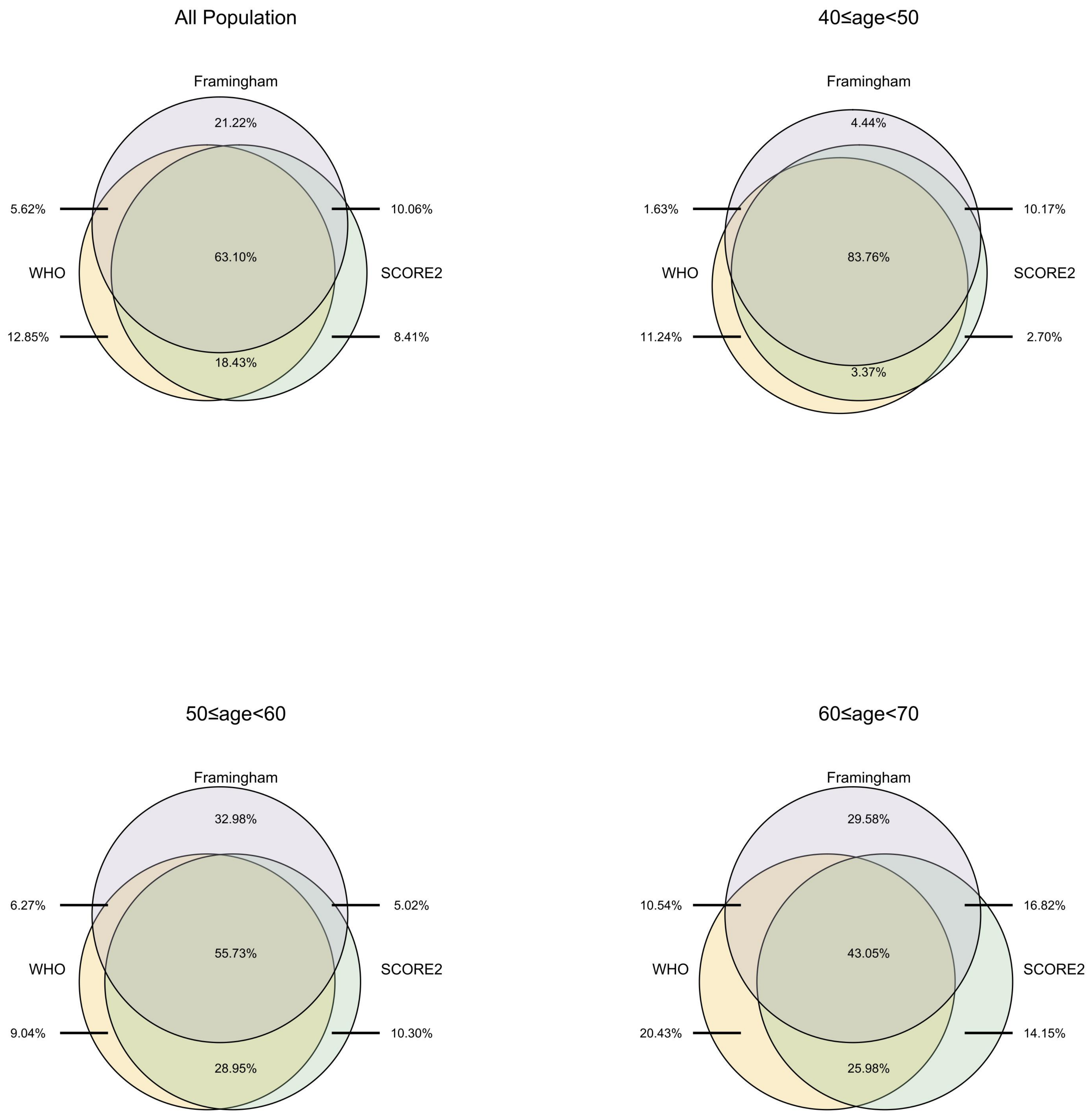

Figure S5. Consistency of three CVD risk assessment models in classifying low, moderate, and high risk levels across age levels

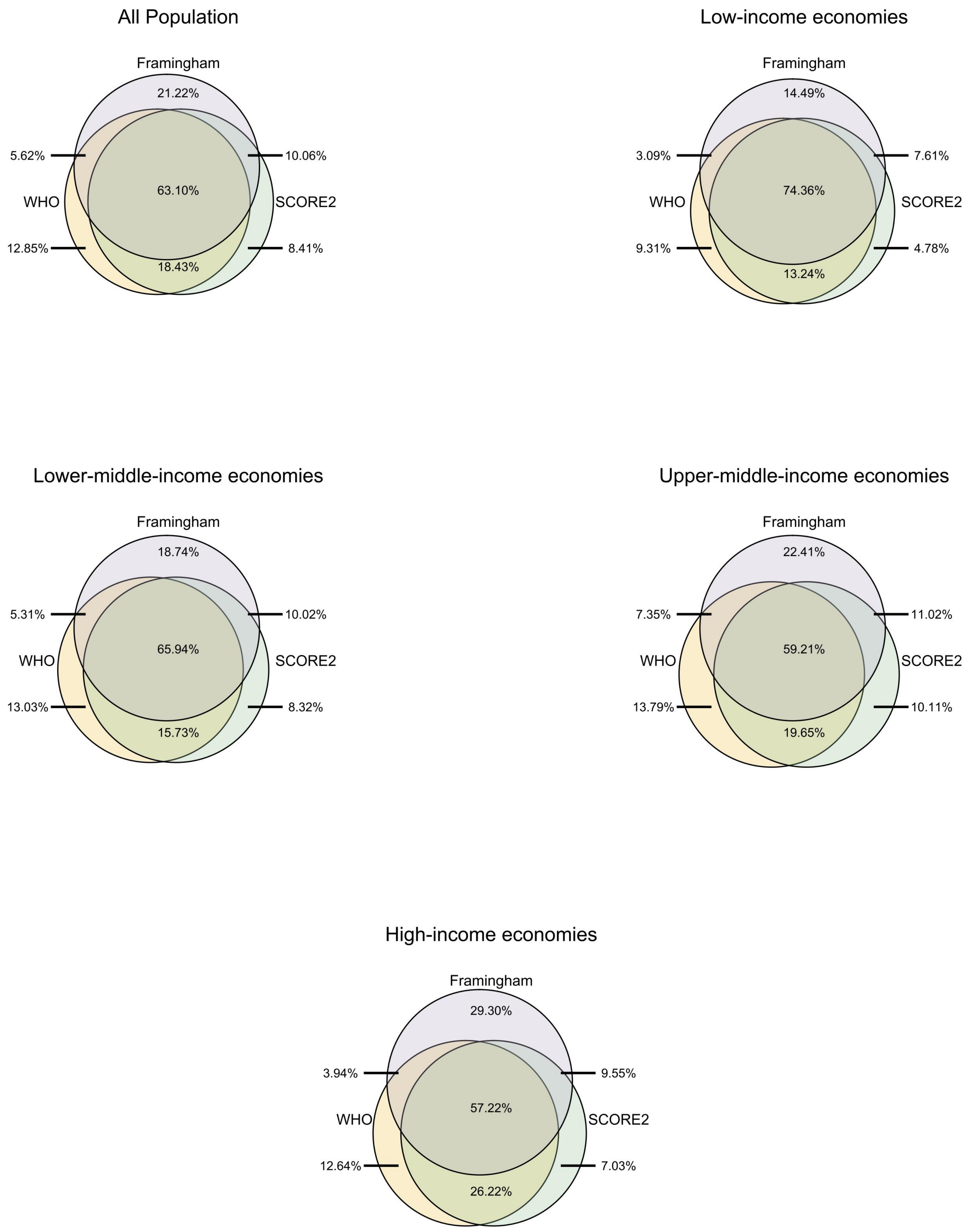

Figure S6. Consistency of three CVD risk assessment models in classifying low, moderate, and high risk levels across income levels

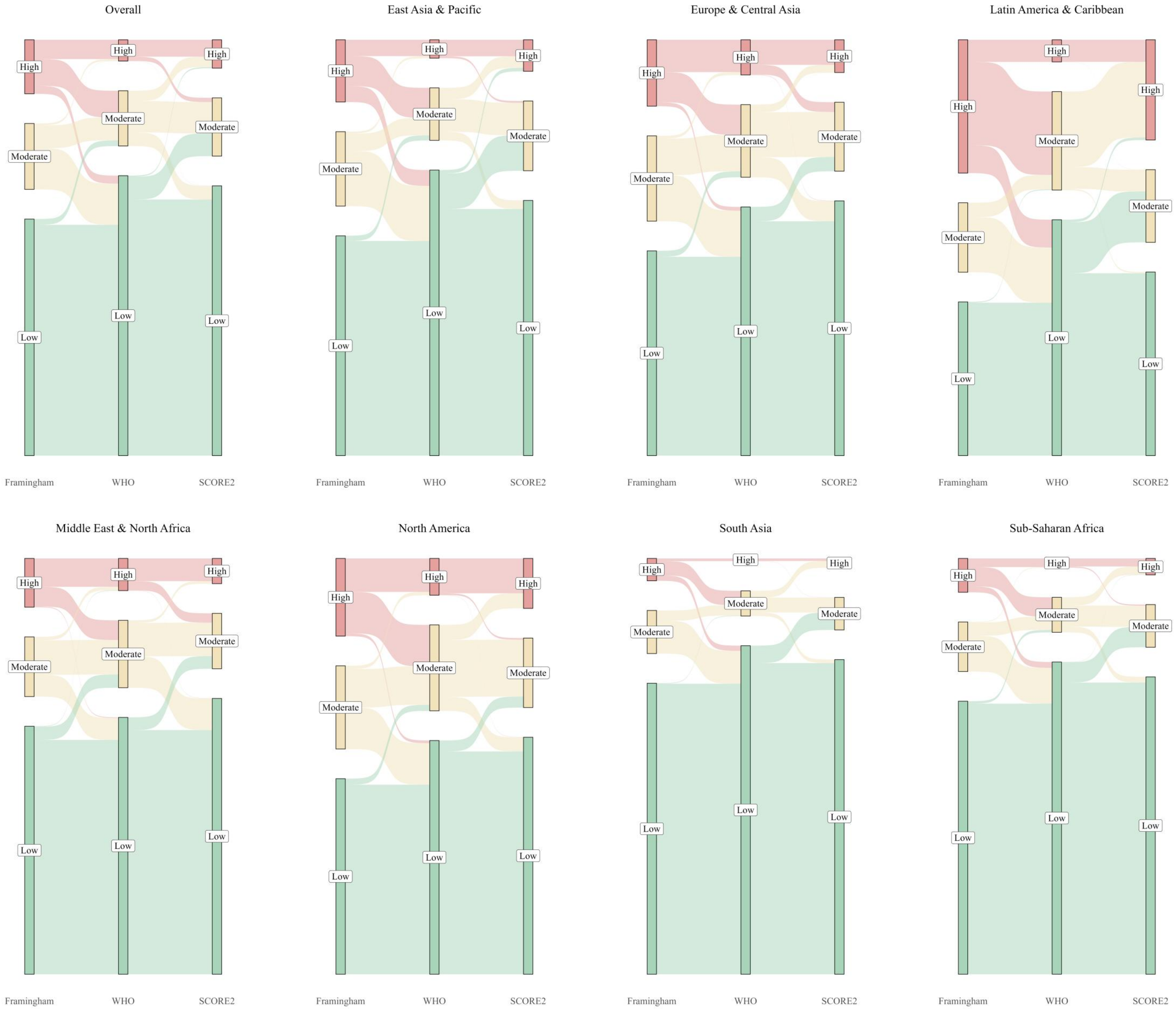

Figure S7. Sensitivity analysis of the distribution of 10-year CVD risk levels across regions (age 30–80 years)

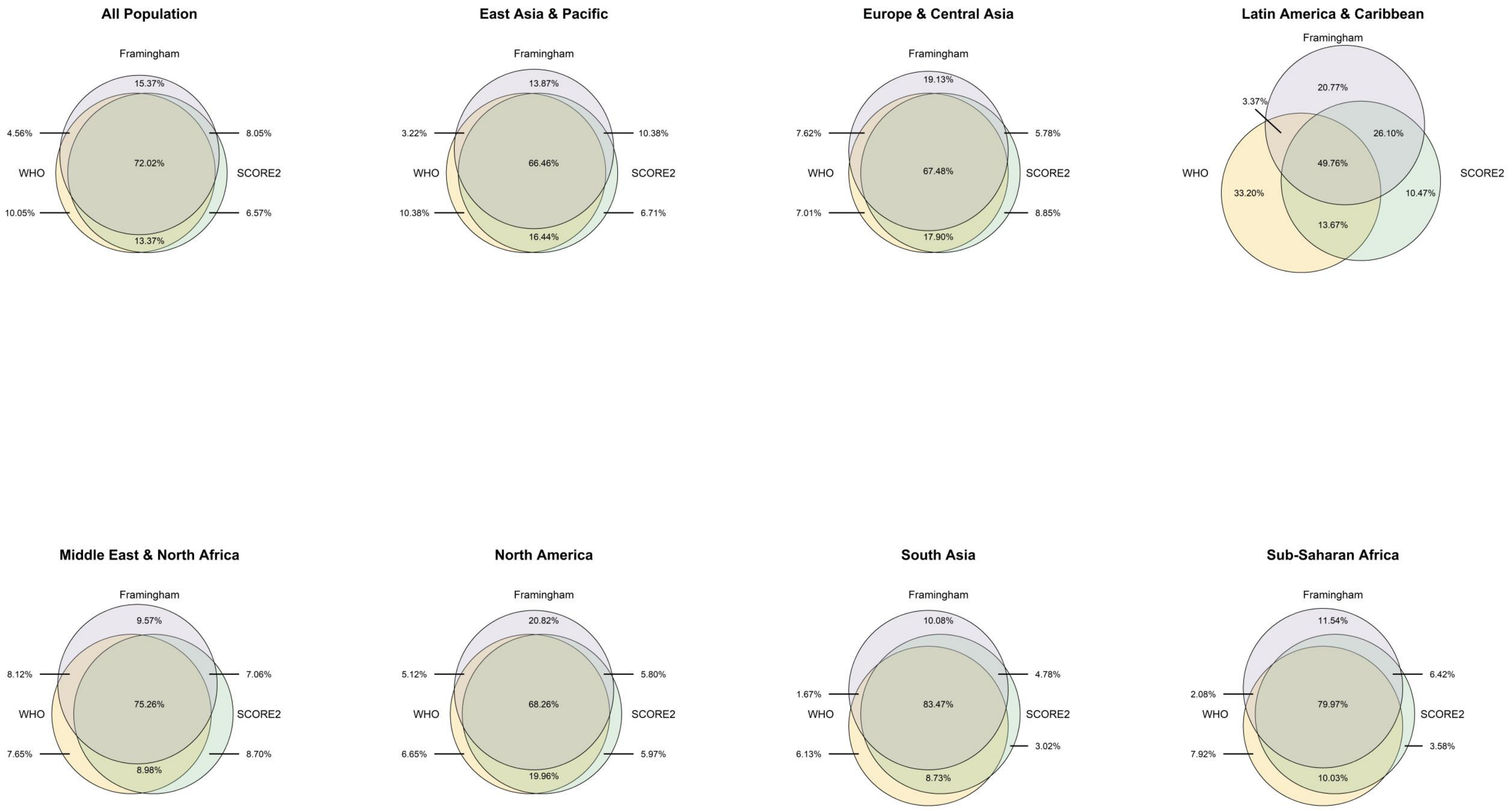

Figure S8. Sensitivity analysis of the consistency of three CVD risk assessment models in classifying low, moderate, and high risk levels across regions (age 30–80 years)

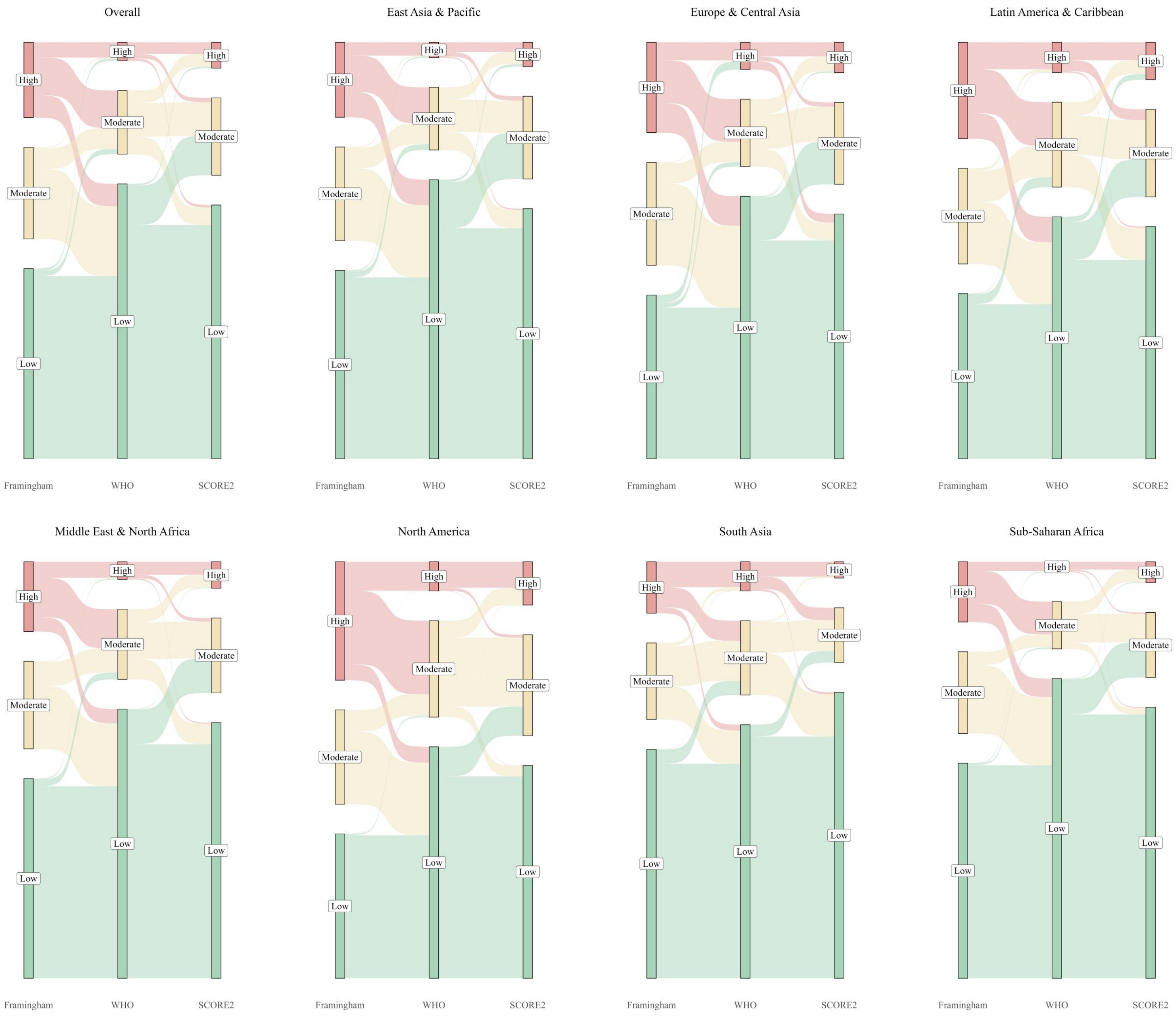

Figure S9.Sensitivity analysis of the distribution of 10-year CVD risk levels across regions (with multiple imputation)

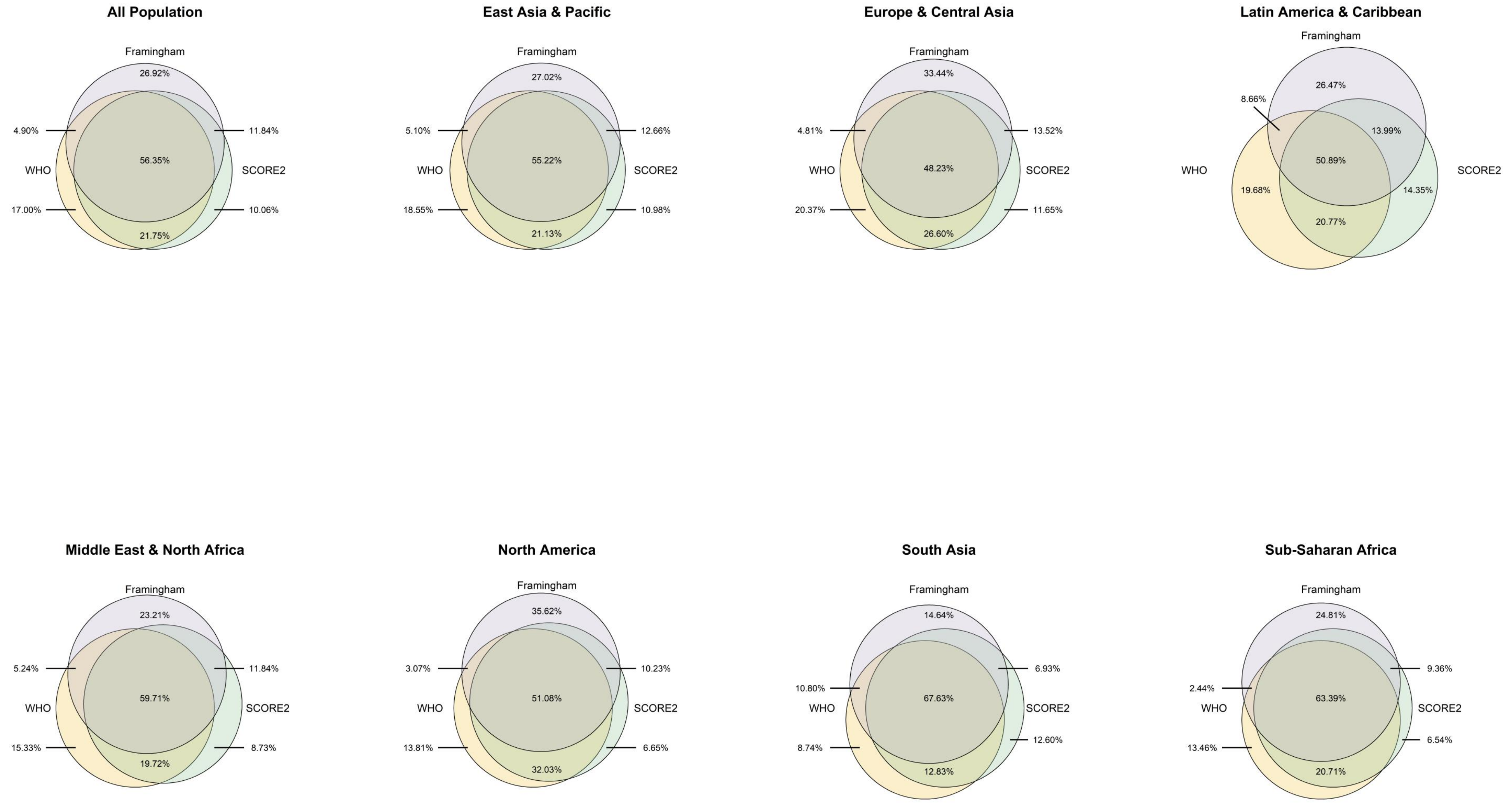

Figure S10.Sensitivity analysis of the consistency of three CVD risk assessment models in classifying low, moderate, and high risk levels across regions (with multiple imputation)

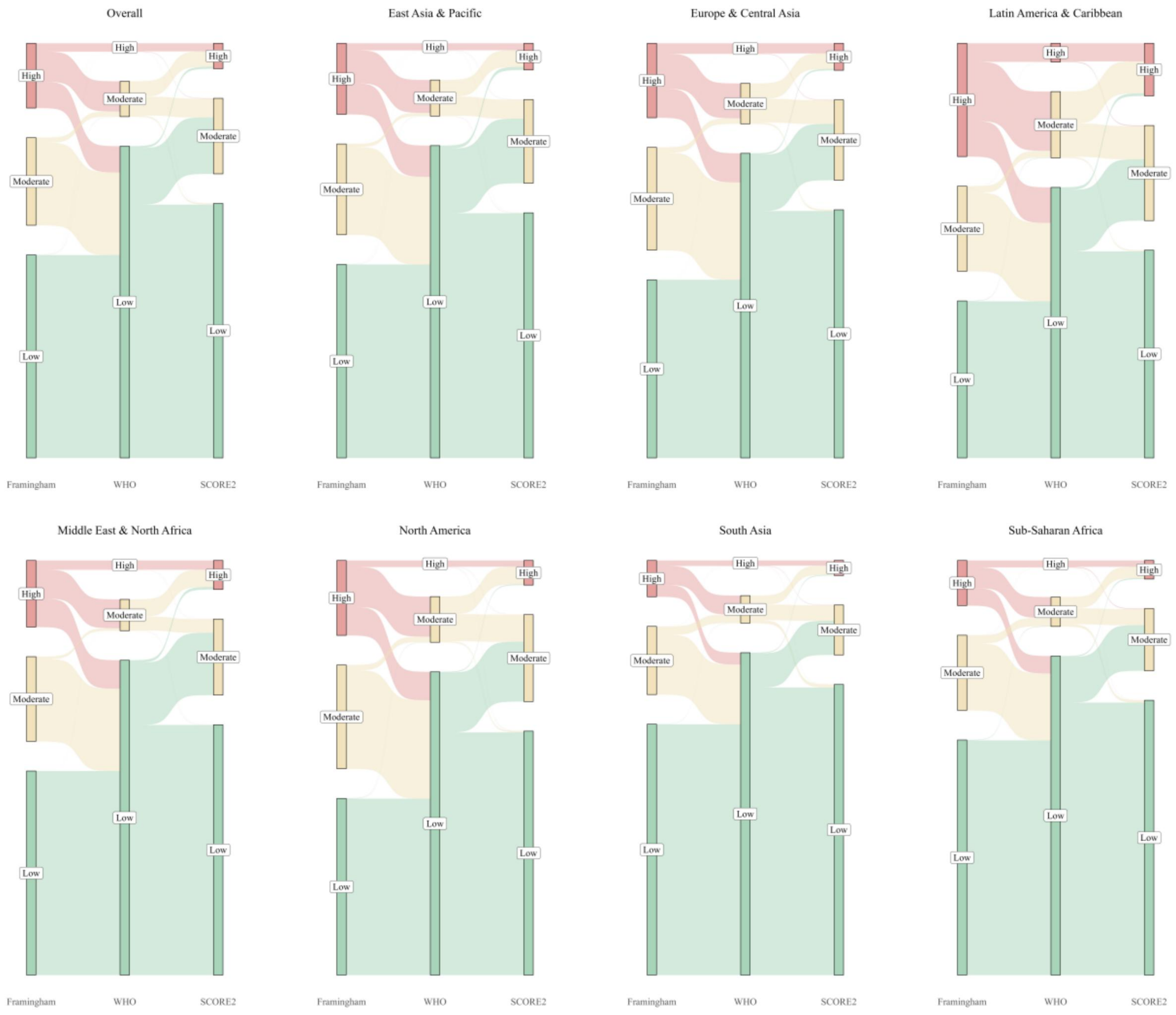

Figure S11.Sensitivity analysis of the distribution of 10-year CVD risk levels across regions (without regional recalibration)

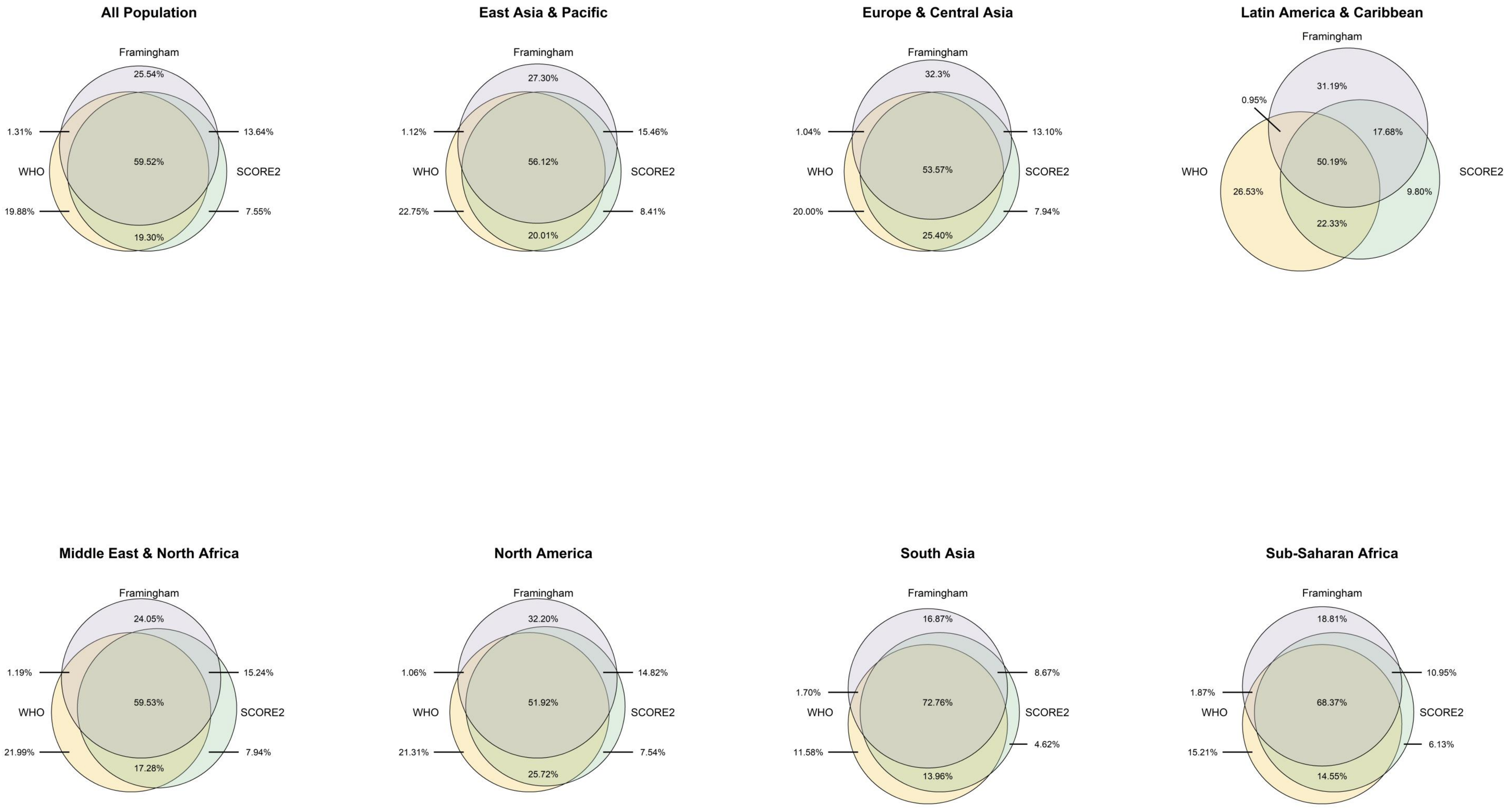

Figure S12.Sensitivity analysis of the consistency of three CVD risk assessment models in classifying low, moderate, and high risk levels across regions (without regional recalibration)

Reference:

1. STEPwise approach to NCD risk factor surveillance (STEPS). Accessed November 6, 2024. https://www.who.int/teams/noncommunicable-diseases/surveillance/systems-tools/steps

2. NatCen Social Research, University College London, Institute for Fiscal Studies. English Longitudinal Study of Ageing. 7th Release. Published online 2023. doi:http://doi.org/10.5255/UKDA-Series-200011

3. HAALSA. Accessed November 6, 2024. https://haalsi.org/home

4. Wong R, Michaels-Obregon A, Palloni A. Cohort Profile: The Mexican Health and Aging Study (MHAS). *Int J Epidemiol*. 2017;46(2):e2. doi:10.1093/ije/dyu263

5. Costa Rica: Estudio de Longevidad y Envejecimiento Saludable. Accessed November 6, 2024. http://creles.berkeley.edu/

6. MIDUS - Midlife in the United States, A National Longitudinal Study of Health and Well-being. Accessed November 6, 2024. https://www.midus.wisc.edu/midja/index.php

7. CHARLS. Accessed November 6, 2024. https://charls.pku.edu.cn/en/

8. NHANES - National Health and Nutrition Examination Survey Homepage. November 4, 2024. Accessed November 6, 2024. https://www.cdc.gov/nchs/nhanes/index.htm
